# Validating a non-invasive, ALT-based non-alcoholic fatty liver phenotype in the million veteran program

**DOI:** 10.1371/journal.pone.0237430

**Published:** 2020-08-25

**Authors:** Marina Serper, Marijana Vujkovic, David E. Kaplan, Rotonya M. Carr, Kyung Min Lee, Qing Shao, Donald R. Miller, Peter D. Reaven, Lawrence S. Phillips, Christopher J. O’Donnell, James B. Meigs, Peter W. F. Wilson, Rachel Vickers-Smith, Henry R. Kranzler, Amy C. Justice, John M. Gaziano, Sumitra Muralidhar, Saiju Pyarajan, Scott L. DuVall, Themistocles L. Assimes, Jennifer S. Lee, Philip S. Tsao, Daniel J. Rader, Scott M. Damrauer, Julie A. Lynch, Danish Saleheen, Benjamin F. Voight, Kyong-Mi Chang, on behalf of the VA Million Veteran Program

**Affiliations:** 1 Corporal Michael J. Crescenz VA Medical Center, Philadelphia, Pennsylvania, United States of America; 2 Department of Medicine, Perelman School of Medicine, University of Pennsylvania, Philadelphia, Pennsylvania, United States of America; 3 Leonard Davis Institute of Health Economics, University of Pennsylvania, Philadelphia, Pennsylvania, United States of America; 4 Center for Healthcare Organization and Implementation Research, Edith Nourse Rogers Memorial Veterans Hospital, Bedford, Massachusetts, United States of America; 5 Department of Health Law, Policy and Management, Boston University School of Public Health, Boston, Massachusetts, United States of America; 6 VA Informatics and Computing Infrastructure, VA Salt Lake City Health Care System, Salt Lake City, Utah, United States of America; 7 Phoenix VA Health Care System, Phoenix, Arizona, United States of America; 8 Department of Veterans Affairs, Atlanta Health Care System, Decatur, Georgia, United States of America; 9 Division of Endocrinology and Metabolism, Department of Medicine, Emory University School of Medicine, Atlanta, Georgia, United States of America; 10 Massachusetts Veterans Epidemiology Research and Information Center, VA Boston Healthcare System, Boston, Massachusetts, United States of America; 11 Department of Medicine, Brigham and Women's Hospital, Harvard Medical School, Boston, Massachusetts, United States of America; 12 Massachusetts General Hospital, Harvard Medical School and the Broad Institute, Boston, Massachusetts, United States of America; 13 University of Louisville, Louisville, Kentucky, United States of America; 14 Department of Psychiatry, Perelman School of Medicine, University of Pennsylvania, Philadelphia, Pennsylvania, United States of America; 15 Yale School of Medicine, New Haven, Connecticut, United States of America; 16 Veterans Affairs Connecticut Healthcare System, West Haven, Connecticut, United States of America; 17 Yale School of Public Health, New Haven, Connecticut, United States of America; 18 Boston University School of Public Health, Boston, Massachusetts, United States of America; 19 Office of Research and Development, Veterans Health Administration, Washington, DC, United States of America; 20 Department of Internal Medicine Division of Epidemiology, University of Utah School of Medicine, Salt Lake City, Utah, United States of America; 21 Department of Medicine, Stanford University School of Medicine, Stanford, California, United States of America; 22 VA Palo Alto Health Care System, Palo Alto, California, United States of America; 23 Department of Genetics, Perelman School of Medicine, University of Pennsylvania, Philadelphia, Pennsylvania, United States of America; 24 Department of Pediatrics, Perelman School of Medicine, University of Pennsylvania, Philadelphia, Pennsylvania, United States of America; 25 Cardiovascular Institute, Perelman School of Medicine, University of Pennsylvania, Philadelphia, Pennsylvania, United States of America; 26 Department of Surgery, Perelman School of Medicine, University of Pennsylvania, Philadelphia, Pennsylvania, United States of America; 27 College of Nursing and Health Sciences, University of Massachusetts, Boston, Massachusetts, United States of America; 28 Department of Biostatistics and Epidemiology, Perelman School of Medicine, University of Pennsylvania, Philadelphia, Pennsylvania, United States of America; 29 Department of Systems Pharmacology and Translational Therapeutics and Genetics, Perelman School of Medicine, University of Pennsylvania, Philadelphia, Pennsylvania, United States of America; Wake Forest School of Medicine, UNITED STATES

## Abstract

**Background & aims:**

Given ongoing challenges in non-invasive non-alcoholic liver disease (NAFLD) diagnosis, we sought to validate an ALT-based NAFLD phenotype using measures readily available in electronic health records (EHRs) and population-based studies by leveraging the clinical and genetic data in the Million Veteran Program (MVP), a multi-ethnic mega-biobank of US Veterans.

**Methods:**

MVP participants with alanine aminotransferases (ALT) >40 units/L for men and >30 units/L for women without other causes of liver disease were compared to controls with normal ALT. Genetic variants spanning eight NAFLD risk or ALT-associated loci (*LYPLAL1*, *GCKR*, *HSD17B13*, *TRIB1*, *PPP1R3B*, *ERLIN1*, *TM6SF2*, *PNPLA3)* were tested for NAFLD associations with sensitivity analyses adjusting for metabolic risk factors and alcohol consumption. A manual EHR review assessed performance characteristics of the NAFLD phenotype with imaging and biopsy data as gold standards. Genetic associations with advanced fibrosis were explored using FIB4, NAFLD Fibrosis Score and platelet counts.

**Results:**

Among 322,259 MVP participants, 19% met non-invasive criteria for NAFLD. Trans-ethnic meta-analysis replicated associations with previously reported genetic variants in all but *LYPLAL1* and *GCKR* loci (P<6x10^-3^), without attenuation when adjusted for metabolic risk factors and alcohol consumption. At the previously reported *LYPLAL1* locus, the established genetic variant did not appear to be associated with NAFLD, however the regional association plot showed a significant association with NAFLD 279kb downstream. In the EHR validation, the ALT-based NAFLD phenotype yielded a positive predictive value 0.89 and 0.84 for liver biopsy and abdominal imaging, respectively (inter-rater reliability (Cohen’s kappa = 0.98)). *HSD17B13* and *PNPLA3* loci were associated with advanced fibrosis.

**Conclusions:**

We validate a simple, non-invasive ALT-based NAFLD phenotype using EHR data by leveraging previously established NAFLD risk-associated genetic polymorphisms.

## Introduction

Non-alcoholic fatty liver disease (NAFLD) is a heritable, clinically heterogeneous disorder encompassing simple steatosis and non-alcoholic steatohepatitis (NASH) with concomitant cardio-metabolic risk factors [1, 2]. To date, genome-wide association studies (GWAS) for NAFLD and related traits such as serum alanine aminotransferase (ALT) concentration have identified 8 independent genetic loci derived primarily from hepatic lipid and glucose homeostatic genes (*LYPLAL1*, *GCKR*, *HSD17B13*, *TRIB1*, *PPP1R3B*, *CPN1-ERLIN1-CHUK*, *TM6SF2*, *PNPLA3*) (S1 Table in S1 File) [3–18]. In particular, the I148M variant of the patatin-like phospholipase domain-containing protein-3 (*PNPLA3*) gene has been strongly associated with NAFLD, ALT concentration, and alcoholic liver disease. *PNPLA3* encodes the calcium-independent phospholipase A2 epsilon (also called adiponutrin) which is enriched in hepatocytes and hepatic stellate cells and has a role in lipid droplet regulation [19]. Additionally, polymorphisms in *MBOAT7 and IFNL3/4* have been shown to be associated with hepatic steatosis and necroinflammation [20–22].

Despite our advanced understanding of NAFLD pathogenesis, population-based identification of NAFLD remains a challenge in clinical practice and research [23, 24]. Although liver biopsy is generally considered the gold standard in NAFLD diagnosis [25], it is infrequently performed in routine clinical care due to its invasive nature with poor patient acceptance and sample variability [26]. Conventional ultrasound, though frequently used, has limited sensitivity and specificity, whereas the role of transient elastography continues to emerge [1]. While magnetic resonance imaging (MRI) modalities such as MRI protein-density fat fraction (MRI-PDFF) or Magnetic Resonance Spectroscopy (MRS) can accurately diagnose hepatic steatosis, these technologies are not widely available in routine clinical practice [26, 27]. Current electronic-health record (EHR) based algorithms using diagnosis codes, clinical encounters, and laboratory values have limited sensitivity, underestimate population prevalence, and still require clinician adjudication and labor-intensive medical record review [24, 28, 29]. Additional approaches to NAFLD phenotyping such as natural language processing and machine learning remain areas of active and ongoing investigation [30].

Given these challenges in NAFLD diagnosis, we sought to validate a phenotype of NAFLD using measures that can be readily applied in clinical practice and in population-based investigations. To this end, we leveraged robust clinical and genomic data from the Million Veteran Program (MVP), a multi-ethnic cohort with over 300,000 genotyped Veterans enrolled at 63 Veteran Affairs (VA) medical centers across the United States (US) [31]. Specifically, we used 16 genetic variants from 8 previously reported independent loci associated with NAFLD risk (diagnosed using imaging, liver biopsy and related traits) and EHR review to validate a clinical NAFLD phenotype. The replication of known genetic variant associations was performed in MVP to increase the confidence on the non-invasive ALT-based NAFLD phenotype to facilitate future genetic association studies.

## Materials and methods

### MVP cohort description

This was a cross-sectional analysis at the time of MVP enrollment using previously collected EHR data. We performed replication analyses using DNA samples and clinical data from the MVP cohort, which has been described previously in detail [31, 32]. All participants provided written informed consent to participate in the study. Consented participants provided a blood sample, answered self-reported baseline and lifestyle questionnaires, and were consented for future contact. Recruitment is ongoing at 63 VA Medical Centers across the US. The cohort is predominantly male and enriched with Veterans of African (AA) and Hispanic/Latino (LA) ancestry as compared to the US population [31]. Prospectively collected questionnaire data were linked with clinical information from the VA EHR via the VA’s central database, the Corporate Data Warehouse (CDW). The MVP core study protocol was approved by the VA Central Institutional Review Board (CIRB) and the Research and Development (R&D) Committees at all 63 participating VA medical centers. Further approval for this specific analysis was obtained from the VA CIRB and from the R&D committees at Bedford, Philadelphia, Palo Alto, Salt Lake City, and Phoenix VA medical centers.

For the current analysis, clinical and genetic data were available from 234,683 European (EU), 64,961 AA, and 22,615 LA participants (S2 Table in S1 File) categorized as mutually-exclusive ancestral groups based on CDW data, self-identified race/ethnicity, and genetically inferred ancestry enrolled in MVP from 2011 until 2016 [33]. Asian American participants were excluded due to small sample size. As shown in S2 Table in S1 File, we further excluded 71,012 participants with the presence of international classification of disease-clinical modification (ICD-9-CM/10-CM) codes for alcoholic liver disease and/or alcohol use disorder (n = 51,549), other chronic viral (n = 7,995) metabolic, cholestatic liver diseases and liver metastases (n = 11,468). For the main analyses, we further excluded 58,631 participants with intermediate ALT values (between 30–40 U/L for men and 20–30 U/L for women) that did not meet threshold ALT cutoffs for NAFLD case or control phenotype, resulting in a final analytic cohort of 192,616 (S2 Table in S1 File, Row C).

### NAFLD phenotype definitions

MVP NAFLD phenotype definitions were developed by combining a previously published VA CDW ALT-based approach [24] with non-invasive clinical parameters available to practicing clinicians at the point of care. The primary NAFLD phenotype (“ALT-threshold”) was defined by: (i) elevated ALT >40 U/L for men and >30 U/L for women during at least two time points at least 6 months apart within a two-year window period at any point prior to enrollment and (ii) exclusion of other causes of liver disease (e.g. viral, cholestatic, and hereditary in addition to alcohol-related hepatitis and cirrhosis) and/or alcohol use disorder by ICD-9-CM/10-CM. Another ALT-based phenotype, ABALT, defined as ALT >30 U/L for men, >20 U/L for women was evaluated using EHR validation (**EHR validation section**).

A secondary NAFLD phenotype (“ALT-metabolic”) combined “ALT-threshold” criteria and at least one metabolic risk factor including obesity with body mass index (BMI) ≥ 30 kg/m^2^, dyslipidemia (DL), type 2 diabetes mellitus (T2D) or pre-diabetes as defined in the **Metabolic Risk Factor** section below. The control group was defined by: normal ALT (≤30 U/L for men, ≤20 U/L for women) and no apparent causes of liver disease. There was a 97% overlap between NAFLD cohorts defined by ALT-threshold and ALT-metabolic phenotypes. Given this high overlap, we chose “ALT-threshold” as the main NAFLD phenotype for our analyses given its simplicity and applicability in diverse study settings where clinical data may not be as detailed as in the VA CDW.

We examined the associations between known ALT-associated variants and maximum ALT within 2 years prior to enrollment as a continuous variable (labeled “ALT-max”). Sensitivity analyses were conducted with six additional NAFLD phenotypes as defined in S4 Table in S1 File in which we altered ALT thresholds, individual metabolic risk factors, and inclusion of intermediate ALT values in the control group (S5, S6 Tables in S1 File).

### Metabolic risk factor definitions

All baseline variables were created using the most recent observation prior to MVP enrollment. BMI was obtained from vital signs taken during clinical appointments. DL was defined as any of the following: (i) triglyceride (TG) ≥ 150 mg/dL taken before 9 AM, (ii) high density lipoprotein (HDL) cholesterol < 40 mg/dL for men and < 50 mg/dL for women with at least 2 ICD-9-CM/10-CM codes (272.x/E78.0-E78.5), or (iii) at least one prescription for fenofibrate or gemfibrozil. The DL definition was based on the criteria established by Third Adult Treatment Panel (NCEP ATP III) for diagnosis of metabolic syndrome (MetS) [34]. Patients prescribed HMG-CoA reductase inhibitors who did not meet any other criteria were not classified as having DL as they could have been prescribed statins for primary coronary artery disease prevention unrelated to dyslipidemia [35]. Hypertension (HTN) was defined by ICD-9-CM/10-CM codes (401.x-405.x/I10-I16).

T2D was based on any of the following criteria: (i) ICD9-10 codes shown in S3 Table in S1 File, but excluding codes for diabetes mellitus (T1D), other diabetes, medical conditions that may cause diabetes, or diabetes pattern consistent with T1D (which included insulin in the absence of oral agents, age of onset <40 years, BMI<25, or history of diabetic ketoacidosis), (ii) hemoglobin A1c (HbA1c) ≥6.5% or outpatient blood glucose of ≥200 mg/dL, or (iii) at least two prescriptions for diabetic medications. Pre-diabetes was defined with ICD-9/ICD-10-CM codes: 790.2, 790.2x except 790.29, R73, R73.xx except R73.03 or HbA1c between 5.7% and 6.49%, ever before the enrollment date in the absence of diabetes.

### Assessment of alcohol use

Alcohol consumption was assessed with the mean age-adjusted scores from the Alcohol Use Disorders Identification Test-Consumption (AUDIT-C), a validated 3-item questionnaire administered annually by VA primary care practitioners and used previously in MVP [36–38]. The rationale for including and adjusting for AUDIT-C was: i) diagnostic codes used to exclude patients for alcohol-use disorder may be insensitive for mild to moderate alcohol consumption, ii) one third of the sample met criteria for possible alcohol misuse by AUDIT-C resulting in loss of power if applying AUDIT-C as an exclusion criterion.

### Genetic data

DNA extracted from whole blood was genotyped in MVP using a customized Affymetrix Axiom biobank array, the MVP 1.0 Genotyping Array, as previously described [31, 32]. Quality control procedures include the following as previously reported: 1) ancestry classification using a composite of self-reported race/ethnicity followed by ADMIXTURE v1.3 analyses; 2) exclusion of low-quality samples (individual missingness >2.5%), 3) exclusion of related samples (using KING software); and 4) exclusion of low quality variants (<95% call rate) [32]. Subsequently, genome-wide genotype pre-phasing (EAGLE v2) and imputation (Minimac3) was performed using the 1000 Genomes phase 3, version 5 reference population where variants with posterior call probability of < 0.9, imputation quality score <0.3, call rate <97.5%, and/or ancestry specific Hardy-Weinberg equilibrium P <1x10^-20^ were excluded. Variants were also excluded if they deviated >10% from their expected allele frequency from the 1000 Genomes Project. Ethnicity-specific principal component analysis was performed using EIGENSOFT software.

### Genetic variants selected for analyses

As shown in S1 Table in S1 File, we initially tested 15 genetic variants representing 8 independent genomic regions from the imputed genetic dataset that were previously identified in genome-wide association studies [3–9], including those associated with ALT concentration [3, 7, 8] and/or NAFLD diagnosed by MR spectroscopy [9, 39], computed tomography (CT) [6], and histology [3, 40]. After this initial analysis (and lack of association at LYPLAL1), regional association plots were generated for all 8 previously reported NAFLD-associated loci using LocusZoom software [41] and shown in S1 Fig.

### Electronic health record review

A medical record review in the VA EHR was independently performed by two hepatologists on a sample of national data of 457 MVP enrollees, that included 241 with liver biopsies and 216 that had at least one abdominal ultrasound, CT scan, or MRI to assess the diagnostic performance of the two ALT-based NAFLD phenotype definitions against biopsy-proven and/or radiologically confirmed NAFLD: (i) ABALT and (ii) ALT-threshold both defined above (**NAFLD Phenotype Definitions**). In addition to liver biopsy and imaging data, the adjudicators reviewed laboratory parameters, diagnoses, medication lists and inpatient and outpatient clinical notes to rule in or out NAFLD; the algorithm followed a previously published schema in the Veteran population [24]. The inter-rater reliability was measured by Cohen’s kappa (κ) statistic. Performance characteristics of two NAFLD phenotypes, ALT-threshold and ABALT, against EHR-adjudicated NAFLD as the gold standard were assessed by calculating positive predictive values (PPVs) using Stata 15 (StataCorp LP, College Station, TX).

### Assessment of advanced liver disease

We investigated relationships between previously established NAFLD variants and advanced liver disease using two established clinically defined scores: FIB4 = Age [years] x AST [U/L] / (platelets [10^9/L] x sqrt (ALT)), and NAFLD fibrosis score = -1.675 + (0.037*age) + (0.094*BMI) + (1.13*(diabetes or prediabetes as defined above)) + (0.99*(AST/ALT))–(0.013*platelets)–(0.66*albumin) [26, 42–45]. We defined advanced liver disease phenotypes at enrollment by: (i) FIB4 score >2.670 [44] and (ii) NAFLD fibrosis score >0.676 with cutoffs based on their optimal performance characteristics in previous NAFLD studies [43]. Average platelet count at enrollment was investigated as a surrogate for portal hypertension as a continuous measure. We also analyzed FIB4 and NAFLD fibrosis scores as continuous measures (S7 Table in S1 File).

### Statistical analyses

Regression models were used to delineate the presence and strength of the relationship between 8 established genetic loci and various definitions of the NAFLD phenotype (i.e. ABALT, ABALT2, ALT2DL, AL2DM, ALT2HTN, ALT2OBESE, FIB4score, NAFLD fibrosis score and their definition is described in S4 Table in S1 File). A total of 16 genetic variants were chosen to represent 8 independent genetic regions. In particular, the 15 previously reported variants (described in S1 Table in S1 File) were chosen together with an additional variant in LYPLA1 locus (rs3001032, chr1:219727779) that captured the lead association with NAFLD in the Million Veteran Program dataset upon investigating the regional association plot (S1 Fig). Linear regression was used for continuous outcomes, such as FIB4 score, NAFLD fibrosis score, whereas logistic regression was performed for dichotomous outcomes, e.g. ABALT, ABALT2, ALT2DL, AL2DM, ALT2HTN, ALT2OBESE. The primary analysis for the three above phenotypes was a trans-ethnic meta-analysis combining participants of EU, AA, and LA ancestry; this was also conducted separately for each ancestry (S5A–S5C Table in S1 File). The meta-analyses were performed using in a fixed-effects model using METAL with inverse-variance weighting of log odds ratios [46]. Between-study allelic effect size heterogeneity was assessed with Cochran’s Q statistic as implemented in METAL. Variants were considered genome-wide significant if they surpassed the standard threshold (P = 5x10^-8^). Additional replication-level significance (of P = 0.00625 representing Bonferroni correction of 8 independent loci) and experiment-wide significance (P = 1x10^-5^ for correction of ~5,000 independent tests regionally across the 8 loci) were also considered. Three multivariable models were generated for each outcome: (i) **Model 1**: NAFLD phenotype modeled as a function of SNP, age, gender, and the first 10 genetic principal components (PCs) of genetic ancestry, (ii) **Model 2**: NAFLD phenotype modeled as a function of SNP, age, gender, the first 10 genetic principal components, and alcohol consumption at enrollment, and (iii) **Model 3**: NAFLD phenotype modeled as a function of age, gender, the first 10 genetic principal components, alcohol consumption, T2D, hypertension, dyslipidemia and obesity. Covariates included age, gender, AUDIT-C score, and 10 PCs for genetic similarity. Analysis was performed using R version 3.2.5.

## Results and discussion

### Characteristics of NAFLD analytic cohort across diverse ancestries

As shown in Table 1, 192,616 participants in the final NAFLD analytic cohort included 148,354 (82%) Europeans (EU), 31,878 (18%) African-Americans (AA), and 12,384 (6.4%) Hispanic/Latinos (LA) with mean age of 64.5 (SD 13.1) of which 8.4% were female (similar to the proportion of females in the entire VA population). The proportion of females was higher among NAFLD cases across all ancestries.

**Table 1:** Baseline characteristics of the MVP NAFLD analytic cohort defined by the ALT-threshold definition.

		All Ancestries (n = 192,616)		European Ancestry (n = 148,354)		African Ancestry (n = 31,878)		Hispanic/Latino Ancestry (n = 12,384)	
CHARACTERISTIC	TOTAL	CASES	CONTROLS	CASES	CONTROLS	CASES	CONTROLS	CASES	CONTROLS
Participants, n	192,616	60,542	132,074	46,653	101,701	8,019	23,859	5,870	6,514
Age at enrollment, mean ± sd	64.5 ± 13.1	61.8 ± 12.1	66.2 ± 13.7	63.1 ± 11.9	64.2 ± 14.8	58.2 ± 10.9	57.0 ± 14.5	56.5 ± 13.2	59.9 ± 16.0
Female, n (%)	16,209 (8.4%)	6,107 (10.1%)	10,102 (7.6%)	4,325 (9.3%)	6,169 (6.1%)	1,230 (15.3%)	3,508 (14.7%)	552 (9.4%)	425 (6.5%)
BMI ≥30 kg/m^2^, n (%)	88,547 (50.0%)	34,676 (57.3%)	53,871 (40.8%)	26,509 (56.8%)	39,672 (39%)	47,75 (59.5%)	11,115 (46.6%)	3,392 (57.8%)	3,084 (47.3%)
Hypertension, n (%)	136,862 (71.1%)	49,423 (81.6%)	87,439 (66.2%)	38,375 (82.3%)	66,946 (65.8%)	6,921 (86.3%)	16,668 (69.9%)	4,127 (70.3%)	3,825 (58.7%)
Diabetes, n (%)	49,961 (25.9%)	21,161 (35%)	28,800 (21.8%)	16,215 (34.8%)	21,232 (20.9%)	2,907 (36.3%)	5,899 (24.7%)	2,039 (34.7%)	1,669 (25.6%)
Prediabetes, n (%)	72,505 (37.6%)	20,210 (33.4%)	52,295 (39.6%)	15,664 (33.6%)	40,043 (39.4%)	2,571 (32.1%)	9,790 (41%)	2,075 (35.3%)	2,462 (37.8%)
Dyslipidemia, n (%)	97,698 (50.7%)	40,967 (67.7%)	5,6731 (43%)	31,900 (68.4%)	44,450 (43.7%)	4,693 (58.5%)	8,578 (26.9%)	4,374 (74.5%)	3,703 (56.8%)
Metabolic Risk Factor, n (%)	179,822 (93.4%)	58,964 (97.4%)	120,858 (91.5%)	45,487 (97.5%)	93,870 (92.3%)	7,835 (97.4%)	21,282 (89.2%)	5,675 (96.7%)	5,706 (87.6%)
Alcohol misuse, n (%)	59,304 (30.8%)	18,571 (30.7%)	40,733 (30.8%)	14,659 (31.4%)	32,966 (32.4%)	2,142 (26.7%)	5,909 (18.5%)	1,770 (30.2%)	1,858 (28.5%)
Peak ALT U/L, median [IQR]	28 [21 – 42]	43 [33 – 57]	23 [18 – 29]	44 [33 – 60]	24 [19 – 31]	42 [31 – 57]	23 [18 – 31]	47 [35 – 64]	24 [19 – 31]
Cirrhosis and chronic liver disease not due to alcohol	1616 (0.8%)	1212 (2%)	404 (0.3%)	574 (1.2%)	217 (0.2%)	169 (2.1%)	115 (0.5%)	469 (8%)	72 (1.1%)

**Abbreviations:** ALT = alanine aminotransferase, AST = aspartate aminotransferase, BMI = body mass index, IQR = Interquartile range. All between and within-group comparisons were P<0.001.

The NAFLD analytic cohort had a substantial burden of cardiometabolic risk factors: 93% of participants had at least 1 metabolic risk factor, 50% had BMI ≥ 30 kg/m^2^, 71% had HTN, 26% had T2D, and 51% had DL. Approximately one third of the cohort showed evidence of alcohol misuse based on the AUDIT-C score [36] despite the exclusion of participants with alcohol use disorder diagnoses based on ICD-9-CM/10-CM. Laboratory measures consistent with advanced fibrosis were detected in 10.2% based on NAFLD fibrosis score (>0.676), 3.8% by FIB4 score (>2.670) and 9.5% based on platelet count (<150,000/μl), although fewer than 1% had diagnostic codes for cirrhosis or related complications(S2 Table in S1 File). As expected, participants with our primary NAFLD phenotype based on ALT-threshold were more likely to have concomitant metabolic risk factors compared to controls with greater obesity (57.3% vs 40.8%), HTN (81.6% vs 66.2%), T2D (35% vs 21.8%) and DL (67.7% vs 43%), but not alcohol misuse (30.7% vs 30.8%).

Similar patterns persisted across EU, AA and LA ancestries. However, alcohol misuse was more frequent among NAFLD compared to control participants with AA (26.7% vs 18.5%) and LA (30.2% vs 28.5%) but not EU (31.4% vs 32.4%) ancestries. These findings provide demographic and clinical characteristics of the NAFLD cohort in our analyses.

### Replication of published NAFLD-associated loci in MVP NAFLD analytic cohort

We next sought to replicate the NAFLD risk associations previously reported for 7 SNPs in 6 distinct genetic loci including *LYPLAL1*, *GCKR*, *HSD17B13*, *PPP1R3B*, *TM6SF2* and *PNPLA3*, using our primary and secondary NAFLD phenotype definitions (ALT-threshold and ALT-metabolic) with and without further adjustment for alcohol use and/or metabolic risk factors [3, 7, 8]. As shown in Table 2, four of the six NAFLD loci (5 of the seven tagging SNPs) were robustly associated in the trans-ethnic meta-analysis of MVP cohort across all phenotype definitions and models (all P < 1x10^-6^, S1 Fig). We observed negligible differences in effect estimates between the two NAFLD case definitions at these loci (**Methods**, Table 2), given the high overlap (97%) between ALT-threshold and ALT-metabolic as described in **Methods**. Additional adjustment for alcohol use based on AUDIT-C in **Model 3** did not affect the estimated odds ratios.

**Table 2:** Previously published NAFLD risk variants with genome-wide significant association with clinical NAFLD phenotypes across all ancestries in the Million Veteran Program NAFLD analytic cohort.

						NAFLD Phenotype: ALT-Threshold (n = 192,616 Total: 60,542 cases, 132,074 controls)						NAFLD Phenotype: ALT-Metabolic (n = 191,038 Total: 58,964 cases, 132,074 controls			
						Model 1 (Base)		Model 2 (Base + Alcohol))		Model 3 (Base+Alcohol+Metab)		Model 1 (Base)		Model 2 (Base + Alcohol)	
Previously published NAFLD risk variants															
Gene	rsID	Chr	Pos (Mb)	EA	EAF	OR (95% CI)	P	OR (95% CI)	P	OR (95% CI)	P	OR (95% CI)	P	OR (95% CI)	P
LYPLAL1	rs12137855	1	219.4	C	0.80	1.00 (0.98–1.02)	0.9	1.00 (0.98–1.02)	0.92	1.00 (0.98–1.02)	0.83	1.00 (0.98–1.02)	0.87	1.00 (0.98–1.02)	0.89
LYPLAL1*	rs3001032	1	219.7	T	0.69	1.04 (1.02–1.06)	**4.7E-07**	1.04 (1.02–1.05)	**9.9E-07**	1.04 (1.03–1.06)	**1.4E-07**	1.04 (1.02–1.05)	**1.2E-06**	1.04 (1.02–1.05)	**2.6E-06**
GCKR	rs780094	2	27.7	T	0.40	1.02 (1.00–1.03)	0.02	1.02 (1.00–1.03)	0.04	1.01 (0.99–1.03)	0.20	1.02 (1.00–1.03)	0.02	1.01 (1.00–1.03)	0.06
HSD17B13	rs72613567	4	88.2	T	0.73	1.09 (1.07–1.11)	**2.7E-22**	1.09 (1.07–1.11)	**2.2E-22**	1.10 (1.08–1.12)	**3.0E-26**	1.09 (1.07–1.11)	**8.1E-22**	1.09 (1.07–1.11)	**1.0E-21**
PPP1R3B	rs4240624	8	9.2	G	0.09	1.12 (1.09–1.14)	**1.2E-22**	1.12 (1.09–1.14)	**6.6E-22**	1.12 (1.10–1.15)	**3.3E-22**	1.12 (1.09–1.14)	**2.5E-22**	1.12 (1.09–1.14)	**2.4E-21**
TM6SF2	rs2228603	19	19.3	T	0.08	1.19 (1.15–1.22)	**4.3E-30**	1.19 (1.16–1.23)	**7.1E-31**	1.24 (1.20–1.27)	**5.0E-40**	1.19 (1.15–1.22)	**3.9E-30**	1.19 (1.16–1.23)	**1.3E-30**
TM6SF2	rs58542926	19	19.4	T	0.07	1.23 (1.19–1.26)	**3.2E-46**	1.23 (1.20–1.27)	**1.2E-47**	1.29 (1.26–1.33)	**1.1E-63**	1.23 (1.19–1.26)	**7.2E-46**	1.23 (1.20–1.27)	**2.5E-47**
PNPLA3	rs738409	22	44.3	G	0.23	1.31 (1.29–1.33)	**2.2E-210**	1.31 (1.29–1.33)	**2.4E-210**	1.35 (1.33–1.38)	**4.1E-232**	1.31 (1.29–1.33)	**3.2E-211**	1.31 (1.29–1.34)	**3.0E-210**

*Newly defined LYPLAL1 variant associated with NAFLD in the Million Veteran Program based on the regional association plot (S1 Fig).

**Abbreviations:** rsID: dbSNP identifier (build 151), Chr: chromosome,Pos (Mb): megabase position on human genome reference hg19, EA: effect allele, EAF: effect allele frequency among Europeans (Million Veteran Program), OR: odds ratio of risk in cases compared to controls per effect allele (additive model), CI: confidence interval. LYPLAL1: Lysophospholipase-like Protein 1, GCKR: glucokinase regulatory protein, HSD17B13: Hydroxysteroid 17-Beta Dehydrogenase 13, PPP1R3B: protein phosphatase 1, TM6SF2: Transmembrane 6 Superfamily Member 2, PNPLA3: patatin-like phospholipase domain-containing protein 3. **Model 1**: adjusted for age, gender, and 10 principal components (PCs). **Model 2**: covariates in Model 1 + alcohol consumption at enrollment measured by the Alcohol Use Disorder Identification Test (AUDIT-C). **Model 3**: covariates in Model 2 + metabolic risk factors (Type II diabetes/prediabetes, hypertension, dyslipidemia and BMI ≥ 30 kg/m^2^). P-values below 0.006 (adjusted for multiple comparisons) are shown in **bold font**.

We further investigated the two regions with little to no statistical association in our cohort in more detail. First, while the previously lead associated variant near the *LYPLAL1* gene (rs12137855, chr1:219,448,378) was not associated in our cohort (all P >0.80, Table 2), a regional association plot (S1 Fig) indicated a substantial association with a robust effect with a nearby SNP (rs3001032, chr1:219,727,779) (all OR = 1.04, all P < 1x10^-5^). Previous studies have shown modest associations of rs3001032 with insulin resistance (HOMA-IR, P = 1.1x10^-4^), beta-cell function (HOMA-B, P = 6.6x10^-4^), BMI (P = 1.4x10^-5^), T2D (P = 3.8x10^-14^), HDL cholesterol (P = 8.1x10^-3^), and TG (P = 0.02), in contrast to rs12137855 which was not associated with these traits (P>0.05 for all) [47–50]. Given the burden of metabolic associations, these data suggest that rs3001032 is likely to tag a true NAFLD association in this region. Second, the previously associated variant at *GCKR* (rs780094) was not strongly associated with our NAFLD phenotypes (i.e., a nominal P < 0.05 in the base model), particularly after metabolic risk factor adjustments (Table 2). We investigated whether the association between *GCKR* (rs780094) and secondary NAFLD phenotypes was sensitive to the NAFLD subtype definition depending on the respective metabolic risk factor that served as an inclusion criterion. When the NAFLD phenotype was defined by ALT-threshold + dyslipidemia (ALT2DL, S6c Table in S1 File), the association was highly significant in participants of EU ancestry (OR 1.05, P = 6.5x10^-8^) as well as in the trans-ethnic meta-analysis (OR 1.05, P = 7 x 6.5x10^-9^). These associations persisted when the models accounted for alcohol consumption (**Model 2**), but were markedly attenuated and no longer significant when the NAFLD phenotype specifically excluded dyslipidemia and only included T2D (S6d Table in S1 File), HTN (S6e Table in S1 File), or obesity (S6f Table in S1 File) in its definition.

### Comparison of established NAFLD loci across EU, AA and LA cohorts

We further explored the associations of the foregoing NAFLD risk variants between MVP participants stratified by EU, AA and LA ancestries (S5a and S5b Table in S1 File) [5–7, 9]. Similar to the trans-ethnic meta-analyses, 6 of the 8 NAFLD risk variants (including the revised *LYPLAL1* variant rs3001032) were replicated with pre-specified threshold of significance (i.e., P<0.006) among EU participants with NAFLD defined by ALT-threshold (S5a Table in S1 File) or ALT-metabolic (S5b Table in S1 File) phenotype, but not *GCKR* (rs780094). Among AA participants, only the genetic variants in *PPP1R3B* (rs4240624) and *PNPLA3* (rs738409) were replicated for both NAFLD phenotypes. Although there was a relatively modest sample of LA, in that population, there was 100% directional concordance in odds ratios for the risk alleles seen in EU participants and the *TM6SF2* (rs58542926) and *PNPLA3* (rs738409) loci were significantly associated with both NAFLD phenotypes.

### Replication of genetic loci associated with elevated ALT in MVP NAFLD cohort

Having replicated NAFLD risk-associated variants with ALT-based NAFLD phenotypes, we further examined 10 variants reported to be associated with ALT levels [3, 7, 8] including two with NAFLD (rs72613567 and rs738409), using peak ALT (ALT-max) as defined in **Methods**. As shown in Table 3, all 10 variants were strongly associated with peak levels of ALT in the entire cohort, with the strongest associations for *PNPLA3* variants. Significant associations persisted for all variants when adjusted for alcohol use in **Model 2**, while additional adjustment for metabolic risk factor in **Model 3** further increased both effect size and statistical significance for most variants except for that in *TRIB1*. In ancestry-stratified analyses (S5c Table in S1 File), all 10 variants were replicated among EU. In the AA cohort, *HSD17B13* (rs72613567) was replicated in **Model 3** as was one SNP in *ERLIN1* (rs11597086) and two in *PNPLA3* (rs2281135, rs738409). In the LA cohort, variants at each of four independent loci were also replicated.

**Table 3:** Previously published ALT-associated variants with genome-wide significance and association with maximal ALT at enrollment.

						NAFLD Phenotype: ALT-Max (n = 192,616)								
						Model 1 (Base)			Model 2 (Base + Alcohol)			Model 3 (Base+Alcohol+Metab)		
Gene	rsID	Chr	Pos	EA	EAF	Beta	SE	P	Beta	SE	P	Beta	SE	P
HSD17B13	rs6834314	4	88213808	A	0.72	0.781	0.090	**6.1E-18**	0.759	0.091	**7.7E-17**	0.805	0.090	**5.0E-19**
HSD17B13	rs72613567	4	88231392	T	0.73	0.893	0.096	**2.0E-20**	0.867	0.097	**4.6E-19**	0.920	0.096	**1.3E-21**
TRIB1	rs2954021	8	126482077	A	0.50	0.787	0.080	**6.4E-23**	0.814	0.08	**4.4E-24**	0.762	0.080	**1.4E-21**
ERLIN1	rs10883437	10	101795361	T	0.61	0.501	0.081	**5.3E-10**	0.524	0.081	**1.1E-10**	0.534	0.081	**3.4E-11**
ERLIN1	rs11597390	10	101861435	G	0.64	0.789	0.085	**1.5E-20**	0.797	0.086	**1.3E-20**	0.801	0.085	**3.8E-21**
ERLIN1	rs11597086	10	101953705	A	0.58	1.114	0.087	**9.3E-38**	1.115	0.087	**2.9E-37**	1.140	0.087	**1.9E-39**
ERLIN1	rs11591741	10	101976501	G	0.58	1.090	0.086	**5.8E-37**	1.096	0.086	**8.2E-37**	1.120	0.086	**5.6E-39**
PNPLA3	*rs738409	22	44324727	G	0.23	2.580	0.097	**1.9E-155**	2.574	0.098	**1.4E-152**	2.684	0.097	**3.9E-168**
PNPLA3	rs2281135	22	44332570	A	0.17	2.263	0.105	**4.4E-102**	2.240	0.106	**7.5E-99**	2.357	0.105	**9.5E-111**
PNPLA3	rs2143571	22	44391686	A	0.18	1.235	0.098	**1.0E-36**	1.212	0.098	**4.8E-35**	1.276	0.097	**3.7E-39**

**Abbreviations:** rsID: dbSNP identifier (build 151), Chr: chromosome, Pos: base pair position on human genome reference hg19, EA: effect allele, EAF: effect allele frequency among Europeans (Million Veteran Program), Beta: effect size estimated increase in trait per increase copy of the effect allele (additive model). SE: Standard error on Beta, HSD17B13: Hydroxysteroid 17-Beta Dehydrogenase 13, TRIB1:Tribbles Homolog 1, ERLIN1: ER Lipid Raft Associated 1, PNPLA3: patatin-like phospholipase domain-containing protein 3. **Model 1:** adjusted for age, gender, and 10 principal components (PCs), **Model 2:** covariates in Model 1 + alcohol consumption at enrollment measured by the Alcohol Use Disorder Identification Test (AUDIT-C), **Model 3:** covariates in Model 2 + Type II diabetes/prediabetes, hypertension, dyslipidemia and BMI ≥ 30 kg/m2. P-values below 0.006 (adjusted for multiple comparisons) are shown in **bold font**.

Further sensitivity analyses were performed using previously published NAFLD risk and ALT-associated genetic loci with six alternative NAFLD phenotype definitions to determine whether further optimization could be achieved (S6a–S6f Table in S1 File). Altering the ALT cutoff to >30 U/L for men and >20 U/L for women, changing ALT cutoff for the control group, specifying the additional metabolic risk factor for NAFLD inclusion (e.g. T2D versus dyslipidemia, obesity, or hypertension), and altering the number of concomitant metabolic risk factors did not appreciably alter the associations, compared to NAFLD phenotype based on ALT-threshold.

Not surprisingly, the strength of associations improved for most NAFLD risk/ALT level-associated variants with higher ALT cutoffs (S6a, S6b Table in S1 File) and by further adjusting for metabolic risk factors for most variants. The stronger associations noted between established variants and higher ALT cutoffs shows the enhanced specificity (reduction in false positive cases) of the ALT-threshold phenotype without a concomitant reduction in statistical power to detect associations.

### Clinical NAFLD phenotype performance and direct EHR review

We next performed an EHR review to assess the performance characteristics of our clinical ALT-based NAFLD phenotype definitions. The inter-rater reliability of the initial chart review was κ = 0.98. As shown in Table 4, the, ALT-threshold phenotype yielded PPV of 0.89 and 0.84 with biopsy and imaging as gold standards, respectively.

**Table 4:** Electronic health record validation of NAFLD phenotype.

**Liver Biopsy and Clinical Notes as Gold Standard (n = 178)**		
** **	**NAFLD Phenotype**	**NAFLD Phenotype**
	ABALT*	ALT-threshold**
	(n = 241)	(n = 178)
PPV	0.89	0.89
**Abdominal Imaging Studies and Clinical Notes as Gold Standard (n = 216)**		
** **	**NAFLD Phenotype**	**NAFLD Phenotype**
	ABALT*	ALT-threshold**
	(n = 216)	(n = 142)
PPV	0.71	0.84

* ALT > 30 for men and > 20 for women during at least two time points at least 6 months apart within a two-year period and no other chronic liver disease.

** ALT > 40 for men and > 30 for women during at least two time points at least 6 months apart within a two-year period and no other chronic liver disease irrespective of metabolic risk factors. Sample size is lower due to exclusion of n = 137 from cases/control due to intermediate ALT values between 20–30 units/L.

Abbreviations: ALT:alanine aminotransferase, M:male, F:female; NAFLD = non-alcoholic fatty liver disease, PPV = positive predictive value in the validation sample.

### Associations of established NAFLD risk and ALT level-associated variants with advanced fibrosis

Most NAFLD risk/ALT level-associated variants examined in our study have been associated with hepatic fibrosis progression including: *GCKR* (rs780094), *HSD17B13* (rs72613567), *TM6SF2* (rs58542926), *ERLIN1* (rs11597390, rs11597086, rs11591741) and *PNPLA3* (rs738409) [3–9, 51]. Therefore, we examined our NAFLD/ALT panel for associations with advanced fibrosis in our MVP cohort, using FIB4 score (>2.670) and NAFLD fibrosis score (≥0.676) and platelet counts at enrollment as a surrogate measure of portal hypertension. As shown in Table 5, variants in *GCKR*, *HSD17B13* and *PNPLA3* (but not *TM6SF2* and *ERLIN1*) were associated with advanced fibrosis in our overall MVP cohort, but with variable levels of significance depending on fibrosis definition. For example, significant associations were replicated for the *GCKR* variant (rs780094), both *HSD17B13* variants (rs6834314A, rs72613567T) and three *PNPLA3* variants (rs738409, rs2281135, rs2143571) using platelet count as a continuous variable. However, the use of FIB4 score replicated the associations for *HSD17B13* and *PNPLA3* variants but not *GCKR*, whereas the use of NAFLD fibrosis score replicated the associations for *PNPLA3* variants but not *HSD17B13* or GCKR.

**Table 5:** Previously published ALT level/NAFLD risk-associated variants with genome-wide significance and associations with advanced fibrosis/cirrhosis and platelet count at enrollment among patients with NAFLD (n = 60,542).

						FIB4 score >2.670 (n = 7,376 cases, 53,166 controls)		NAFLD fibrosis score ≥0.676 (n = 18,363 cases, 42,179 controls)		Platelet Count (n = 60,542)		
GENE	rsID	Chr	Pos	EA	EAF	OR (95% CI)	P	OR (95% CI)	P	BETA	SE	P
LYPLAL1	rs12137855	1	219448378	C	0.8	0.97 (0.94–1.01)	0.11	0.98 (0.95–1.01)	0.18	0.214	0.214	0.32
LYPLAL1	rs3001032	1	219727779	T	0.69	1.00 (0.97–1.03)	0.91	0.98 (0.95–1.00)	0.082	-0.298	0.178	0.095
*GCKR	rs780094	2	27741237	T	0.4	1.04 (1.01–1.07)	0.0092	1.00 (0.97–1.02)	0.8	1.605	0.179	**2.5E-19**
HSD17B13	rs6834314	4	88213808	A	0.72	1.05 (1.02–1.08)	**0.0023**	1.01 (0.98–1.04)	0.47	-1.504	0.193	**6.1E-15**
*HSD17B13	rs72613567	4	88231392	T	0.73	1.06 (1.02–1.09)	**9.7E-04**	1.02 (0.99–1.05)	0.25	-1.688	0.206	**2.2E-16**
TRIB1	rs2954021	8	126482077	A	0.5	0.99 (0.97–1.02)	0.55	0.99 (0.96–1.01)	0.25	-0.259	0.169	0.13
PPP1R3B	rs4240624	8	9184231	G	0.09	0.96 (0.92–1.01)	0.084	1.00 (0.96–1.04)	0.89	-0.376	0.266	0.16
ERLIN	rs10883437	10	101795361	T	0.61	1.02 (1.00–1.05)	0.089	1.01 (0.99–1.04)	0.4	-0.183	0.171	0.29
*ERLIN	rs11597390	10	101861435	G	0.64	1.01 (0.98–1.04)	0.68	1.00 (0.97–1.02)	0.72	0.164	0.18	0.36
*ERLIN	rs11597086	10	101953705	A	0.58	1.01 (0.98–1.03)	0.73	1.00 (0.97–1.03)	0.95	-0.068	0.184	0.71
*ERLIN	rs11591741	10	101976501	G	0.58	1.00 (0.97–1.03)	0.82	1.00 (0.97–1.02)	0.87	-0.064	0.182	0.72
TM6SF2	rs2228603	19	19329924	T	0.08	0.97 (0.92–1.03)	0.32	0.97 (0.93–1.02)	0.24	0.154	0.357	0.67
*TM6SF2	rs58542926	19	19379549	T	0.07	1.01 (0.96–1.06)	0.64	0.97 (0.93–1.01)	0.18	-0.041	0.341	0.9
*PNPLA3	rs738409	22	44324727	G	0.23	1.08 (1.05–1.12)	**2.4E-07**	1.06 (1.03–1.09)	**2.0E-05**	-2.88	0.204	**3.9E-45**
PNPLA3	rs2281135	22	44332570	A	0.17	1.08 (1.04–1.11)	**1.4E-05**	1.05 (1.02–1.09)	**0.0007**	-2.382	0.221	**5.1E-27**
PNPLA3	rs2143571	22	44391686	A	0.18	1.05 (1.02–1.09)	**0.0013**	1.04 (1.01–1.07)	**0.0042**	-1.437	0.206	**2.8E-12**

**Abbreviations:** rsID: dbSNP identifier (build 151), Chr: chromosome, Pos (Mb): megabase position on human genome reference hg19, EA: effect allele, EAF: effect allele frequency among Europeans (Million Veteran Program), OR: odds ratio, increased risk in cases compared to controls per effect allele (additive model), CI: confidence interval, Beta: effect size estimated increase in trait per increase copy of the effect allele (additive model). SE: Standard error on Beta, LYPLAL1: Lysophospholipase-like Protein 1, GCKR: glucokinase regulatory protein, HSD17B13: Hydroxysteroid 17-Beta Dehydrogenase 13, PPP1R3B: protein phosphatase 1, TM6SF2: Transmembrane 6 Superfamily Member 2, PNPLA3: patatin-like phospholipase domain-containing protein 3. All analysis adjusted for age, gender, and 10 principal components (PCs); adjustments including alcohol consumption are presented in the Supplement (S6a–S6c Table in S1 File). P-values below 0.006 (adjusted for multiple comparisons) are shown in **bold font.**

Further ancestry-stratified analyses using FIB4 (S7a Table in S1 File), NAFLD fibrosis scores (S7b Table in S1 File) and baseline platelet count (S7c Table in S1 File) showed similar results for MVP participants with EU ancestry, with significant associations for *GCKR*, *HSD17B13* and *PNPLA3* variants. Despite smaller sample sizes, analyses using baseline platelet count showed significant associations among AA participants for *GCKR* and two *PNPLA3* variants (rs738409, rs2281135) and among LA participants for *HSD17B13* variant (rs6834314) and all three *PNPLA3* variants. The use of NAFLD fibrosis score resulted in a significant association for the *TRIB1* variant (rs2954021) among LA participants (S7b Table in S1 File), although this association did not persist when using NAFLD fibrosis or FIB4 as continuous measures (S7d and S7e Table in S1 File). Overall, results were similar for models adjusted for alcohol use and metabolic risk factors and with fibrosis scores as continuous measures (S7d and S7e Table in S1 File). Thus, these results replicated the associations between advanced hepatic fibrosis and *GCKR*, *HSD17B13* and *PNPLA3* variants in our MVP cohort. Together, these data demonstrate the utility of the ALT-threshold phenotype in phenotyping NAFLD in a large EHR database.

## Discussion

In this study, we took advantage of the robust clinical EHR and genotype data from the largest and diverse NAFLD case/control cohort to date to develop a non-invasive ALT-based NAFLD phenotype that may be used in future, large-scale population-based studies. Our NAFLD phenotype is based on a few key components: chronically elevated ALT, exclusion of viral, cholestatic and other hereditary liver diseases, and exclusion of persons with alcohol-related cirrhosis.

Of the 322,259 potentially eligible MVP participants with genetic and clinical data, 19% met criteria for NAFLD as defined by the ALT-threshold phenotype. After applying exclusion criteria, of the 192,616 participants in the final NAFLD analytic cohort, 31% (n = 60,542) met criteria for NAFLD using this definition. These findings are consistent with the 18–21% NAFLD prevalence reported previously among Veterans (2003–2011) and with national estimates [23, 52, 53]. Expectedly, NAFLD participants were more likely to have metabolic risk factors than controls. In the course of developing our phenotype, we noted a high degree of overlap between the ALT-based NAFLD phenotype (ALT-threshold) and one that required a concomitant metabolic risk factor (ALT-metabolic). The very similar associations between known NAFLD risk genetic loci and these two definitions support our use of ALT-threshold as the primary NAFLD phenotype for two main reasons. The ALT-threshold definition is more parsimonious and by not including a metabolic risk factor facilitates the conduct of further genetic correlation or causal inference studies (via Mendelian randomization) to investigate the links between these individual metabolic risk factors and NAFLD (by not conditioning a phenotype on a metabolic risk factor performing causal inference studies of the influence of a risk factor and NAFLD would become problematic potentially inducing collider bias) [54]. In addition to investigating how our NAFLD phenotype associated with previously established genetic variants, we also assessed the performance characteristics of these phenotypes among Veterans with available liver biopsy and abdominal imaging data, which yielded high positive predictive values and high inter-rater reliability. The PPV noted in our study was 89% when compared to a biopsy-proven gold standard and 71% when using imaging and clinical notes as the gold standard. Results are comparable to other studies using EHR- and natural language-based processing algorithms [24, 29, 55].

The strength of our ALT-based NAFLD phenotype is that it utilizes factors routinely assessed in clinical practice and performs well even among participants with moderate alcohol consumption. Clinical models for the diagnosis of NAFLD/NASH have been validated in prospective studies, however, several require measures such as waist circumference, homeostasis model assessment of insulin resistance, or fasting insulin or fasting glucose. Several of these factors are not readily available in real-world settings [52, 53, 56].

In the course of performing genetic association studies, we made several observations regarding genetic variants in *LYPLAL1* (rs12137855) and *GCKR* (rs80094). While the previously reported association was not replicated in the *LYPLAL1* variant (rs12137855) in our cohort, a nearby variant (rs3001032) was strongly associated with our phenotype and a plethora of metabolic risk factors, suggesting that this variant tags the regional NAFLD signal. With regards to *GCKR*, our sensitivity analyses showed a highly significant association between the established *GCKR* variant and NAFLD only when dyslipidemia was included in the NAFLD definition. *GKCR* was previously found to be associated with elevated ALT, however this was in smaller, highly selected cohorts (overweight/obese Mexican women, obese children of Asian ancestry), which differed substantially from MVP enrollees [57, 58]. This was not surprising as *GCKR was previously shown to* enhance hepatic glucose uptake resulting in reduced fatty acid oxidation and increased hepatic de novo lipogenesis [59] augmenting both the risk of NAFLD and metabolic aberrations [6]. It has also been shown by others that the GCKR variant associates with dyslipidemia, while this is not the case for many other NAFLD risk-increasing genotypes such as *PNPLA3* [6, 60] and that it increases the risk of NAFLD in obese individuals [58]. In sensitivity analyses, including/excluding dyslipidemia in the NAFLD case definition might have modified the proportions of individuals carrying these risk alleles contributing to the noted differences in the reported association tests. It is also possible that the lack of apparent associations with *GCKR* may have been due to our highly specific, but less sensitive NAFLD phenotype. This would need to be confirmed in future VA studies with imaging and biopsy data.

The diversity of the MVP cohort provided an opportunity to investigate NAFLD in under-represented populations. GWAS studies for NAFLD and ALT levels have largely focused on persons of EU ancestry, with minority populations underrepresented [4]. For example, only cohorts with EU ancestry were included in the two largest studies examining hepatic steatosis (n = 7,176) and ALT (n = 45,596), whereas other studies included up to 3,124 AA and 849 LA [5–7, 9, 61]. At the same time, NAFLD prevalence has been reported to be lower among AA but higher among LA than EU in population-based studies [23, 52, 62]. Notably, our MVP cohort of 60,542 NAFLD cases included 8,019 of AA and 5,870 of LA ancestry, thereby establishing one of the largest NAFLD cohorts with multi-ethnic representation. Among AA in our MVP cohort, significant associations with NAFLD and/or ALT were detected for 6 variants, including *PNPLA3* (rs738409) and *PPP1R3B* (rs4240624), which were previously reported in 3,124 AA patients examined for hepatic steatosis by CT [61]. As for LA participants, significant associations were replicated for 9 variants including *PNPLA3 (rs738409)* further supporting the robustness of our NAFLD phenotype [61].

In MVP, we confirmed associations between several NAFLD risk variants and advanced fibrosis. In our main analyses, variants in *PNPLA3* (rs738409, rs2281135, rs2143571) exhibited strong positive associations with advanced fibrosis and negative associations with platelet count and *HSD17B13* variants (rs6834314, rs72613567) confirming the results of prior studies [3, 6, 39]. We did not find significant associations between advanced fibrosis and *TM6SF2* [18] or two additional loci *MBOAT7 and IFNL3/4* (results not shown) previously found to associate with hepatic steatosis and necroinflammation [20–22]. This may be secondary to our low sample size of patients with advanced fibrosis or the heterogeneity of fibrosis definitions across previous studies. The *GCKR* variant (rs780094) had a near-significant association with advanced fibrosis when characterized by continuous FIB4 measurement. Notably, *GCKR* was associated with a higher platelet count. This is not surprising as the variant in *GCKR* is pleiotropic and has been associated with platelet count and other human blood cell traits [63]. Interestingly, the observed prevalence of advanced fibrosis among AA was comparable to EU, differing from previous reports and suggesting possible under-recognition of NAFLD among AA in previous studies [61, 62] and/or an underestimation of how ethnic differences in pathogenic traits such as visceral adiposity underlie NAFLD susceptibility [64].

There are several limitations to this study. The requirement for abnormal ALT potentially excluded a large number of individuals with NAFLD/NASH with and without cirrhosis who did not manifest elevated liver enzymes. The primary analyses excluded those with intermediate ALT values, however, sensitivity analyses (S6 Table in S1 File) showed that genetic associations were similar when participants with intermediate values were included. Patients of Asian ancestry were not represented and women were under-represented potentially limiting generalizability. Although fibrosis was assessed non-invasively and in several different ways, the validity of these measures will need to be determined among Veterans. The sample size of Veterans with advanced fibrosis and biopsy or transient elastography data was small limiting our ability to evaluate associations with advanced fibrosis; these will be examined in future studies. We may have been limited in our ability to capture Veterans with the most severe forms of NAFLD who did not survive to MVP enrollment as well as Veterans with hepatic steatosis and normal ALT values. Despite these concerns, our accurate, genetically and clinically-validated phenotype should be amenable to large-scale scans to identify and replicate genetic causes of NAFLD and progression to complications.

## Conclusion

We leveraged the clinical and genetic data in MVP—a multi-ethnic, mega-biobank to provide a validation of a simple, non-invasive ALT-based NAFLD phenotype in a real-world, population-based, national cohort. Our phenotype may be applied to future genetic and epidemiologic studies in population-based cohorts and to aid practicing clinicians in identifying individuals at risk for NAFLD with readily available clinical data.

## Supporting information

S1 File (DOCX)

S1 Fig Regional plots of 8 independent previously published NAFLD risk loci. (PDF)
