# Supplementary material for: Validating a non-invasive, ALT-based non-alcoholic fatty liver phenotype in the million veteran program
Source: PLoS One. 2020 Aug 25;15(8):e0237430. doi: 10.1371/journal.pone.0237430 (PMC7447043; doi:10.1371/journal.pone.0237430)
Supplement: S1 File — (DOCX) [file pone.0237430.s001.docx]

**Supplemental Data. Table of Contents**

**Table S1.** List of previously published non-alcoholic fatty liver disease (NAFLD) risk, alanine aminotransferase (ALT)-associated, and chronic liver disease variants, target population, and diagnostic of liver disease

**Table S2.** Selection of final NAFLD analytic cohort for each ancestry in the Million Veteran Program

**Table S3.** International classification of disease (ICD9 and 10 codes) used to define Type II diabetes

**Table S4.** Definitions of additional NAFLD and advanced fibrosis phenotypes in MVP sensitivity analyses

**Tables S5a-c.** Associations of previously published non-alcoholic fatty liver disease (NAFLD) risk and alanine aminotransferase (ALT)-associated variants with NAFLD and ALT phenotypes in ancestry-stratified analyses among MVP participants with European ancestry and African ancestry

**Tables S6a-f.** Sensitivity analyses of previously published NAFLD risk/ALT level-associated variants and 6 additional NAFLD/ALT phenotypes among MVP participants with European ancestry, African ancestry, and meta-analysis (pooled)

**Tables S7a-e.** Sensitivity analysis of previously published NAFLD risk and ALT level-associated variants and fibrosis scores as well as baseline platelet count among MVP participants with European ancestry, African ancestry, and meta-analysis (pooled)

**Figure S1.** Regional plots of 8 independent previously published NAFLD loci

**Table S1.** List of previously published non-alcoholic fatty liver disease (NAFLD) risk, alanine aminotransferase (ALT)-associated, and chronic liver disease variants, target population, and diagnostic of liver disease

| **Nearest Gene** | **SNP ID** | **Chr** | **Phenotype** | **Diagnostic modality** | **Ancestry** | **Cohort Description** |
| --- | --- | --- | --- | --- | --- | --- |
| LYPLAL1^5^ | rs12137855 | 1 | NAFLD | CT, histology | Mostly EUR, AA, Hispanic | Population-based, enriched in heart disease |
| GCKR^5,6^ | rs780094 | 2 | NAFLD | CT, histology, lab-based | Mostly EUR, AA, Hispanic | Population-based, enriched in heart disease |
| MAPK10;HSD17B13^6^ | rs6834314 | 4 | ALT | lab-based | EUR | Population-based |
| MAPK10;HSD17B13^2^ | rs72613567 | 4 | ALT | diagnosis codes, histology | Mostly EUR, AA, Hispanic | Population-based, enriched in heart disease/NAFLD |
| TRIB1^6^ | rs2954021 | 8 | ALT | lab-based, lab-based | EUR | Population-based |
| PPP1R3B^5,6^ | rs4240624 | 8 | NAFLD | CT, histology, lab-based | Mostly EUR, AA, Hispanic | Population-based, enriched in heart disease |
| CPN1;ERLIN1;CHUK^6^ | rs10883437 | 10 | ALT | lab-based | EUR | Population-based, enriched in heart-disease based |
| CPN1;ERLIN1;CHUK^7^ | rs11597390 | 10 | ALT | lab-based | Mostly EUR, Indian Asian | Population-based |
| CPN1;ERLIN1;CHUK^7^ | rs11597086 | 10 | ALT | lab-based | Mostly EUR, Indian Asian | Population-based |
| CPN1;ERLIN1;CHUK^7^ | rs11591741 | 10 | ALT | lab-based | Mostly EUR, Indian Asian | Population-based |
| TM6SF2/NCAN^5^ | rs2228603 | 19 | NAFLD | CT, histology, lab-based | Mostly EUR, AA, Hispanic | Population-based, enriched in heart disease |
| TM6SF2^4^ | rs58542926 | 19 | NAFLD | MR spectroscopy | EUR, AA, Hispanic | Population-based, enriched in heart disease |
| PNPLA3^6^ | rs738409 | 22 | ALT, NAFLD | CT, lab-based | EUR | Population- and heart-disease based |
| PNPLA3^8^ | rs738409 | 22 | NAFLD | MR spectroscopy | EUR, AA, Hispanic | Population-based, enriched in heart disease |
| PNPLA3^5^ | rs738409 | 22 | NAFLD | CT, histology, lab-based | Mostly EUR, AA, Hispanic | Population-based and enriched in heart disease |
| PNPLA3^7^ | rs2281135 | 22 | ALT | lab-based | EUR, Indian Asian | Population-based |
| SAMM50^7^ | rs2143571 | 22 | ALT | lab-based | EUR, Indian Asian | Population-based |
| 2.Abul-Husn NS, Cheng X, Li AH, Xin Y, Schurmann C, Stevis P, et al. A Protein-Truncating HSD17B13 Variant and Protection from Chronic Liver Disease. New Engl J Med. 2018;378(12):1096-106.  4.Kozlitina J, Smagris E, Stender S, Nordestgaard BG, Zhou HH, Tybjaerg-Hansen A, et al. Exome-wide association study identifies a TM6SF2 variant that confers susceptibility to nonalcoholic fatty liver disease. Nat Genet. 2014;46(4):352-6.  5.Speliotes EK, Yerges-Armstrong LM, Wu J, Hernaez R, Kim LJ, Palmer CD, et al. Genome-wide association analysis identifies variants associated with nonalcoholic fatty liver disease that have distinct effects on metabolic traits. PLoS Genet. 2011;7(3):e1001324.  6.Chambers JC, Zhang W, Sehmi J, Li X, Wass MN, Van der Harst P, et al. Genome-wide association study identifies loci influencing concentrations of liver enzymes in plasma. Nat Genet. 2011;43(11):1131-8.  7.Yuan X, Waterworth D, Perry JR, Lim N, Song K, Chambers JC, et al. Population-based genome-wide association studies reveal six loci influencing plasma levels of liver enzymes. Am J Hum Genet. 2008;83(4):520-8.  8.Romeo S, Kozlitina J, Xing C, Pertsemlidis A, Cox D, Pennacchio LA, et al. Genetic variation in PNPLA3 confers susceptibility to nonalcoholic fatty liver disease. Nat Genet. 2008;40(12):1461-5. | | | | | | |
|  |  |  |  |  |  |  |

**Table S2.** Selection of final analytic cohort for each ancestry in the Million Veteran Program

| STUDY PARTICIPANTS | | TOTAL | EU | AA | LA |
| --- | --- | --- | --- | --- | --- |
| A. Initial MVP Participants with genetic ancestry | | **322,259** | **234,683** | **64,961** | **22,615** |
| B. Excluded participants | |  |  |  |  |
|  | Alcohol use disorder* | 51,549 | 30,879 | 16,148 | 4,522 |
|  | Other liver disease/metastatic liver cancer** | 11,468 | 8,004 | 2,612 | 852 |
|  | Viral hepatitis | 7,995 | 3,146 | 3,894 | 955 |
|  | Intermediate ALT values*** | 58,631 | 44,300 | 10,429 | 3,902 |
| C. NAFLD analytic cohort (after applying exclusions) | | **192,616** | **148,354** | **31,878** | **12,384** |
| D. NAFLD case cohort with ALT-threshold phenotype | | **60,542** | **46,653** | **8,019** | **5,870** |
|  | % Initial MVP participants with genetic ancestry | 19% | 20% | 12% | 26% |
|  | % MVP Study cohort for NAFLD analysis | 31% | 31% | 25% | 47% |
| E. NAFLD case cohort with ALT-metabolic phenotype | | **58,997** | **45,487** | **7,835** | **5,675** |
|  | % Initial MVP participants with genetic ancestry | 18% | 19% | 12% | 25% |
|  | % MVP Study cohort for NAFLD analysis | 31% | 31% | 25% | 46% |
| F. Control cohort with normal ALT | | **132,074** | **101,701** | **23,859** | **6,514** |
|  | % Initial MVP participants with genetic ancestry | 41% | 43% | 37% | 29% |
|  | % MVP Study cohort for NAFLD analysis | 69% | 69% | 75% | 53% |

* Defined as: alcoholic liver disease (K70.0), alcoholic hepatitis and/or ascites (K70.1, K70.10, K70.11), alcoholic fibrosis and sclerosis of liver (K70.2), alcoholic cirrhosis of liver and/or ascites (571.0-571.3, K70.3, K70.30, K70.31), alcoholic hepatic failure and/or coma (K70.4, K70.40, K70.41), unspecified alcoholic liver disease (K70.9), and alcohol use disorder (291, 303.0, 305.0, 303.9, F10.x)

**Defined as: hemochromatosis (275.01/E83.11, E83.119), primary biliary cholangitis (571.6/K74.3, K74.5), primary sclerosing cholangitis (576.1/K83.0), autoimmune hepatitis (571.42/K75.4), alpha-1-antitrypsin deficiency (273.4/E88.01), sarcoidosis (135/D86.9), and secondary malignant neoplasm of liver and intrahepatic bile duct (197.7/C78.7), secondary biliary cirrhosis (K74.4), Wilson’s disease (275.1/E83.00, E83.01, E83.09)

*** Defined as: ALT values between 30-40 for men, and 20-30 for women—that did not meet ALT threshold for NAFLD case or control groups.

**Table S3.** International classification of disease (ICD9 and 10 codes) used to define Type 2 diabetes mellitus

| ICD-9-CM |  |  |  |  |  |  |
| --- | --- | --- | --- | --- | --- | --- |
| **250, 250.x, 250.xx except for 250.x1 and 250.x3; 357.2; 362.0; 366.41; 648.0** | | | | | | |
| ICD-10-CM |  |  |  |  |  |  |
| **E11, E11x, E11,xx, E11xxx, O241, O241x, O241xx** | | | | |  |  |
|  |  |  |  |  |  |  |
| ICD-9-CM |  |  |  |  |  |  |
| 250, 250.x, 250.xx | Diabetes mellitus | |  |  |  |  |
| Do not include any codes with a fifth digit of 1 or 3: 250.x1, 250.x3 | | | | | | |
|  | 250.x1 | Diabetes mellitus, type 1 | | |  |  |
|  | 250.x3 | Diabetes mellitus, type 1, uncontrolled | | | | |
| 250.0, 250.0x | Diabetes mellitus without mention of complication | | | | | |
| 250.1, 250.1x | Diabetes with ketoacidosis | | |  |  |  |
| 250.2, 250.2x | Diabetes with hyperosmolarity | | | |  |  |
| 250.3, 250.3x | Diabetes with other coma | | |  |  |  |
| 250.4, 250.4x | Diabetes with renal manifestations | | | |  |  |
| 250.5, 250.5x | Diabetes with ophthalmic manifestations | | | | |  |
| 250.6, 250.6x | Diabetes with neurological manifestations | | | | |  |
| 250.7, 250.7x | Diabetes with peripheral circulatory disorders | | | | | |
| 250.8, 250.8x | Diabetes with other specified manifestations | | | | |  |
| 250.9, 250.9x | Diabetes with unspecified complication | | | | |  |
| 357.2, 357.2x | Polyneuropathy in diabetes | | | |  |  |
| 362.0, 362.0x | Diabetic retinopathy | | |  |  |  |
| 366.41 | Diabetic cataract | |  |  |  |  |
| 648.0, 648.0x | Diabetes mellitus (conditions in the mother complicating pregnancy but excluding gestational diabetes) | | | | | |
|  |  |  |  |  |  |  |
| ICD-10-CM |  |  |  |  |  |  |
| E1100 | Type 2 diab w hyprosm w/o nonket hyprgly-hypros coma (NKHHC) | | | | | |
| E1101 | Type 2 diabetes mellitus with hyperosmolarity with coma | | | | | |
| E1121 | Type 2 diabetes mellitus with diabetic nephropathy | | | | | |
| E1122 | Type 2 diabetes mellitus w diabetic chronic kidney disease | | | | | |
| E1129 | Type 2 diabetes mellitus w oth diabetic kidney complication | | | | | |
| E11311 | Type 2 diabetes w unsp diabetic retinopathy w macular edema | | | | | |
| E11319 | Type 2 diabetes w unsp diabetic rtnop w/o macular edema | | | | | |
| E11321 | Type 2 diab w mild nonprlf diabetic rtnop w macular edema | | | | | |
| E11329 | Type 2 diab w mild nonprlf diabetic rtnop w/o macular edema | | | | | |
| E11331 | Type 2 diab w moderate nonprlf diab rtnop w macular edema | | | | | |
| E11339 | Type 2 diab w moderate nonprlf diab rtnop w/o macular edema | | | | | |
| E11341 | Type 2 diab w severe nonprlf diabetic rtnop w macular edema | | | | | |
| E11349 | Type 2 diab w severe nonprlf diab rtnop w/o macular edema | | | | | |
| E11351 | Type 2 diabetes w prolif diabetic rtnop w macular edema | | | | | |
| E11359 | Type 2 diabetes w prolif diabetic rtnop w/o macular edema | | | | | |
| E1136 | Type 2 diabetes mellitus with diabetic cataract | | | | | |
| E1139 | Type 2 diabetes w oth diabetic ophthalmic complication | | | | | |
| E1140 | Type 2 diabetes mellitus with diabetic neuropathy, unsp | | | | | |
| E1141 | Type 2 diabetes mellitus with diabetic mononeuropathy | | | | | |
| E1142 | Type 2 diabetes mellitus with diabetic polyneuropathy | | | | | |
| E1143 | Type 2 diabetes w diabetic autonomic (poly)neuropathy | | | | | |
| E1144 | Type 2 diabetes mellitus with diabetic amyotrophy | | | | | |
| E1149 | Type 2 diabetes w oth diabetic neurological complication | | | | | |
| E1151 | Type 2 diabetes w diabetic peripheral angiopath w/o gangrene | | | | | |
| E1152 | Type 2 diabetes w diabetic peripheral angiopathy w gangrene | | | | | |
| E1159 | Type 2 diabetes mellitus with oth circulatory complications | | | | | |
| E11610 | Type 2 diabetes mellitus w diabetic neuropathic arthropathy | | | | | |
| E11618 | Type 2 diabetes mellitus with other diabetic arthropathy | | | | | |
| E11620 | Type 2 diabetes mellitus with diabetic dermatitis | | | | | |
| E11621 | Type 2 diabetes mellitus with foot ulcer | | | | |  |
| E11622 | Type 2 diabetes mellitus with other skin ulcer | | | | |  |
| E11628 | Type 2 diabetes mellitus with other skin complications | | | | | |
| E11630 | Type 2 diabetes mellitus with periodontal disease | | | | | |
| E11638 | Type 2 diabetes mellitus with other oral complications | | | | | |
| E11641 | Type 2 diabetes mellitus with hypoglycemia with coma | | | | | |
| E11649 | Type 2 diabetes mellitus with hypoglycemia without coma | | | | | |
| E1165 | Type 2 diabetes mellitus with hyperglycemia | | | | |  |
| E1169 | Type 2 diabetes mellitus with other specified complication | | | | | |
| E118 | Type 2 diabetes mellitus with unspecified complications | | | | | |
| E119 | Type 2 diabetes mellitus without complications | | | | | |
| O24111 | Pre-existing diabetes, type 2, in pregnancy, first trimester | | | | | |
| O24112 | Pre-exist diabetes, type 2, in pregnancy, second trimester | | | | | |
| O24113 | Pre-existing diabetes, type 2, in pregnancy, third trimester | | | | | |
| O24119 | Pre-existing diabetes, type 2, in pregnancy, unsp trimester | | | | | |
| O2412 | Pre-existing diabetes mellitus, type 2, in childbirth | | | | | |
| O2413 | Pre-existing diabetes mellitus, type 2, in the puerperium | | | | | |

**Table S4.** Definitions of additional NAFLD, ALT, and advanced fibrosis phenotypes tested in sensitivity analyses

1. **ABALT =** ALT >30 U/L for men, >20 U/L for women ≥ 6 months apart over 2 years, no viral hepatitis, and no chronic liver disease with

ALT≤30 U/L for men and ≤20 U/L for women as controls

1. **ABALT2** = ALT >40 U/L for men, >30 U/L for women ≥ 6 months apart over 2 years, no viral hepatitis, and no chronic liver disease with ALT≤40 U/L for men and ≤30 U/L for women as controls
2. **ALT2DL** = ALT >40 U/L for men, >30 U/L for women ≥ 6 months apart over 2 years, no viral hepatitis, and no chronic liver disease + dyslipidemia with ALT≤30 U/L for men and ≤20 U/L for women as controls
3. **ALT2DM =** ALT >40 U/L for men, >30 U/L for women ≥ 6 months apart over 2 years, no viral hepatitis, and no chronic liver disease + Type II diabetes or prediabetes with ALT≤30 U/L for men and ≤20 U/L for women as controls
4. **ALT2HTN =** ALT >40 U/L for men, >30 U/L for women ≥ 6 months apart over 2 years, no viral hepatitis, and no chronic liver disease + hypertension with ALT≤30 U/L for men and ≤20 U/L for women as controls
5. **ALT2OBESE =** ALT >40 U/L for men, >30 U/L for women ≥ 6 months apart over 2 years, no viral hepatitis, and no chronic liver disease + BMI ≥ 30 kg/m^2^ with ALT≤30 U/L for men and ≤20 U/L for women as controls
6. **FIB4score** = FIB4 as a continuous variable (Age [years] x AST [U/L] / (platelets [10^9/L] x sqrt (ALT))
7. **NAFLD fibrosis score** = NAFLD fibrosis score as a continuous variable (NAFFIB = -1.675+(0.037*age) +(0.094*BMI) + (1.13*(diabetes or prediabetes)) + (0.99*(AST/ALT))-(0.013*platelets) - (0.66*albumin))

**Table S5a.** Previously published NAFLD risk variants with genome-wide significance and association with NAFLD phenotype, **ALT-threshold**, in the Million Veteran Program – ancestry-stratified analysis (n=192,616)

|  |  |  |  |  |  |  | **European Ancestry**  **(n=46,653 cases, 101,701 controls)** | | | **African Ancestry**  **(n=8,019 cases, 23,859 controls)** | | | **Hispanic/Latino Ancestry**  **(n=5,870 cases, 6,514 controls)** | | |
| --- | --- | --- | --- | --- | --- | --- | --- | --- | --- | --- | --- | --- | --- | --- | --- |
| **GENE** | **rsID** | **CHR** | **POS** | **EA** | **EAF** | **Adjusted** | **OR** | **95% CI** | **P** | **OR** | **95% CI** | **P** | **OR** | **95% CI** | **P** |
| LYPLAL1 | rs12137855 | 1 | 219448378 | C | 0.8 | Model 1 | 1.01 | 0.99 - 1.03 | 0.45 | 0.95 | 0.90 - 1.00 | 0.028 | 1.01 | 0.94 - 1.08 | 0.82 |
|  |  |  |  |  |  | Model 2 | 1.01 | 0.99 - 1.03 | 0.43 | 0.95 | 0.90 - 0.99 | 0.023 | 1.01 | 0.94 - 1.09 | 0.73 |
|  |  |  |  |  |  | Model 3 | 1.01 | 0.99 - 1.03 | 0.4 | 0.95 | 0.90 - 1.00 | 0.039 | 1.05 | 0.97 - 1.12 | 0.26 |
| LYPLAL1 | rs3001032 | 1 | 219727779 | T | 0.69 | Model 1 | 1.05 | 1.04 - 1.07 | **5.5E-09** | 0.98 | 0.94 - 1.01 | 0.2 | 1.06 | 1.00 - 1.11 | 0.038 |
|  |  |  |  |  |  | Model 2 | 1.05 | 1.04 - 1.07 | **1.1E-08** | 0.98 | 0.94 - 1.01 | 0.18 | 1.06 | 1.00 - 1.11 | 0.041 |
|  |  |  |  |  |  | Model 3 | 1.06 | 1.04 - 1.08 | **2.5E-09** | 0.98 | 0.95 - 1.02 | 0.34 | 1.06 | 1.01 - 1.12 | 0.035 |
| GCKR | rs780094 | 2 | 27741237 | T | 0.4 | Model 1 | 1.02 | 1.00 - 1.04 | 0.013 | 1 | 0.96 - 1.05 | 0.86 | 1.02 | 0.96 - 1.07 | 0.59 |
|  |  |  |  |  |  | Model 2 | 1.02 | 1.00 - 1.03 | 0.034 | 1 | 0.96 - 1.05 | 0.95 | 1.01 | 0.95 - 1.06 | 0.78 |
|  |  |  |  |  |  | Model 3 | 1.01 | 0.99 - 1.03 | 0.17 | 1 | 0.95 - 1.05 | 0.95 | 1.01 | 0.95 - 1.07 | 0.79 |
| HSD17B13 | rs72613567 | 4 | 88231392 | T | 0.73 | Model 1 | 1.09 | 1.07 - 1.11 | **2.4E-20** | 1.07 | 1.00 - 1.14 | 0.048 | 1.09 | 1.02 - 1.17 | 0.023 |
|  |  |  |  |  |  | Model 2 | 1.09 | 1.07 - 1.11 | **2.2E-20** | 1.07 | 1.00 - 1.14 | 0.073 | 1.11 | 1.03 - 1.18 | 0.012 |
|  |  |  |  |  |  | Model 3 | 1.11 | 1.09 - 1.12 | **4.8E-24** | 1.08 | 1.01 - 1.16 | 0.029 | 1.1 | 1.02 - 1.19 | 0.019 |
| PPP1R3B | rs4240624 | 8 | 9184231 | G | 0.09 | Model 1 | 1.15 | 1.12 - 1.18 | **1.4E-22** | 1.09 | 1.04 - 1.13 | **0.00031** | 1.02 | 0.95 - 1.09 | 0.59 |
|  |  |  |  |  |  | Model 2 | 1.15 | 1.12 - 1.18 | **3.4E-22** | 1.08 | 1.04 - 1.13 | **0.00052** | 1.02 | 0.95 - 1.08 | 0.64 |
|  |  |  |  |  |  | Model 3 | 1.16 | 1.13 - 1.19 | **2.5E-22** | 1.09 | 1.04 - 1.13 | **0.00052** | 1.02 | 0.95 - 1.10 | 0.51 |
| TM6SF2 | rs2228603 | 19 | 19329924 | T | 0.08 | Model 1 | 1.2 | 1.17 - 1.23 | **1.5E-31** | 0.96 | 0.81 - 1.11 | 0.6 | 1.11 | 0.96 - 1.26 | 0.17 |
|  |  |  |  |  |  | Model 2 | 1.21 | 1.18 - 1.24 | **8.9E-33** | 0.95 | 0.80 - 1.10 | 0.5 | 1.1 | 0.94 - 1.25 | 0.23 |
|  |  |  |  |  |  | Model 3 | 1.25 | 1.22 - 1.28 | **1.6E-41** | 0.98 | 0.82 - 1.13 | 0.78 | 1.16 | 1.00 - 1.32 | 0.075 |
| TM6SF2 | rs58542926 | 19 | 19379549 | T | 0.07 | Model 1 | 1.25 | 1.22 - 1.28 | **7.9E-46** | 1.08 | 0.99 - 1.17 | 0.11 | 1.19 | 1.06 - 1.31 | 0.0063 |
|  |  |  |  |  |  | Model 2 | 1.25 | 1.22 - 1.28 | **4.1E-47** | 1.08 | 0.99 - 1.18 | 0.11 | 1.21 | 1.08 - 1.33 | **0.0035** |
|  |  |  |  |  |  | Model 3 | 1.32 | 1.29 - 1.35 | **1.8E-62** | 1.1 | 1.00 - 1.19 | 0.065 | 1.29 | 1.16 - 1.42 | **0.00016** |
| PNPLA3 | rs738409 | 22 | 44324727 | G | 0.23 | Model 1 | 1.31 | 1.29 - 1.33 | **6.2E-171** | 1.2 | 1.15 - 1.25 | **1.9E-13** | 1.4 | 1.34 - 1.45 | **3.2E-33** |
|  |  |  |  |  |  | Model 2 | 1.32 | 1.30 - 1.34 | **9.2E-171** | 1.21 | 1.16 - 1.25 | **1.5E-13** | 1.4 | 1.35 - 1.46 | **2.8E-33** |
|  |  |  |  |  |  | Model 3 | 1.36 | 1.34 - 1.38 | **3.8E-189** | 1.22 | 1.17 - 1.27 | **5.9E-14** | 1.47 | 1.41 - 1.52 | **8.5E-38** |

**ALT threshold:** ALT >40 U/L for men, >30 U/L for women ≥ 6 months apart over 2 years, no viral hepatitis, and no chronic liver disease with ALT≤30 U/L for men and ≤20 U/L for women as controls. **Abbreviations:** rsID: dbSNP identifier (build 151), Chr: chromosome,Pos (Mb): megabase position on human genome reference hg19, EA: effect allele, EAF: effect allele frequency among Europeans (Million Veteran Program), OR: odds ratio of risk in cases compared to controls per effect allele (additive model), CI: confidence interval. LYPLAL1: Lysophospholipase-like Protein 1, GCKR: glucokinase regulatory protein, HSD17B13: Hydroxysteroid 17-Beta Dehydrogenase 13, PPP1R3B: protein phosphatase 1, TM6SF2: Transmembrane 6 Superfamily Member 2, PNPLA3: patatin-like phospholipase domain-containing protein 3. **Model 1**: adjusted for age, gender, and 10 principal components (PCs). **Model 2**: covariates in Model 1 + alcohol consumption at enrollment measured by the Alcohol Use Disorder Identification Test (AUDIT-C). **Model 3**: covariates in Model 2 + Type II diabetes/prediabetes, hypertension, dyslipidemia and BMI ≥ 30 kg/m^2^. Control group excludes intermediate ALT values of 30-40 U/L for men and 20-30 U/L for women. P-values below 0.006 (adjusted for multiple comparisons) are shown in **bold font**.

**Table S5b.** Previously published NAFLD risk variants with genome-wide significance and association with NAFLD phenotype, **ALT-metabolic**, in the Million Veteran Program – ancestry-stratified analysis (n= 191,038)

|  |  |  |  |  |  |  | **European Ancestry**  **(n=45,487 cases, 101,701 controls)** | | | **African Ancestry**  **(n=7,835 cases, 23,859 controls)** | | | **Hispanic/Latino Ancestry**  **(n=5,675 cases, 6,514 controls)** | | |
| --- | --- | --- | --- | --- | --- | --- | --- | --- | --- | --- | --- | --- | --- | --- | --- |
| **GENE** | **rsID** | **CHR** | **POS** | **EA** | **EAF** | **Adjusted** | **OR** | **95% CI** | **P** | **OR** | **95% CI** | **P** | **OR** | **95% CI** | **P** |
| LYPLAL1 | rs12137855 | 1 | 219448378 | C | 0.8 | Model 1 | 1.01 | 0.99 - 1.03 | 0.55 | 0.95 | 0.91 - 1.00 | 0.04 | 1.01 | 0.94 - 1.09 | 0.73 |
|  | rs12137855 | 1 | 219448378 | C | 0.8 | Model 2 | 1.01 | 0.99 - 1.03 | 0.5 | 0.95 | 0.90 - 1.00 | 0.034 | 1.01 | 0.94 - 1.09 | 0.7 |
| LYPLAL1 | rs3001032 | 1 | 219727779 | T | 0.69 | Model 1 | 1.05 | 1.03 - 1.07 | **2.0E-08** | 0.98 | 0.94 - 1.01 | 0.18 | 1.06 | 1.01 - 1.11 | 0.031 |
|  | rs3001032 | 1 | 219727779 | T | 0.69 | Model 2 | 1.05 | 1.03 - 1.07 | **3.9E-08** | 0.98 | 0.94 - 1.01 | 0.17 | 1.06 | 1.00 - 1.11 | 0.038 |
| GCKR | rs780094 | 2 | 27741237 | T | 0.4 | Model 1 | 1.02 | 1.00 - 1.04 | 0.021 | 1 | 0.96 - 1.05 | 0.91 | 1.02 | 0.96 - 1.07 | 0.56 |
|  | rs780094 | 2 | 27741237 | T | 0.4 | Model 2 | 1.02 | 1.00 - 1.03 | 0.045 | 1 | 0.95 - 1.05 | 0.99 | 1.01 | 0.95 - 1.07 | 0.74 |
| HSD17B13 | rs72613567 | 4 | 88231392 | T | 0.73 | Model 1 | 1.09 | 1.07 - 1.11 | **7.4E-20** | 1.07 | 1.00 - 1.14 | 0.073 | 1.1 | 1.03 - 1.18 | 0.013 |
|  | rs72613567 | 4 | 88231392 | T | 0.73 | Model 2 | 1.09 | 1.07 - 1.11 | **9.1E-20** | 1.06 | 0.99 - 1.13 | 0.11 | 1.11 | 1.03 - 1.19 | 0.0075 |
| PPP1R3B | rs4240624 | 8 | 9184231 | G | 0.09 | Model 1 | 1.15 | 1.12 - 1.18 | **1.1E-22** | 1.08 | 1.04 - 1.13 | **0.00036** | 1.01 | 0.94 - 1.08 | 0.76 |
|  | rs4240624 | 8 | 9184231 | G | 0.09 | Model 2 | 1.15 | 1.12 - 1.18 | **4.0E-22** | 1.08 | 1.04 - 1.13 | **0.00075** | 1.01 | 0.94 - 1.08 | 0.81 |
| TM6SF2 | rs2228603 | 19 | 19329924 | T | 0.08 | Model 1 | 1.2 | 1.17 - 1.23 | **1.3E-31** | 0.96 | 0.81 - 1.11 | 0.61 | 1.11 | 0.96 - 1.26 | 0.18 |
|  | rs2228603 | 19 | 19329924 | T | 0.08 | Model 2 | 1.21 | 1.18 - 1.24 | **1.6E-32** | 0.95 | 0.80 - 1.10 | 0.51 | 1.1 | 0.94 - 1.25 | 0.24 |
| TM6SF2 | rs58542926 | 19 | 19379549 | T | 0.07 | Model 1 | 1.25 | 1.22 - 1.28 | **9.5E-46** | 1.08 | 0.98 - 1.17 | 0.12 | 1.18 | 1.06 - 1.31 | 0.009 |
|  | rs58542926 | 19 | 19379549 | T | 0.07 | Model 2 | 1.25 | 1.22 - 1.29 | **4.5E-47** | 1.08 | 0.98 - 1.17 | 0.12 | 1.2 | 1.07 - 1.32 | **0.0051** |
| PNPLA3 | rs738409 | 22 | 44324727 | G | 0.23 | Model 1 | 1.31 | 1.30 - 1.33 | **2.7E-171** | 1.21 | 1.16 - 1.26 | **3.2E-14** | 1.39 | 1.34 - 1.45 | **1.8E-32** |
|  | rs738409 | 22 | 44324727 | G | 0.23 | Model 2 | 1.32 | 1.30 - 1.34 | **3.5E-170** | 1.21 | 1.16 - 1.26 | **3.6E-14** | 1.4 | 1.35 - 1.46 | **7.9E-33** |

**ALT-metabolic:** ALT >40 U/L for men, >30 U/L for women ≥ 6 months apart over 2 years, no viral hepatitis, and no chronic liver disease with at least 1 metabolic risk factors (hypertension, diabetes, prediabetes, dyslipidemia, BMI≥30 kg/m^2^) with ALT≤30 U/L for men and ≤20 U/L for women as controls. **Abbreviations:** rsID: dbSNP identifier (build 151), Chr: chromosome,Pos (Mb): megabase position on human genome reference hg19, EA: effect allele, EAF: effect allele frequency among Europeans (Million Veteran Program), OR: odds ratio of risk in cases compared to controls per effect allele (additive model), CI: confidence interval. LYPLAL1: Lysophospholipase-like Protein 1, GCKR: glucokinase regulatory protein, HSD17B13: Hydroxysteroid 17-Beta Dehydrogenase 13, PPP1R3B: protein phosphatase 1, TM6SF2: Transmembrane 6 Superfamily Member 2, PNPLA3: patatin-like phospholipase domain-containing protein 3. **Model 1**: adjusted for age, gender, and 10 principal components (PCs). **Model 2**: covariates in Model 1 + alcohol consumption at enrollment measured by the Alcohol Use Disorder Identification Test (AUDIT-C). Control group excludes intermediate ALT values of 30-40 U/L for men and 20-30 U/L for women. P-values below 0.006 (adjusted for multiple comparisons) are shown in **bold font**.

**Table S5c.** Previously published alanine aminotransferase (ALT)-associated variants with genome-wide significance and association with peak ALT at enrollment, **ALT-max**, in the Million Veteran Program – ancestry-stratified analysis (n=192,616)

|  |  |  |  |  |  |  | **European Ancestry (n=148,354)** | | | **African Ancestry**  **(n=31,878)** | | | **Hispanic/Latino Ancestry**  **(n=12,384)** | | |
| --- | --- | --- | --- | --- | --- | --- | --- | --- | --- | --- | --- | --- | --- | --- | --- |
| **GENE** | **rsID** | **Chr** | **Pos** | **EA** | **EAF** | **Adjusted** | **Beta** | **SE** | **P** | **Beta** | **SE** | **P** | **Beta** | **SE** | **P** |
| HSD17B13 | rs6834314 | 4 | 88213808 | A | 0.72 | Model 1 | 0.833 | 0.101 | **2.1E-16** | 0.469 | 0.218 | 0.032 | 1.149 | 0.505 | 0.023 |
|  | rs6834314 | 4 | 88213808 | A | 0.72 | Model 2 | 0.813 | 0.102 | **1.9E-15** | 0.462 | 0.218 | 0.034 | 1.043 | 0.506 | 0.039 |
|  | rs6834314 | 4 | 88213808 | A | 0.72 | Model 3 | 0.856 | 0.101 | **3.3E-17** | 0.527 | 0.217 | 0.015 | 1.064 | 0.502 | 0.034 |
| HSD17B13 | rs72613567 | 4 | 88231392 | T | 0.73 | Model 1 | 0.864 | 0.102 | **2.3E-17** | 0.947 | 0.358 | 0.0081 | 1.551 | 0.53 | **0.0034** |
|  | rs72613567 | 4 | 88231392 | T | 0.73 | Model 2 | 0.839 | 0.103 | **3.5E-16** | 0.942 | 0.358 | 0.0085 | 1.448 | 0.531 | 0.0064 |
|  | rs72613567 | 4 | 88231392 | T | 0.73 | Model 3 | 0.886 | 0.102 | **3.6E-18** | 1.092 | 0.356 | **0.0021** | 1.452 | 0.527 | **0.0059** |
| TRIB1 | rs2954021 | 8 | 126482077 | A | 0.5 | Model 1 | 0.865 | 0.091 | **1.9E-21** | 0.275 | 0.187 | 0.14 | 1.513 | 0.372 | **4.7E-05** |
|  | rs2954021 | 8 | 126482077 | A | 0.5 | Model 2 | 0.897 | 0.092 | **1.5E-22** | 0.287 | 0.187 | 0.12 | 1.545 | 0.373 | **3.4E-05** |
|  | rs2954021 | 8 | 126482077 | A | 0.5 | Model 3 | 0.832 | 0.091 | **6.6E-20** | 0.29 | 0.186 | 0.12 | 1.472 | 0.37 | **7.0E-05** |
| ERLIN1 | rs10883437 | 10 | 101795361 | T | 0.61 | Model 1 | 0.532 | 0.093 | **1.0E-08** | 0.427 | 0.182 | 0.019 | 0.32 | 0.37 | 0.39 |
|  | rs10883437 | 10 | 101795361 | T | 0.61 | Model 2 | 0.564 | 0.094 | **1.8E-09** | 0.419 | 0.182 | 0.021 | 0.35 | 0.371 | 0.35 |
|  | rs10883437 | 10 | 101795361 | T | 0.61 | Model 3 | 0.569 | 0.093 | **9.3E-10** | 0.435 | 0.181 | 0.016 | 0.408 | 0.368 | 0.27 |
| ERLIN1 | rs11591741 | 10 | 101976501 | G | 0.58 | Model 1 | 1.133 | 0.092 | **7.3E-35** | 0.637 | 0.293 | 0.03 | 1.125 | 0.412 | 0.0063 |
|  | rs11591741 | 10 | 101976501 | G | 0.58 | Model 2 | 1.13 | 0.093 | **3.9E-34** | 0.713 | 0.293 | 0.015 | 1.179 | 0.413 | **0.0043** |
|  | rs11591741 | 10 | 101976501 | G | 0.58 | Model 3 | 1.147 | 0.092 | **1.1E-35** | 0.793 | 0.292 | 0.0066 | 1.229 | 0.41 | **0.0027** |
| ERLIN1 | rs11597086 | 10 | 101953705 | A | 0.58 | Model 1 | 1.13 | 0.092 | **1.1E-34** | 0.929 | 0.337 | **0.0058** | 1.069 | 0.413 | 0.0097 |
|  | rs11597086 | 10 | 101953705 | A | 0.58 | Model 2 | 1.128 | 0.093 | **5.2E-34** | 0.947 | 0.337 | **0.0049** | 1.109 | 0.414 | 0.0073 |
|  | rs11597086 | 10 | 101953705 | A | 0.58 | Model 3 | 1.146 | 0.092 | **1.4E-35** | 1.059 | 0.335 | **0.0016** | 1.145 | 0.411 | **0.0053** |
| ERLIN1 | rs11597390 | 10 | 101861435 | G | 0.64 | Model 1 | 0.889 | 0.094 | **4.7E-21** | 0.301 | 0.227 | 0.18 | 0.544 | 0.382 | 0.15 |
|  | rs11597390 | 10 | 101861435 | G | 0.64 | Model 2 | 0.891 | 0.095 | **7.8E-21** | 0.322 | 0.227 | 0.16 | 0.616 | 0.382 | 0.11 |
|  | rs11597390 | 10 | 101861435 | G | 0.64 | Model 3 | 0.887 | 0.094 | **6.1E-21** | 0.36 | 0.226 | 0.11 | 0.672 | 0.38 | 0.077 |
| PNPLA3 | rs2143571 | 22 | 44391686 | A | 0.18 | Model 1 | 1.587 | 0.118 | **5.1E-41** | 0.113 | 0.191 | 0.55 | 2.173 | 0.405 | **7.9E-08** |
|  | rs2143571 | 22 | 44391686 | A | 0.18 | Model 2 | 1.557 | 0.119 | **7.6E-39** | 0.126 | 0.19 | 0.51 | 2.16 | 0.406 | **1.0E-07** |
|  | rs2143571 | 22 | 44391686 | A | 0.18 | Model 3 | 1.629 | 0.118 | **5.2E-43** | 0.18 | 0.19 | 0.34 | 2.135 | 0.402 | **1.1E-07** |
| PNPLA3 | rs2281135 | 22 | 44332570 | A | 0.17 | Model 1 | 2.273 | 0.122 | **6.5E-78** | 1.477 | 0.253 | **5.4E-09** | 4.009 | 0.389 | **7.3E-25** |
|  | rs2281135 | 22 | 44332570 | A | 0.17 | Model 2 | 2.245 | 0.123 | **9.2E-75** | 1.485 | 0.253 | **4.3E-09** | 3.987 | 0.389 | **1.6E-24** |
|  | rs2281135 | 22 | 44332570 | A | 0.17 | Model 3 | 2.364 | 0.122 | **4.9E-84** | 1.59 | 0.252 | **2.8E-10** | 4.087 | 0.387 | **5.4E-26** |
| PNPLA3 | rs738409 | 22 | 44324727 | G | 0.23 | Model 1 | 2.577 | 0.109 | **3.4E-123** | 1.697 | 0.259 | **6.1E-11** | 4.476 | 0.376 | **1.9E-32** |
|  | rs738409 | 22 | 44324727 | G | 0.23 | Model 2 | 2.569 | 0.11 | **2.8E-120** | 1.713 | 0.259 | **4.0E-11** | 4.457 | 0.377 | **4.1E-32** |
|  | rs738409 | 22 | 44324727 | G | 0.23 | Model 3 | 2.681 | 0.109 | **5.9E-133** | 1.809 | 0.258 | **2.4E-12** | 4.55 | 0.374 | **7.6E-34** |

**ALT max:** Maximum ALT value at enrollment. **Abbreviations:** rsID: dbSNP identifier (build 151), Chr: chromosome, Pos: basepair position on human genome reference hg19, EA: effect allele, EAF: effect allele frequency among Europeans (Million Veteran Program), Beta: effect size estimated increase in trait per increase copy of the effect allele (additive model). SE: Standard error on Beta, HSD17B13: Hydroxysteroid 17-Beta Dehydrogenase 13, TRIB1:Tribbles Homolog 1, ERLIN1: ER Lipid Raft Associated 1, PNPLA3: patatin-like phospholipase domain-containing protein 3. **Model 1:** adjusted for age, gender, and 10 principal components (PCs), **Model 2:** covariates in Model 1 + alcohol consumption at enrollment measured by the Alcohol Use Disorder Identification Test (AUDIT-C), **Model 3:** covariates in Model 2 + Type II diabetes/prediabetes, hypertension, dyslipidemia and BMI ≥ 30 kg/m2. P-values below 0.006 (adjusted for multiple comparisons) are shown in **bold font**.

**Table S6a.** Previously published NAFLD risk/ALT level-associated variants with genome-wide significance and association with NAFLD phenotype, **ABALT**

|  |  |  |  |  |  |  | **European Ancestry**  **(n = 90,953 cases, 101,701 controls)** | | | **African Ancestry**  **(n=18,448 cases, 23,859 controls)** | | | **Hispanic/Latino Ancestry**  **(n=9,772 cases, 6,514 controls)** | | | **Trans-ethnic Meta-Analysis**  **(n=119,173 cases, 132,074 controls)** | | |
| --- | --- | --- | --- | --- | --- | --- | --- | --- | --- | --- | --- | --- | --- | --- | --- | --- | --- | --- |
| **Gene** | **rsID** | **Chr** | **Pos** | **EA** | **EAF** | **ADJUSTED** | **OR** | **95% CI** | **P** | **OR** | **95% CI** | **P** | **OR** | **95% CI** | **P** | **OR** | **95% CI** | **P** |
| LYPLAL1 | rs12137855 | 1 | 219448378 | C | 0.8 | Model 1 | 1.00 | 0.98 - 1.02 | 0.988 | 0.97 | 0.93 - 1.01 | 0.141 | 1.05 | 0.98 - 1.12 | 0.137 | 1.00 | 0.98 - 1.01 | 0.833 |
|  | rs12137855 | 1 | 219448378 | C | 0.8 | Model 2 | 1.00 | 0.98 - 1.02 | 0.992 | 0.97 | 0.93 - 1.01 | 0.110 | 1.06 | 0.99 - 1.13 | 0.109 | 1.00 | 0.98 - 1.01 | 0.796 |
|  | rs12137855 | 1 | 219448378 | C | 0.8 | Model 3 | 1.00 | 0.98 - 1.02 | 0.932 | 0.97 | 0.93 - 1.01 | 0.132 | 1.08 | 1.01 - 1.16 | 0.032 | 1.00 | 0.98 - 1.02 | 0.868 |
| LYPLAL1 | rs3001032 | 1 | 219727779 | T | 0.69 | Model 1 | 1.04 | 1.02 - 1.05 | **1.8E-06** | 0.98 | 0.95 - 1.01 | 0.116 | 1.05 | 1.00 - 1.10 | 0.060 | 1.03 | 1.01 - 1.04 | **8.9E-05** |
|  | rs3001032 | 1 | 219727779 | T | 0.69 | Model 2 | 1.04 | 1.02 - 1.06 | **9.2E-07** | 0.98 | 0.95 - 1.01 | 0.146 | 1.05 | 1.00 - 1.10 | 0.063 | 1.03 | 1.01 - 1.04 | **4.8E-05** |
|  | rs3001032 | 1 | 219727779 | T | 0.69 | Model 3 | 1.04 | 1.03 - 1.06 | **2.4E-07** | 0.98 | 0.95 - 1.01 | 0.211 | 1.05 | 1.00 - 1.10 | 0.056 | 1.03 | 1.02 - 1.05 | **1.2E-05** |
| GCKR | rs780094 | 2 | 27741237 | T | 0.4 | Model 1 | 1.02 | 1.00 - 1.03 | 0.047 | 1.00 | 0.96 - 1.04 | 0.929 | 1.01 | 0.96 - 1.06 | 0.803 | 1.01 | 1.00 - 1.03 | 0.058 |
|  | rs780094 | 2 | 27741237 | T | 0.4 | Model 2 | 1.01 | 1.00 - 1.03 | 0.105 | 1.00 | 0.96 - 1.04 | 0.941 | 0.10 | 0.95 - 1.05 | 0.951 | 1.01 | 1.00 - 1.02 | 0.153 |
|  | rs780094 | 2 | 27741237 | T | 0.4 | Model 3 | 1.01 | 0.99 - 1.02 | 0.276 | 0.99 | 0.95 - 1.04 | 0.781 | 0.99 | 0.94 - 1.05 | 0.806 | 1.01 | 0.99 - 1.02 | 0.406 |
| HSD17B13 | rs6834314 | 4 | 88213808 | A | 0.72 | Model 1 | 1.05 | 1.03 - 1.06 | **3.6E-08** | 1.01 | 0.97 - 1.04 | 0.752 | 1.02 | 0.95 - 1.09 | 0.602 | 1.04 | 1.02 - 1.05 | **2.3E-07** |
|  | rs6834314 | 4 | 88213808 | A | 0.72 | Model 2 | 1.05 | 1.03 - 1.06 | **4.4E-08** | 1.00 | 0.97 - 1.04 | 0.873 | 1.02 | 0.95 - 1.09 | 0.562 | 1.04 | 1.02 - 1.05 | **3.8E-07** |
|  | rs6834314 | 4 | 88213808 | A | 0.72 | Model 3 | 1.06 | 1.04 - 1.07 | **1.3E-10** | 1.01 | 0.97 - 1.05 | 0.546 | 1.02 | 0.95 - 1.09 | 0.597 | 1.05 | 1.03 - 1.06 | **1.1E-09** |
| HSD17B13 | rs72613567 | 4 | 88231392 | T | 0.73 | Model 1 | 1.05 | 1.03 - 1.06 | **1.2E-08** | 1.01 | 0.95 - 1.07 | 0.881 | 1.04 | 0.97 - 1.11 | 0.323 | 1.04 | 1.03 - 1.06 | **1.8E-08** |
|  | rs72613567 | 4 | 88231392 | T | 0.73 | Model 2 | 1.05 | 1.03 - 1.06 | **1.6E-08** | 1.00 | 0.94 - 1.06 | 0.971 | 1.04 | 0.97 - 1.11 | 0.323 | 1.04 | 1.03 - 1.06 | **3.3E-08** |
|  | rs72613567 | 4 | 88231392 | T | 0.73 | Model 3 | 1.06 | 1.04 - 1.07 | **8.2E-11** | 1.01 | 0.95 - 1.08 | 0.649 | 1.04 | 0.97 - 1.11 | 0.309 | 1.05 | 1.04 - 1.07 | **1.1E-10** |
| PPP1R3B | rs4240624 | 8 | 9184231 | G | 0.09 | Model 1 | 1.09 | 1.07 - 1.12 | **9.0E-13** | 1.06 | 1.02 - 1.10 | **0.002** | 1.02 | 0.96 - 1.08 | 0.575 | 1.08 | 1.06 - 1.10 | **7.2E-14** |
|  | rs4240624 | 8 | 9184231 | G | 0.09 | Model 2 | 1.10 | 1.07 - 1.12 | **5.6E-13** | 1.06 | 1.02 - 1.10 | **0.003** | 1.01 | 0.95 - 1.08 | 0.689 | 1.08 | 1.06 - 1.10 | **1.4E-13** |
|  | rs4240624 | 8 | 9184231 | G | 0.09 | Model 3 | 1.11 | 1.08 - 1.13 | **5.1E-14** | 1.07 | 1.03 - 1.11 | **0.001** | 1.02 | 0.96 - 1.09 | 0.518 | 1.09 | 1.06 - 1.11 | **3.9E-15** |
| TRIB1 | rs2954021 | 8 | 126482077 | A | 0.5 | Model 1 | 1.09 | 1.07 - 1.10 | **2.6E-32** | 1.01 | 0.98 - 1.04 | 0.708 | 1.07 | 1.02 - 1.12 | 0.009 | 1.07 | 1.06 - 1.09 | **2.9E-29** |
|  | rs2954021 | 8 | 126482077 | A | 0.5 | Model 2 | 1.09 | 1.07 - 1.10 | **1.5E-31** | 1.01 | 0.98 - 1.04 | 0.652 | 1.06 | 1.01 - 1.12 | 0.011 | 1.07 | 1.06 - 1.09 | **1.3E-28** |
|  | rs2954021 | 8 | 126482077 | A | 0.5 | Model 3 | 1.08 | 1.06 - 1.09 | **1.4E-23** | 1.01 | 0.98 - 1.04 | 0.471 | 1.05 | 1.00 - 1.10 | 0.062 | 1.07 | 1.05 - 1.08 | **1.3E-21** |
| ERLIN1 | rs10883437 | 10 | 101795361 | T | 0.61 | Model 1 | 1.06 | 1.05 - 1.08 | **8.5E-16** | 1.02 | 0.99 - 1.05 | 0.231 | 1.06 | 1.01 - 1.11 | 0.016 | 1.05 | 1.04 - 1.07 | **4.0E-16** |
|  | rs10883437 | 10 | 101795361 | T | 0.61 | Model 2 | 1.06 | 1.05 - 1.08 | **1.0E-15** | 1.02 | 0.99 - 1.05 | 0.267 | 1.06 | 1.01 - 1.11 | 0.025 | 1.05 | 1.04 - 1.07 | **9.8E-16** |
|  | rs10883437 | 10 | 101795361 | T | 0.61 | Model 3 | 1.07 | 1.05 - 1.08 | **1.9E-17** | 1.02 | 0.99 - 1.05 | 0.252 | 1.06 | 1.01 - 1.11 | 0.022 | 1.06 | 1.04 - 1.07 | **2.6E-17** |
| ERLIN1 | rs11597390 | 10 | 101861435 | G | 0.64 | Model 1 | 1.09 | 1.08 - 1.11 | **1.2E-33** | 1.05 | 1.01 - 1.09 | 0.007 | 1.05 | 0.99 - 1.10 | 0.081 | 1.09 | 1.07 - 1.10 | **1.2E-34** |
|  | rs11597390 | 10 | 101861435 | G | 0.64 | Model 2 | 1.10 | 1.08 - 1.11 | **4.1E-33** | 1.05 | 1.01 - 1.09 | 0.010 | 1.04 | 0.99 - 1.09 | 0.103 | 1.09 | 1.07 - 1.10 | **1.2E-33** |
|  | rs11597390 | 10 | 101861435 | G | 0.64 | Model 3 | 1.10 | 1.09 - 1.12 | **2.9E-35** | 1.05 | 1.01 - 1.09 | 0.011 | 1.05 | 0.99 - 1.10 | 0.097 | 1.09 | 1.08 - 1.11 | **1.4E-35** |
| ERLIN1 | rs11597086 | 10 | 101953705 | A | 0.58 | Model 1 | 1.12 | 1.10 - 1.13 | **2.4E-51** | 1.11 | 1.05 - 1.17 | **3.5E-04** | 1.08 | 1.02 - 1.14 | **0.004** | 1.11 | 1.10 - 1.13 | **1.3E-55** |
|  | rs11597086 | 10 | 101953705 | A | 0.58 | Model 2 | 1.12 | 1.10 - 1.13 | **1.4E-50** | 1.10 | 1.04 - 1.17 | **5.4E-04** | 1.08 | 1.02 - 1.13 | 0.008 | 1.11 | 1.10 - 1.13 | **2.4E-54** |
|  | rs11597086 | 10 | 101953705 | A | 0.58 | Model 3 | 1.13 | 1.11 - 1.14 | **1.1E-55** | 1.12 | 1.06 - 1.19 | **1.1E-04** | 1.08 | 1.03 - 1.15 | **0.004** | 1.12 | 1.11 - 1.14 | **2.4E-60** |
| ERLIN1 | rs11591741 | 10 | 101976501 | G | 0.58 | Model 1 | 1.12 | 1.10 - 1.13 | **2.7E-51** | 1.09 | 1.04 - 1.14 | **6.0E-04** | 1.08 | 1.03 - 1.14 | **0.003** | 1.11 | 1.10 - 1.13 | **2.1E-55** |
|  | rs11591741 | 10 | 101976501 | G | 0.58 | Model 2 | 1.12 | 1.10 - 1.13 | **1.6E-50** | 1.09 | 1.04 - 1.14 | **8.0E-04** | 1.08 | 1.02 - 1.14 | **0.005** | 1.11 | 1.10 - 1.13 | **3.3E-54** |
|  | rs11591741 | 10 | 101976501 | G | 0.58 | Model 3 | 1.13 | 1.11 - 1.14 | **1.2E-55** | 1.10 | 1.04 - 1.15 | **2.6E-04** | 1.09 | 1.03 - 1.15 | **0.003** | 1.12 | 1.11 - 1.14 | **4.6E-60** |
| TM6SF2 | rs2228603 | 19 | 19329924 | T | 0.08 | Model 1 | 1.11 | 1.08 - 1.14 | **1.5E-14** | 1.01 | 0.89 - 1.14 | 0.921 | 1.06 | 0.92 - 1.21 | 0.447 | 1.11 | 1.08 - 1.13 | **4.4E-14** |
|  | rs2228603 | 19 | 19329924 | T | 0.08 | Model 2 | 1.12 | 1.09 - 1.15 | **2.7E-15** | 0.99 | 0.88 - 1.13 | 0.931 | 1.05 | 0.91 - 1.20 | 0.536 | 1.11 | 1.08 - 1.14 | **1.5E-14** |
|  | rs2228603 | 19 | 19329924 | T | 0.08 | Model 3 | 1.15 | 1.11 - 1.18 | **8.3E-21** | 1.02 | 0.89 - 1.16 | 0.826 | 1.10 | 0.95 - 1.27 | 0.208 | 1.14 | 1.11 - 1.17 | **2.0E-20** |
| TM6SF2 | rs58542926 | 19 | 19379549 | T | 0.07 | Model 1 | 1.14 | 1.11 - 1.17 | **1.5E-20** | 1.07 | 0.98 - 1.16 | 0.117 | 1.10 | 0.98 - 1.23 | 0.092 | 1.13 | 1.10 - 1.16 | **3.2E-21** |
|  | rs58542926 | 19 | 19379549 | T | 0.07 | Model 2 | 1.14 | 1.11 - 1.17 | **3.6E-21** | 1.07 | 0.98 - 1.16 | 0.116 | 1.12 | 1.00 - 1.25 | 0.053 | 1.13 | 1.10 - 1.16 | **4.9E-22** |
|  | rs58542926 | 19 | 19379549 | T | 0.07 | Model 3 | 1.18 | 1.15 - 1.21 | **3.6E-30** | 1.08 | 1.00 - 1.18 | 0.060 | 1.19 | 1.05 - 1.33 | **0.005** | 1.17 | 1.14 - 1.20 | **6.2E-32** |
| PNPLA3 | rs738409 | 22 | 44324727 | G | 0.23 | Model 1 | 1.17 | 1.15 - 1.19 | **2.7E-72** | 1.11 | 1.06 - 1.15 | **4.1E-06** | 1.21 | 1.15 - 1.27 | **1.7E-14** | 1.16 | 1.15 - 1.18 | **6.2E-88** |
|  | rs738409 | 22 | 44324727 | G | 0.23 | Model 2 | 1.17 | 1.15 - 1.19 | **6.4E-73** | 1.10 | 1.06 - 1.15 | **6.4E-06** | 1.21 | 1.16 - 1.28 | **1.3E-14** | 1.17 | 1.15 - 1.18 | **2.5E-88** |
|  | rs738409 | 22 | 44324727 | G | 0.23 | Model 3 | 1.20 | 1.17 - 1.22 | **2.1E-85** | 1.11 | 1.07 - 1.16 | **1.8E-06** | 1.25 | 1.19 - 1.32 | **1.1E-17** | 1.19 | 1.17 - 1.21 | **1.3E-103** |
| PNPLA3 | rs2281135 | 22 | 44332570 | A | 0.17 | Model 1 | 1.14 | 1.12 - 1.17 | **1.0E-44** | 1.10 | 1.05 - 1.14 | **1.7E-05** | 1.17 | 1.10 - 1.22 | **1.7E-08** | 1.14 | 1.12 - 1.16 | **7.6E-55** |
|  | rs2281135 | 22 | 44332570 | A | 0.17 | Model 2 | 1.15 | 1.12 - 1.17 | **4.1E-44** | 1.10 | 1.05 - 1.14 | **3.7E-05** | 1.16 | 1.10 - 1.22 | **8.3E-09** | 1.14 | 1.12 - 1.16 | **4.8E-54** |
|  | rs2281135 | 22 | 44332570 | A | 0.17 | Model 3 | 1.17 | 1.15 - 1.19 | **1.1E-53** | 1.10 | 1.05 - 1.15 | **1.3E-05** | 1.20 | 1.13 - 1.26 | **4.2E-11** | 1.16 | 1.14 - 1.18 | **9.9E-66** |
| PNPLA3 | rs2143571 | 22 | 44391686 | A | 0.18 | Model 1 | 1.10 | 1.08 - 1.12 | **3.9E-25** | 1.01 | 0.98 - 1.04 | 0.595 | 1.10 | 1.05 - 1.16 | **2.4E-04** | 1.08 | 1.06 - 1.10 | **4.6E-23** |
|  | rs2143571 | 22 | 44391686 | A | 0.18 | Model 2 | 1.10 | 1.08 - 1.12 | **6.2E-24** | 1.01 | 0.97 - 1.04 | 0.763 | 1.11 | 1.05 - 1.17 | **1.7E-04** | 1.08 | 1.06 - 1.09 | **1.1E-21** |
|  | rs2143571 | 22 | 44391686 | A | 0.18 | Model 3 | 1.12 | 1.10 - 1.14 | **9.6E-30** | 1.00 | 0.97 - 1.04 | 0.799 | 1.12 | 1.06 - 1.18 | **8.1E-05** | 1.09 | 1.07 - 1.11 | **3.0E-26** |

**ABALT:** ALT >30 U/L for men, >20 U/L for women ≥ 6 months apart over 2 years, no viral hepatitis, and no chronic liver disease with ALT≤30 U/L for men and ≤20 U/L for women as controls. **Abbreviations:** rsID: dbSNP identifier (build 151), Chr: chromosome, Pos: basepair position on human genome reference hg19, EA: effect allele, EAF: effect allele frequency among Europeans (Million Veteran Program), Beta: effect size estimated increase in trait per increase copy of the effect allele (additive model). SE: Standard error on Beta. **Model 1:** adjusted for age, gender, and 10 principal components (PCs), **Model 2:** covariates in Model 1 + alcohol consumption at enrollment measured by the Alcohol Use Disorder Identification Test (AUDIT-C), **Model 3:** covariates in Model 2 + Type II diabetes/prediabetes, hypertension, dyslipidemia and BMI ≥ 30 kg/m2. P-values below 0.006 (adjusted for multiple comparisons) are shown in **bold font**.

**Table S6b.** Previously published NAFLD risk /ALT level-associated variants with genome-wide significance and association with NAFLD phenotype, **ABALT2**

|  |  |  |  |  |  |  | **European Ancestry**  **(n=46,653 cases, 146,001 controls)** | | | **African Ancestry**  **(n=8,019 cases, 34,288 controls)** | | | **Hispanic/Latino Ancestry**  **(n=5,870 cases, 10,416 controls)** | | | **Transethnic Meta-Analysis (n=60,542 cases, 190,705 controls)** | | |
| --- | --- | --- | --- | --- | --- | --- | --- | --- | --- | --- | --- | --- | --- | --- | --- | --- | --- | --- |
| **Gene** | **rsID** | **Chr** | **Pos** | **EA** | **EAF** | **ADJUSTED** | **OR** | **95% CI** | **P** | **OR** | **95% CI** | **P** | **OR** | **95% CI** | **P** | **OR** | **95% CI** | **P** |
| LYPLAL1 | rs12137855 | 1 | 219448378 | C | 0.8 | Model 1 | 1.01 | 0.99 - 1.03 | 0.21 | 0.95 | 0.91 - 1.00 | 0.037 | 0.98 | 0.92 - 1.05 | 0.64 | 1 | 0.99 - 1.02 | 0.84 |
|  | rs12137855 | 1 | 219448378 | C | 0.8 | Model 2 | 1.01 | 0.99 - 1.03 | 0.18 | 0.95 | 0.91 - 1.00 | 0.038 | 0.99 | 0.92 - 1.06 | 0.71 | 1 | 0.99 - 1.02 | 0.76 |
|  | rs12137855 | 1 | 219448378 | C | 0.8 | Model 3 | 1.01 | 0.99 - 1.03 | 0.19 | 0.96 | 0.92 - 1.00 | 0.058 | 1 | 0.93 - 1.08 | 0.95 | 1 | 0.99 - 1.02 | 0.66 |
| LYPLAL1 | rs3001032 | 1 | 219727779 | T | 0.69 | Model 1 | 1.04 | 1.03 - 1.06 | **4.1E-07** | 0.98 | 0.95 - 1.01 | 0.25 | 1.05 | 1.00 - 1.10 | 0.074 | 1.03 | 1.02 - 1.05 | **1.2E-05** |
|  | rs3001032 | 1 | 219727779 | T | 0.69 | Model 2 | 1.04 | 1.02 - 1.06 | **1.2E-06** | 0.98 | 0.95 - 1.01 | 0.23 | 1.04 | 0.99 - 1.10 | 0.08 | 1.03 | 1.02 - 1.05 | **3.4E-05** |
|  | rs3001032 | 1 | 219727779 | T | 0.69 | Model 3 | 1.05 | 1.03 - 1.06 | **4.0E-07** | 0.98 | 0.95 - 1.02 | 0.33 | 1.05 | 1.00 - 1.10 | 0.063 | 1.03 | 1.02 - 1.05 | **8.8E-06** |
| GCKR | rs780094 | 2 | 27741237 | T | 0.4 | Model 1 | 1.02 | 1.00 - 1.03 | 0.015 | 1 | 0.96 - 1.05 | 0.85 | 1.02 | 0.97 - 1.07 | 0.49 | 1.02 | 1.00 - 1.03 | 0.014 |
|  | rs780094 | 2 | 27741237 | T | 0.4 | Model 2 | 1.02 | 1.00 - 1.03 | 0.035 | 1 | 0.96 - 1.05 | 0.89 | 1.01 | 0.96 - 1.07 | 0.62 | 1.02 | 1.00 - 1.03 | 0.036 |
|  | rs780094 | 2 | 27741237 | T | 0.4 | Model 3 | 1.01 | 1.00 - 1.03 | 0.091 | 1 | 0.96 - 1.04 | 0.95 | 1.01 | 0.96 - 1.06 | 0.75 | 1.01 | 1.00 - 1.03 | 0.11 |
| HSD17B13 | rs6834314 | 4 | 88213808 | A | 0.72 | Model 1 | 1.09 | 1.07 - 1.10 | **6.0E-22** | 1.03 | 0.99 - 1.08 | 0.092 | 1.11 | 1.04 - 1.18 | 0.0028 | 1.08 | 1.06 - 1.10 | **2.3E-23** |
|  | rs6834314 | 4 | 88213808 | A | 0.72 | Model 2 | 1.09 | 1.07 - 1.11 | **4.0E-22** | 1.03 | 0.99 - 1.07 | 0.12 | 1.12 | 1.04 - 1.19 | **0.0015** | 1.08 | 1.06 - 1.10 | **1.8E-23** |
|  | rs6834314 | 4 | 88213808 | A | 0.72 | Model 3 | 1.1 | 1.08 - 1.12 | **2.1E-26** | 1.04 | 1.00 - 1.08 | 0.056 | 1.12 | 1.05 - 1.20 | **0.0014** | 1.09 | 1.08 - 1.11 | **5.4E-28** |
| HSD17B13 | rs72613567 | 4 | 88231392 | T | 0.73 | Model 1 | 1.09 | 1.07 - 1.11 | **4.9E-23** | 1.09 | 1.02 - 1.17 | 0.0078 | 1.13 | 1.05 - 1.21 | **0.00092** | 1.09 | 1.07 - 1.11 | **8.2E-27** |
|  | rs72613567 | 4 | 88231392 | T | 0.73 | Model 2 | 1.09 | 1.07 - 1.11 | **6.7E-23** | 1.09 | 1.02 - 1.17 | 0.011 | 1.14 | 1.06 - 1.22 | **0.00041** | 1.09 | 1.07 - 1.11 | **8.9E-27** |
|  | rs72613567 | 4 | 88231392 | T | 0.73 | Model 3 | 1.1 | 1.08 - 1.12 | **6.0E-27** | 1.11 | 1.04 - 1.19 | **0.0031** | 1.15 | 1.06 - 1.23 | **0.00028** | 1.1 | 1.09 - 1.12 | **1.5E-31** |
| PPP1R3B | rs4240624 | 8 | 9184231 | G | 0.09 | Model 1 | 1.14 | 1.11 - 1.16 | **8.8E-22** | 1.07 | 1.03 - 1.12 | **0.00079** | 1.02 | 0.96 - 1.08 | 0.54 | 1.11 | 1.08 - 1.13 | **1.3E-21** |
|  | rs4240624 | 8 | 9184231 | G | 0.09 | Model 2 | 1.13 | 1.10 - 1.16 | **5.5E-21** | 1.07 | 1.03 - 1.12 | **0.0012** | 1.02 | 0.96 - 1.08 | 0.56 | 1.1 | 1.08 - 1.13 | **1.2E-20** |
|  | rs4240624 | 8 | 9184231 | G | 0.09 | Model 3 | 1.14 | 1.11 - 1.17 | **1.1E-21** | 1.08 | 1.03 - 1.12 | **0.0008** | 1.03 | 0.96 - 1.10 | 0.43 | 1.11 | 1.09 - 1.14 | **1.1E-21** |
| TRIB1 | rs2954021 | 8 | 126482077 | A | 0.5 | Model 1 | 1.12 | 1.11 - 1.14 | **7.3E-52** | 1.03 | 0.99 - 1.06 | 0.096 | 1.11 | 1.05 - 1.16 | **4.8E-05** | 1.11 | 1.09 - 1.12 | **1.8E-51** |
|  | rs2954021 | 8 | 126482077 | A | 0.5 | Model 2 | 1.12 | 1.11 - 1.14 | **6.9E-52** | 1.03 | 1.00 - 1.07 | 0.087 | 1.11 | 1.05 - 1.16 | **6.9E-05** | 1.11 | 1.09 - 1.12 | **2.2E-51** |
|  | rs2954021 | 8 | 126482077 | A | 0.5 | Model 3 | 1.12 | 1.10 - 1.14 | **2.6E-43** | 1.04 | 1.00 - 1.07 | 0.053 | 1.1 | 1.04 - 1.15 | **0.00043** | 1.1 | 1.09 - 1.12 | **1.8E-43** |
| ERLIN1 | rs10883437 | 10 | 101795361 | T | 0.61 | Model 1 | 1.07 | 1.05 - 1.09 | **2.3E-17** | 1.03 | 1.00 - 1.06 | 0.081 | 1.04 | 0.99 - 1.09 | 0.11 | 1.06 | 1.05 - 1.07 | **1.3E-17** |
|  | rs10883437 | 10 | 101795361 | T | 0.61 | Model 2 | 1.07 | 1.05 - 1.09 | **4.6E-17** | 1.03 | 1.00 - 1.06 | 0.084 | 1.04 | 0.99 - 1.09 | 0.11 | 1.06 | 1.05 - 1.07 | **2.6E-17** |
|  | rs10883437 | 10 | 101795361 | T | 0.61 | Model 3 | 1.08 | 1.06 - 1.09 | **1.1E-18** | 1.03 | 1.00 - 1.07 | 0.076 | 1.05 | 0.99 - 1.10 | 0.084 | 1.07 | 1.05 - 1.08 | **7.3E-19** |
| ERLIN1 | rs11597390 | 10 | 101861435 | G | 0.64 | Model 1 | 1.11 | 1.09 - 1.12 | **4.3E-37** | 1.03 | 0.99 - 1.07 | 0.19 | 1.04 | 0.99 - 1.09 | 0.14 | 1.09 | 1.08 - 1.11 | **1.2E-34** |
|  | rs11597390 | 10 | 101861435 | G | 0.64 | Model 2 | 1.11 | 1.09 - 1.13 | **2.2E-37** | 1.03 | 0.98 - 1.07 | 0.24 | 1.04 | 0.99 - 1.09 | 0.14 | 1.09 | 1.08 - 1.11 | **1.4E-34** |
|  | rs11597390 | 10 | 101861435 | G | 0.64 | Model 3 | 1.12 | 1.10 - 1.13 | **4.1E-39** | 1.03 | 0.98 - 1.07 | 0.24 | 1.04 | 0.99 - 1.10 | 0.12 | 1.1 | 1.08 - 1.11 | **3.9E-36** |
| ERLIN1 | rs11597086 | 10 | 101953705 | A | 0.58 | Model 1 | 1.14 | 1.12 - 1.16 | **2.9E-62** | 1.14 | 1.07 - 1.21 | **4.9E-05** | 1.1 | 1.04 - 1.16 | **0.00088** | 1.14 | 1.12 - 1.15 | **6.6E-68** |
|  | rs11597086 | 10 | 101953705 | A | 0.58 | Model 2 | 1.14 | 1.12 - 1.16 | **5.3E-62** | 1.13 | 1.07 - 1.21 | **8.3E-05** | 1.09 | 1.03 - 1.15 | **0.0015** | 1.14 | 1.12 - 1.15 | **4.2E-67** |
|  | rs11597086 | 10 | 101953705 | A | 0.58 | Model 3 | 1.15 | 1.13 - 1.17 | **6.9E-67** | 1.15 | 1.08 - 1.22 | **2.6E-05** | 1.1 | 1.04 - 1.16 | **0.0011** | 1.15 | 1.13 - 1.16 | **1.5E-72** |
| ERLIN1 | rs11591741 | 10 | 101976501 | G | 0.58 | Model 1 | 1.14 | 1.12 - 1.16 | **1.7E-62** | 1.11 | 1.05 - 1.17 | **0.00013** | 1.1 | 1.04 - 1.16 | **0.00045** | 1.13 | 1.12 - 1.15 | **5.4E-68** |
|  | rs11591741 | 10 | 101976501 | G | 0.58 | Model 2 | 1.14 | 1.12 - 1.16 | **3.6E-62** | 1.11 | 1.05 - 1.17 | **0.00019** | 1.1 | 1.04 - 1.16 | **0.00075** | 1.14 | 1.12 - 1.15 | **3.5E-67** |
|  | rs11591741 | 10 | 101976501 | G | 0.58 | Model 3 | 1.15 | 1.13 - 1.17 | **4.8E-67** | 1.12 | 1.06 - 1.18 | **7.7E-05** | 1.11 | 1.05 - 1.17 | **0.0005** | 1.15 | 1.13 - 1.16 | **1.5E-72** |
| TM6SF2 | rs2228603 | 19 | 19329924 | T | 0.08 | Model 1 | 1.19 | 1.16 - 1.23 | **2.1E-34** | 0.97 | 0.84 - 1.12 | 0.68 | 1.14 | 0.99 - 1.31 | 0.068 | 1.18 | 1.15 - 1.21 | **2.2E-33** |
|  | rs2228603 | 19 | 19329924 | T | 0.08 | Model 2 | 1.2 | 1.16 - 1.23 | **2.9E-35** | 0.96 | 0.84 - 1.11 | 0.6 | 1.12 | 0.98 - 1.29 | 0.099 | 1.18 | 1.15 - 1.22 | **7.1E-34** |
|  | rs2228603 | 19 | 19329924 | T | 0.08 | Model 3 | 1.23 | 1.20 - 1.27 | **1.4E-43** | 0.98 | 0.85 - 1.13 | 0.74 | 1.17 | 1.02 - 1.35 | 0.03 | 1.22 | 1.18 - 1.25 | **1.8E-42** |
| TM6SF2 | rs58542926 | 19 | 19379549 | T | 0.07 | Model 1 | 1.24 | 1.20 - 1.27 | **7.3E-50** | 1.07 | 0.98 - 1.17 | 0.13 | 1.16 | 1.03 - 1.29 | 0.011 | 1.22 | 1.19 - 1.25 | **1.3E-49** |
|  | rs58542926 | 19 | 19379549 | T | 0.07 | Model 2 | 1.24 | 1.21 - 1.28 | **6.2E-51** | 1.07 | 0.98 - 1.17 | 0.13 | 1.17 | 1.05 - 1.31 | 0.0064 | 1.22 | 1.19 - 1.26 | **8.4E-51** |
|  | rs58542926 | 19 | 19379549 | T | 0.07 | Model 3 | 1.29 | 1.25 - 1.33 | **3.9E-65** | 1.08 | 0.99 - 1.18 | 0.092 | 1.23 | 1.09 - 1.38 | **0.00073** | 1.27 | 1.23 - 1.30 | **2.8E-65** |
| PNPLA3 | rs738409 | 22 | 44324727 | G | 0.23 | Model 1 | 1.31 | 1.28 - 1.33 | **3.2E-191** | 1.19 | 1.14 - 1.25 | **2.1E-13** | 1.4 | 1.33 - 1.47 | **5.6E-40** | 1.3 | 1.28 - 1.32 | **5.5E-236** |
|  | rs738409 | 22 | 44324727 | G | 0.23 | Model 2 | 1.31 | 1.29 - 1.33 | **2.4E-190** | 1.19 | 1.14 - 1.25 | **1.7E-13** | 1.41 | 1.34 - 1.48 | **2.8E-40** | 1.3 | 1.28 - 1.33 | **2.4E-235** |
|  | rs738409 | 22 | 44324727 | G | 0.23 | Model 3 | 1.35 | 1.32 - 1.37 | **1.8E-216** | 1.2 | 1.15 - 1.26 | **2.3E-14** | 1.46 | 1.39 - 1.54 | **6.9E-46** | 1.34 | 1.32 - 1.36 | **2.7E-266** |
| PNPLA3 | rs2281135 | 22 | 44332570 | A | 0.17 | Model 1 | 1.26 | 1.24 - 1.29 | **1.4E-118** | 1.17 | 1.12 - 1.22 | **1.2E-11** | 1.33 | 1.26 - 1.40 | **1.3E-27** | 1.26 | 1.23 - 1.28 | **3.1E-151** |
|  | rs2281135 | 22 | 44332570 | A | 0.17 | Model 2 | 1.26 | 1.24 - 1.29 | **5.6E-117** | 1.17 | 1.12 - 1.22 | **1.9E-11** | 1.34 | 1.27 - 1.41 | **4.8E-28** | 1.26 | 1.24 - 1.28 | **1.3E-149** |
|  | rs2281135 | 22 | 44332570 | A | 0.17 | Model 3 | 1.3 | 1.27 - 1.33 | **4.3E-136** | 1.18 | 1.13 - 1.23 | **3.7E-12** | 1.38 | 1.31 - 1.46 | **1.4E-32** | 1.29 | 1.27 - 1.31 | **1.4E-172** |
| PNPLA3 | rs2143571 | 22 | 44391686 | A | 0.18 | Model 1 | 1.19 | 1.17 - 1.22 | **8.3E-73** | 1.04 | 1.01 - 1.08 | 0.016 | 1.21 | 1.15 - 1.28 | **1.5E-12** | 1.16 | 1.14 - 1.18 | **7.5E-75** |
|  | rs2143571 | 22 | 44391686 | A | 0.18 | Model 2 | 1.19 | 1.17 - 1.22 | **1.6E-71** | 1.04 | 1.01 - 1.08 | 0.017 | 1.22 | 1.15 - 1.28 | **6.9E-13** | 1.16 | 1.14 - 1.18 | **1.1E-73** |
|  | rs2143571 | 22 | 44391686 | A | 0.18 | Model 3 | 1.22 | 1.20 - 1.25 | **2.9E-84** | 1.04 | 1.01 - 1.08 | 0.018 | 1.23 | 1.17 - 1.30 | **1.3E-13** | 1.18 | 1.16 - 1.20 | **2.2E-84** |

**ABALT2**: ALT >40 U/L for men, >30 U/L for women ≥ 6 months apart over 2 years, no viral hepatitis, and no chronic liver disease with ALT ≤40 U/L for men and ≤30 U/L for women as controls. **Abbreviations:** rsID: dbSNP identifier (build 151), Chr: chromosome, Pos: basepair position on human genome reference hg19, EA: effect allele, EAF: effect allele frequency among Europeans (Million Veteran Program), OR: odds ratio of risk in cases compared to controls per effect allele (additive model), CI: confidence interval. **Model 1:** adjusted for age, gender, and 10 principal components (PCs), **Model 2:** covariates in Model 1 + alcohol consumption at enrollment measured by the Alcohol Use Disorder Identification Test (AUDIT-C), **Model 3:** covariates in Model 2 + Type II diabetes/prediabetes, hypertension, dyslipidemia and BMI ≥ 30 kg/m2. P-values below 0.006 (adjusted for multiple comparisons) are shown in **bold font**.

**Table S6c.** Previously published NAFLD risk/ALT level-associated variants with genome-wide significance and association with NAFLD phenotype, **ALT2DL**

|  |  |  |  |  |  |  | **European Ancestry**  **(n=31,900 cases, 101,701 controls)** | | | **African Ancestry**  **(n=4,693 cases, 23,859 controls)** | | | **Hispanic/Latino Ancestry**  **(n=4,374 cases, 6,514 controls)** | | | **Transethnic Meta-Analysis**  **(n=40,967 cases, 132,074 controls)** | | |
| --- | --- | --- | --- | --- | --- | --- | --- | --- | --- | --- | --- | --- | --- | --- | --- | --- | --- | --- |
| **Gene** | **rsID** | **Chr** | **Pos** | **EA** | **EAF** | **ADJUSTED** | **OR** | **95% CI** | **P** | **OR** | **95% CI** | **P** | **OR** | **95% CI** | **P** | **OR** | **95% CI** | **P** |
| LYPLAL1 | rs12137855 | 1 | 219448378 | C | 0.8 | Model 1 | 1.01 | 0.99 - 1.03 | 0.39 | 0.96 | 0.91 - 1.01 | 0.15 | 0.99 | 0.92 - 1.08 | 0.89 | 1 | 0.98 - 1.02 | 0.85 |
|  | rs12137855 | 1 | 219448378 | C | 0.8 | Model 2 | 1.01 | 0.99 - 1.03 | 0.41 | 0.96 | 0.91 - 1.01 | 0.12 | 1 | 0.92 - 1.08 | 0.96 | 1 | 0.98 - 1.02 | 0.88 |
| LYPLAL1 | rs3001032 | 1 | 219727779 | T | 0.69 | Model 1 | 1.06 | 1.04 - 1.08 | **2.4E-09** | 0.98 | 0.94 - 1.02 | 0.31 | 1.05 | 0.99 - 1.11 | 0.11 | 1.04 | 1.03 - 1.06 | **1.9E-07** |
|  | rs3001032 | 1 | 219727779 | T | 0.69 | Model 2 | 1.06 | 1.04 - 1.08 | **1.9E-08** | 0.98 | 0.94 - 1.02 | 0.33 | 1.05 | 0.99 - 1.11 | 0.11 | 1.04 | 1.03 - 1.06 | **9.4E-07** |
| GCKR | rs780094 | 2 | 27741237 | T | 0.4 | Model 1 | 1.05 | 1.03 - 1.07 | **6.5E-08** | 1.04 | 0.99 - 1.10 | 0.12 | 1.04 | 0.98 - 1.11 | 0.16 | 1.05 | 1.03 - 1.07 | **7.0E-09** |
|  | rs780094 | 2 | 27741237 | T | 0.4 | Model 2 | 1.05 | 1.03 - 1.06 | **7.7E-07** | 1.04 | 0.98 - 1.09 | 0.17 | 1.03 | 0.97 - 1.10 | 0.3 | 1.04 | 1.03 - 1.06 | **1.9E-07** |
| HSD17B13 | rs6834314 | 4 | 88213808 | A | 0.72 | Model 1 | 1.08 | 1.05 - 1.10 | **1.7E-13** | 1.02 | 0.98 - 1.07 | 0.35 | 1.09 | 1.00 - 1.18 | 0.042 | 1.07 | 1.05 - 1.09 | **9.3E-14** |
|  | rs6834314 | 4 | 88213808 | A | 0.72 | Model 2 | 1.08 | 1.05 - 1.10 | **2.9E-13** | 1.02 | 0.97 - 1.07 | 0.41 | 1.09 | 1.01 - 1.18 | 0.03 | 1.07 | 1.05 - 1.09 | **1.7E-13** |
| HSD17B13 | rs72613567 | 4 | 88231392 | T | 0.73 | Model 1 | 1.08 | 1.06 - 1.10 | **3.3E-14** | 1.03 | 0.95 - 1.11 | 0.45 | 1.12 | 1.03 - 1.21 | 0.0092 | 1.08 | 1.06 - 1.10 | **2.2E-15** |
|  | rs72613567 | 4 | 88231392 | T | 0.73 | Model 2 | 1.08 | 1.06 - 1.10 | **9.5E-14** | 1.02 | 0.95 - 1.11 | 0.56 | 1.13 | 1.03 - 1.22 | 0.0058 | 1.08 | 1.06 - 1.10 | **6.6E-15** |
| PPP1R3B | rs4240624 | 8 | 9184231 | G | 0.09 | Model 1 | 1.15 | 1.12 - 1.19 | **6.3E-20** | 1.09 | 1.03 - 1.14 | **0.0012** | 1 | 0.93 - 1.08 | 0.96 | 1.12 | 1.09 - 1.14 | **2.8E-19** |
|  | rs4240624 | 8 | 9184231 | G | 0.09 | Model 2 | 1.15 | 1.11 - 1.18 | **3.3E-19** | 1.08 | 1.03 - 1.14 | **0.0018** | 1 | 0.93 - 1.07 | 0.97 | 1.12 | 1.09 - 1.14 | **2.5E-18** |
| TRIB1 | rs2954021 | 8 | 126482077 | A | 0.5 | Model 1 | 1.17 | 1.15 - 1.19 | **9.2E-70** | 1.03 | 0.99 - 1.07 | 0.15 | 1.16 | 1.10 - 1.23 | 2.6E-07 | 1.15 | 1.13 - 1.17 | **3.8E-69** |
|  | rs2954021 | 8 | 126482077 | A | 0.5 | Model 2 | 1.17 | 1.15 - 1.19 | **3.6E-69** | 1.03 | 0.99 - 1.07 | 0.17 | 1.16 | 1.09 - 1.23 | 6.0E-07 | 1.15 | 1.13 - 1.17 | **5.4E-68** |
| ERLIN1 | rs10883437 | 10 | 101795361 | T | 0.61 | Model 1 | 1.08 | 1.06 - 1.10 | **5.9E-18** | 1.05 | 1.01 - 1.09 | 0.022 | 1.05 | 1.00 - 1.12 | 0.069 | 1.07 | 1.06 - 1.09 | **2.7E-19** |
|  | rs10883437 | 10 | 101795361 | T | 0.61 | Model 2 | 1.08 | 1.06 - 1.10 | **1.2E-17** | 1.05 | 1.01 - 1.09 | 0.027 | 1.05 | 0.99 - 1.11 | 0.087 | 1.07 | 1.06 - 1.09 | **9.5E-19** |
| ERLIN1 | rs11597390 | 10 | 101861435 | G | 0.64 | Model 1 | 1.12 | 1.10 - 1.15 | **2.0E-37** | 1.07 | 1.01 - 1.12 | 0.011 | 1.03 | 0.97 - 1.10 | 0.28 | 1.11 | 1.09 - 1.13 | **7.3E-37** |
|  | rs11597390 | 10 | 101861435 | G | 0.64 | Model 2 | 1.13 | 1.11 - 1.15 | **2.0E-37** | 1.06 | 1.01 - 1.12 | 0.017 | 1.03 | 0.97 - 1.10 | 0.28 | 1.11 | 1.09 - 1.13 | **1.6E-36** |
| ERLIN1 | rs11597086 | 10 | 101953705 | A | 0.58 | Model 1 | 1.16 | 1.14 - 1.18 | **2.8E-60** | 1.14 | 1.06 - 1.23 | **0.00048** | 1.09 | 1.02 - 1.16 | 0.01 | 1.15 | 1.13 - 1.17 | **1.4E-63** |
|  | rs11597086 | 10 | 101953705 | A | 0.58 | Model 2 | 1.16 | 1.14 - 1.18 | **1.3E-59** | 1.14 | 1.05 - 1.22 | **0.00086** | 1.09 | 1.02 - 1.16 | 0.012 | 1.15 | 1.13 - 1.17 | **1.5E-62** |
| ERLIN1 | rs11591741 | 10 | 101976501 | G | 0.58 | Model 1 | 1.16 | 1.14 - 1.18 | **1.0E-60** | 1.13 | 1.06 - 1.21 | **0.00026** | 1.09 | 1.02 - 1.16 | 0.0085 | 1.15 | 1.13 - 1.17 | **2.6E-64** |
|  | rs11591741 | 10 | 101976501 | G | 0.58 | Model 2 | 1.16 | 1.14 - 1.18 | **5.5E-60** | 1.13 | 1.06 - 1.20 | **0.00036** | 1.09 | 1.02 - 1.16 | 0.0093 | 1.15 | 1.13 - 1.17 | **2.4E-63** |
| TM6SF2 | rs2228603 | 19 | 19329924 | T | 0.08 | Model 1 | 1.16 | 1.12 - 1.20 | **4.6E-18** | 0.86 | 0.72 - 1.02 | 0.091 | 0.97 | 0.82 - 1.14 | 0.69 | 1.14 | 1.10 - 1.17 | **1.5E-15** |
|  | rs2228603 | 19 | 19329924 | T | 0.08 | Model 2 | 1.16 | 1.13 - 1.20 | **5.0E-19** | 0.85 | 0.72 - 1.02 | 0.079 | 0.96 | 0.81 - 1.14 | 0.65 | 1.14 | 1.11 - 1.18 | **2.7E-16** |
| TM6SF2 | rs58542926 | 19 | 19379549 | T | 0.07 | Model 1 | 1.18 | 1.14 - 1.22 | **1.9E-23** | 1 | 0.90 - 1.12 | 0.99 | 1.04 | 0.90 - 1.19 | 0.6 | 1.16 | 1.12 - 1.20 | **4.1E-21** |
|  | rs58542926 | 19 | 19379549 | T | 0.07 | Model 2 | 1.19 | 1.15 - 1.23 | **1.3E-24** | 1 | 0.90 - 1.12 | 0.97 | 1.06 | 0.93 - 1.22 | 0.38 | 1.17 | 1.13 - 1.20 | **1.8E-22** |
| PNPLA3 | rs738409 | 22 | 44324727 | G | 0.23 | Model 1 | 1.29 | 1.27 - 1.32 | **3.3E-132** | 1.19 | 1.13 - 1.26 | **4.5E-10** | 1.33 | 1.25 - 1.41 | 6.2E-21 | 1.28 | 1.26 - 1.31 | **4.9E-158** |
|  | rs738409 | 22 | 44324727 | G | 0.23 | Model 2 | 1.3 | 1.27 - 1.32 | **1.6E-131** | 1.19 | 1.13 - 1.26 | **4.3E-10** | 1.33 | 1.25 - 1.41 | 4.2E-21 | 1.29 | 1.26 - 1.31 | **1.8E-157** |
| PNPLA3 | rs2281135 | 22 | 44332570 | A | 0.17 | Model 1 | 1.25 | 1.22 - 1.28 | **1.0E-80** | 1.18 | 1.12 - 1.24 | **3.8E-09** | 1.26 | 1.19 - 1.34 | 2.1E-14 | 1.24 | 1.22 - 1.27 | **4.7E-100** |
|  | rs2281135 | 22 | 44332570 | A | 0.17 | Model 2 | 1.25 | 1.22 - 1.28 | **1.3E-79** | 1.18 | 1.11 - 1.24 | **6.2E-09** | 1.27 | 1.20 - 1.35 | 7.7E-15 | 1.24 | 1.22 - 1.27 | **4.4E-99** |
| PNPLA3 | rs2143571 | 22 | 44391686 | A | 0.18 | Model 1 | 1.17 | 1.15 - 1.20 | **4.9E-45** | 1.05 | 1.00 - 1.09 | 0.029 | 1.19 | 1.12 - 1.27 | 2.4E-08 | 1.15 | 1.13 - 1.17 | **1.4E-47** |
|  | rs2143571 | 22 | 44391686 | A | 0.18 | Model 2 | 1.17 | 1.15 - 1.20 | **1.6E-43** | 1.05 | 1.00 - 1.09 | 0.035 | 1.2 | 1.13 - 1.28 | 6.3E-09 | 1.15 | 1.13 - 1.17 | **2.1E-46** |

**ALT2DL:** ALT >40 for men, >30 for women ≥ 6 months apart over 2 years, no viral hepatitis, and no chronic liver disease + dyslipidemia with ALT≤30 U/L for men and ≤20 U/L for women as controls. **Abbreviations:** rsID: dbSNP identifier (build 151), Chr: chromosome, Pos: basepair position on human genome reference hg19, EA: effect allele, EAF: effect allele frequency among Europeans (Million Veteran Program), OR: odds ratio of risk in cases compared to controls per effect allele (additive model), CI: confidence interval. **Model 1:** adjusted for age, gender, and 10 principal components (PCs), **Model 2:** covariates in Model 1 + alcohol consumption at enrollment measured by the Alcohol Use Disorder Identification Test (AUDIT-C). P-values below 0.006 (adjusted for multiple comparisons) are shown in **bold font**.

**Table S6d.** Previously published NAFLD risk /ALT level-associated variants with genome-wide significance and association with NAFLD phenotype, **ALT2DM**

|  |  |  |  |  |  |  | **European Ancestry**  **(n=31,879 cases, 101,701 controls)** | | | **African Ancestry**  **(n=5,478 cases, 23,859 controls)** | | | **Hispanic/Latino Ancestry**  **(n=4,114 cases, 6,514 controls)** | | | **Transethnic Meta-Analysis (n=41,371 cases, 132,074 controls)** | | |
| --- | --- | --- | --- | --- | --- | --- | --- | --- | --- | --- | --- | --- | --- | --- | --- | --- | --- | --- |
| **Gene** | **rsID** | **Chr** | **Pos** | **EA** | **EAF** | **ADJUSTED** | **OR** | **95% CI** | **P** | **OR** | **95% CI** | **P** | **OR** | **95% CI** | **P** | **OR** | **95% CI** | **P** |
| LYPLAL1 | rs12137855 | 1 | 219448378 | C | 0.8 | Model 1 | 1 | 0.98 - 1.02 | 0.85 | 0.96 | 0.91 - 1.02 | 0.17 | 1 | 0.92 - 1.08 | 0.91 | 1 | 0.98 - 1.02 | 0.7 |
|  | rs12137855 | 1 | 219448378 | C | 0.8 | Model 2 | 1 | 0.98 - 1.03 | 0.79 | 0.96 | 0.91 - 1.01 | 0.14 | 1 | 0.92 - 1.09 | 0.96 | 1 | 0.98 - 1.02 | 0.75 |
| LYPLAL1 | rs3001032 | 1 | 219727779 | T | 0.69 | Model 1 | 1.05 | 1.03 - 1.07 | **1.7E-06** | 0.97 | 0.94 - 1.01 | 0.18 | 1.06 | 1.00 - 1.13 | 0.042 | 1.04 | 1.02 - 1.05 | **3.8E-05** |
|  | rs3001032 | 1 | 219727779 | T | 0.69 | Model 2 | 1.05 | 1.03 - 1.07 | **2.0E-06** | 0.97 | 0.94 - 1.01 | 0.17 | 1.06 | 1.00 - 1.13 | 0.046 | 1.04 | 1.02 - 1.05 | **4.9E-05** |
| GCKR | rs780094 | 2 | 27741237 | T | 0.4 | Model 1 | 1.02 | 1.00 - 1.04 | 0.072 | 1.01 | 0.96 - 1.06 | 0.7 | 1.01 | 0.95 - 1.07 | 0.75 | 1.02 | 1.00 - 1.03 | 0.065 |
|  | rs780094 | 2 | 27741237 | T | 0.4 | Model 2 | 1.01 | 1.00 - 1.03 | 0.14 | 1.01 | 0.96 - 1.06 | 0.77 | 1 | 0.94 - 1.07 | 0.93 | 1.01 | 1.00 - 1.03 | 0.15 |
| HSD17B13 | rs6834314 | 4 | 88213808 | A | 0.72 | Model 1 | 1.09 | 1.07 - 1.11 | **1.8E-16** | 1.02 | 0.98 - 1.07 | 0.36 | 1.06 | 0.98 - 1.15 | 0.16 | 1.08 | 1.06 - 1.10 | **7.8E-16** |
|  | rs6834314 | 4 | 88213808 | A | 0.72 | Model 2 | 1.09 | 1.07 - 1.11 | **9.1E-17** | 1.02 | 0.97 - 1.07 | 0.42 | 1.07 | 0.98 - 1.16 | 0.12 | 1.08 | 1.06 - 1.10 | **5.2E-16** |
| HSD17B13 | rs72613567 | 4 | 88231392 | T | 0.73 | Model 1 | 1.09 | 1.07 - 1.11 | **1.4E-17** | 1.07 | 0.99 - 1.16 | 0.1 | 1.08 | 0.99 - 1.17 | 0.094 | 1.09 | 1.07 - 1.11 | **1.1E-18** |
|  | rs72613567 | 4 | 88231392 | T | 0.73 | Model 2 | 1.09 | 1.07 - 1.12 | **1.5E-17** | 1.06 | 0.98 - 1.15 | 0.14 | 1.08 | 1.00 - 1.18 | 0.063 | 1.09 | 1.07 - 1.11 | **1.1E-18** |
| PPP1R3B | rs4240624 | 8 | 9184231 | G | 0.09 | Model 1 | 1.17 | 1.13 - 1.20 | **5.1E-23** | 1.07 | 1.02 - 1.13 | **0.0055** | 0.99 | 0.92 - 1.06 | 0.74 | 1.12 | 1.10 - 1.15 | **3.8E-20** |
|  | rs4240624 | 8 | 9184231 | G | 0.09 | Model 2 | 1.17 | 1.13 - 1.21 | **8.5E-23** | 1.07 | 1.02 - 1.12 | 0.0084 | 0.99 | 0.91 - 1.06 | 0.71 | 1.12 | 1.09 - 1.15 | **1.3E-19** |
| TRIB1 | rs2954021 | 8 | 126482077 | A | 0.5 | Model 1 | 1.13 | 1.11 - 1.15 | **8.3E-39** | 1.03 | 0.99 - 1.07 | 0.15 | 1.13 | 1.06 - 1.19 | **6.9E-05** | 1.11 | 1.09 - 1.13 | **2.5E-39** |
|  | rs2954021 | 8 | 126482077 | A | 0.5 | Model 2 | 1.13 | 1.11 - 1.15 | **6.6E-38** | 1.03 | 0.99 - 1.07 | 0.16 | 1.13 | 1.06 - 1.19 | **7.8E-05** | 1.11 | 1.09 - 1.13 | **2.6E-38** |
| ERLIN1 | rs10883437 | 10 | 101795361 | T | 0.61 | Model 1 | 1.08 | 1.06 - 1.10 | **4.4E-18** | 1.03 | 0.99 - 1.07 | 0.13 | 1.06 | 1.00 - 1.12 | 0.046 | 1.07 | 1.06 - 1.09 | **3.0E-18** |
|  | rs10883437 | 10 | 101795361 | T | 0.61 | Model 2 | 1.08 | 1.06 - 1.10 | **9.6E-18** | 1.03 | 0.99 - 1.07 | 0.13 | 1.06 | 1.00 - 1.13 | 0.045 | 1.07 | 1.06 - 1.09 | **5.6E-18** |
| ERLIN1 | rs11597390 | 10 | 101861435 | G | 0.64 | Model 1 | 1.13 | 1.11 - 1.15 | **1.0E-39** | 1.04 | 0.99 - 1.09 | 0.17 | 1.03 | 0.97 - 1.09 | 0.36 | 1.11 | 1.09 - 1.13 | **3.0E-36** |
|  | rs11597390 | 10 | 101861435 | G | 0.64 | Model 2 | 1.13 | 1.11 - 1.16 | **2.3E-39** | 1.03 | 0.98 - 1.08 | 0.21 | 1.03 | 0.97 - 1.09 | 0.36 | 1.11 | 1.09 - 1.13 | **1.2E-35** |
| ERLIN1 | rs11597086 | 10 | 101953705 | A | 0.58 | Model 1 | 1.17 | 1.15 - 1.19 | **4.3E-62** | 1.14 | 1.05 - 1.22 | **0.00082** | 1.09 | 1.02 - 1.16 | 0.0082 | 1.16 | 1.14 - 1.18 | **3.5E-65** |
|  | rs11597086 | 10 | 101953705 | A | 0.58 | Model 2 | 1.17 | 1.15 - 1.19 | **2.5E-61** | 1.13 | 1.05 - 1.22 | **0.0011** | 1.09 | 1.02 - 1.16 | 0.012 | 1.16 | 1.14 - 1.18 | **4.9E-64** |
| ERLIN1 | rs11591741 | 10 | 101976501 | G | 0.58 | Model 1 | 1.17 | 1.15 - 1.19 | **4.7E-62** | 1.09 | 1.02 - 1.16 | 0.0072 | 1.1 | 1.03 - 1.17 | **0.0047** | 1.16 | 1.14 - 1.18 | **5.4E-64** |
|  | rs11591741 | 10 | 101976501 | G | 0.58 | Model 2 | 1.17 | 1.15 - 1.19 | **3.1E-61** | 1.09 | 1.02 - 1.16 | 0.0084 | 1.09 | 1.03 - 1.17 | 0.0068 | 1.16 | 1.14 - 1.18 | **7.7E-63** |
| TM6SF2 | rs2228603 | 19 | 19329924 | T | 0.08 | Model 1 | 1.2 | 1.16 - 1.24 | **5.6E-27** | 0.93 | 0.78 - 1.10 | 0.38 | 1.11 | 0.94 - 1.30 | 0.24 | 1.19 | 1.15 - 1.23 | **2.2E-25** |
|  | rs2228603 | 19 | 19329924 | T | 0.08 | Model 2 | 1.21 | 1.17 - 1.25 | **5.4E-28** | 0.92 | 0.77 - 1.09 | 0.33 | 1.09 | 0.93 - 1.29 | 0.29 | 1.19 | 1.15 - 1.23 | **4.1E-26** |
| TM6SF2 | rs58542926 | 19 | 19379549 | T | 0.07 | Model 1 | 1.26 | 1.21 - 1.30 | **6.9E-41** | 1.05 | 0.94 - 1.16 | 0.41 | 1.22 | 1.07 - 1.40 | **0.0035** | 1.23 | 1.20 - 1.27 | **1.1E-40** |
|  | rs58542926 | 19 | 19379549 | T | 0.07 | Model 2 | 1.26 | 1.22 - 1.31 | **5.7E-42** | 1.05 | 0.94 - 1.17 | 0.37 | 1.24 | 1.08 - 1.42 | **0.0022** | 1.24 | 1.20 - 1.28 | **6.1E-42** |
| PNPLA3 | rs738409 | 22 | 44324727 | G | 0.23 | Model 1 | 1.32 | 1.29 - 1.35 | **1.6E-147** | 1.24 | 1.17 - 1.31 | **1.1E-14** | 1.42 | 1.33 - 1.50 | **4.5E-30** | 1.32 | 1.30 - 1.35 | **1.7E-186** |
|  | rs738409 | 22 | 44324727 | G | 0.23 | Model 2 | 1.32 | 1.30 - 1.35 | **2.9E-146** | 1.24 | 1.18 - 1.31 | **7.4E-15** | 1.42 | 1.33 - 1.51 | **8.8E-30** | 1.32 | 1.30 - 1.35 | **3.3E-185** |
| PNPLA3 | rs2281135 | 22 | 44332570 | A | 0.17 | Model 1 | 1.27 | 1.24 - 1.30 | **5.0E-89** | 1.22 | 1.15 - 1.29 | **5.4E-13** | 1.34 | 1.26 - 1.43 | **6.4E-21** | 1.27 | 1.24 - 1.30 | **2.3E-118** |
|  | rs2281135 | 22 | 44332570 | A | 0.17 | Model 2 | 1.27 | 1.24 - 1.30 | **1.7E-87** | 1.22 | 1.15 - 1.29 | **7.0E-13** | 1.34 | 1.26 - 1.43 | **1.0E-20** | 1.27 | 1.25 - 1.30 | **1.6E-116** |
| PNPLA3 | rs2143571 | 22 | 44391686 | A | 0.18 | Model 1 | 1.19 | 1.17 - 1.22 | **9.7E-52** | 1.06 | 1.02 - 1.10 | **0.0062** | 1.23 | 1.15 - 1.31 | **3.2E-10** | 1.17 | 1.14 - 1.19 | **4.3E-56** |
|  | rs2143571 | 22 | 44391686 | A | 0.18 | Model 2 | 1.19 | 1.16 - 1.22 | **4.4E-50** | 1.06 | 1.01 - 1.10 | **0.0082** | 1.23 | 1.15 - 1.31 | **1.9E-10** | 1.16 | 1.14 - 1.19 | **2.1E-54** |

**ALT2DM:** ALT >40 for men, >30 for women ≥ 6 months apart over 2 years, no viral hepatitis, and no chronic liver disease + Type II diabetes or prediabetes with ALT≤30 U/L for men and ≤20 U/L for women as controls. **Abbreviations:** rsID: dbSNP identifier (build 151), Chr: chromosome, Pos: basepair position on human genome reference hg19, EA: effect allele, EAF: effect allele frequency among Europeans (Million Veteran Program), OR: odds ratio of risk in cases compared to controls per effect allele (additive model), CI: confidence interval. **Model 1:** adjusted for age, gender, and 10 principal components (PCs), **Model 2:** covariates in Model 1 + alcohol consumption at enrollment measured by the Alcohol Use Disorder Identification Test (AUDIT-C). P-values below 0.006 (adjusted for multiple comparisons) are shown in **bold font**.

**Table S6e.** Previously published NAFLD risk/ALT level-associated variants with genome-wide significance and association with NAFLD phenotype, **ALT2HTN**

|  |  |  |  |  |  |  | **European Ancestry**  **(n=38,375 cases, 101,701 controls)** | | | **African Ancestry**  **(n=6,921 cases, 23,859 controls)** | | | **Hispanic/Latino Ancestry**  **(n=3,825 cases, 6,514 controls)** | | | **Transethnic Meta-Analysis (n=49,423 cases, 132,074 controls)** | | |
| --- | --- | --- | --- | --- | --- | --- | --- | --- | --- | --- | --- | --- | --- | --- | --- | --- | --- | --- |
| **Gene** | **rsID** | **Chr** | **Pos** | **EA** | **EAF** | **ADJUSTED** | **OR** | **95% CI** | **P** | **OR** | **95% CI** | **P** | **OR** | **95% CI** | **P** | **OR** | **95% CI** | **P** |
| LYPLAL1 | rs12137855 | 1 | 219448378 | C | 0.8 | Model 1 | 1.01 | 0.98 - 1.03 | 0.63 | 0.95 | 0.91 - 1.00 | 0.043 | 1 | 0.92 - 1.08 | 0.96 | 1 | 0.98 - 1.02 | 0.71 |
|  | rs12137855 | 1 | 219448378 | C | 0.8 | Model 2 | 1 | 0.98 - 1.03 | 0.68 | 0.95 | 0.90 - 1.00 | 0.039 | 1 | 0.93 - 1.09 | 0.91 | 1 | 0.98 - 1.02 | 0.68 |
| LYPLAL1 | rs3001032 | 1 | 219727779 | T | 0.69 | Model 1 | 1.05 | 1.03 - 1.07 | **5.3E-08** | 0.97 | 0.94 - 1.01 | 0.12 | 1.06 | 1.00 - 1.12 | 0.059 | 1.04 | 1.02 - 1.05 | **6.4E-06** |
|  | rs3001032 | 1 | 219727779 | T | 0.69 | Model 2 | 1.05 | 1.03 - 1.07 | **5.0E-08** | 0.97 | 0.93 - 1.01 | 0.1 | 1.05 | 0.99 - 1.12 | 0.074 | 1.04 | 1.02 - 1.05 | **8.4E-06** |
| GCKR | rs780094 | 2 | 27741237 | T | 0.4 | Model 1 | 1.01 | 1.00 - 1.03 | 0.098 | 1.01 | 0.96 - 1.06 | 0.67 | 1.01 | 0.95 - 1.07 | 0.82 | 1.01 | 1.00 - 1.03 | 0.089 |
|  | rs780094 | 2 | 27741237 | T | 0.4 | Model 2 | 1.01 | 0.99 - 1.03 | 0.23 | 1.01 | 0.96 - 1.06 | 0.79 | 1 | 0.94 - 1.06 | 0.92 | 1.01 | 0.99 - 1.03 | 0.25 |
| HSD17B13 | rs6834314 | 4 | 88213808 | A | 0.72 | Model 1 | 1.09 | 1.07 - 1.11 | **6.9E-18** | 1.02 | 0.98 - 1.07 | 0.33 | 1.09 | 1.01 - 1.18 | 0.03 | 1.08 | 1.06 - 1.10 | **1.0E-17** |
|  | rs6834314 | 4 | 88213808 | A | 0.72 | Model 2 | 1.09 | 1.07 - 1.11 | **7.7E-18** | 1.02 | 0.98 - 1.07 | 0.37 | 1.1 | 1.01 - 1.19 | 0.021 | 1.08 | 1.06 - 1.10 | **1.2E-17** |
| HSD17B13 | rs72613567 | 4 | 88231392 | T | 0.73 | Model 1 | 1.09 | 1.07 - 1.11 | **4.8E-19** | 1.08 | 1.00 - 1.16 | 0.051 | 1.13 | 1.03 - 1.22 | 0.0063 | 1.09 | 1.07 - 1.11 | **2.2E-21** |
|  | rs72613567 | 4 | 88231392 | T | 0.73 | Model 2 | 1.09 | 1.07 - 1.11 | **6.8E-19** | 1.07 | 0.99 - 1.15 | 0.076 | 1.13 | 1.04 - 1.23 | **0.004** | 1.09 | 1.07 - 1.11 | **3.6E-21** |
| PPP1R3B | rs4240624 | 8 | 9184231 | G | 0.09 | Model 1 | 1.16 | 1.13 - 1.20 | **7.4E-24** | 1.08 | 1.03 - 1.14 | **0.00076** | 1.03 | 0.96 - 1.11 | 0.37 | 1.13 | 1.10 - 1.16 | **8.8E-24** |
|  | rs4240624 | 8 | 9184231 | G | 0.09 | Model 2 | 1.16 | 1.13 - 1.20 | **4.6E-23** | 1.08 | 1.03 - 1.13 | 0.001 | 1.03 | 0.96 - 1.11 | 0.43 | 1.13 | 1.10 - 1.15 | **9.3E-23** |
| TRIB1 | rs2954021 | 8 | 126482077 | A | 0.5 | Model 1 | 1.13 | 1.11 - 1.15 | **3.3E-46** | 1.03 | 0.99 - 1.07 | 0.11 | 1.15 | 1.08 - 1.21 | **4.4E-06** | 1.12 | 1.10 - 1.13 | **5.6E-47** |
|  | rs2954021 | 8 | 126482077 | A | 0.5 | Model 2 | 1.13 | 1.11 - 1.15 | **9.8E-46** | 1.03 | 0.99 - 1.07 | 0.11 | 1.14 | 1.08 - 1.21 | **9.7E-06** | 1.12 | 1.10 - 1.13 | **2.7E-46** |
| ERLIN1 | rs10883437 | 10 | 101795361 | T | 0.61 | Model 1 | 1.08 | 1.06 - 1.10 | **2.0E-17** | 1.03 | 1.00 - 1.07 | 0.071 | 1.05 | 1.00 - 1.12 | 0.071 | 1.07 | 1.05 - 1.09 | **6.0E-18** |
|  | rs10883437 | 10 | 101795361 | T | 0.61 | Model 2 | 1.08 | 1.06 - 1.10 | **4.7E-17** | 1.03 | 1.00 - 1.07 | 0.076 | 1.05 | 0.99 - 1.11 | 0.091 | 1.07 | 1.05 - 1.09 | **2.0E-17** |
| ERLIN1 | rs11597390 | 10 | 101861435 | G | 0.64 | Model 1 | 1.13 | 1.11 - 1.15 | **1.1E-40** | 1.05 | 1.00 - 1.10 | 0.042 | 1.05 | 0.99 - 1.12 | 0.099 | 1.11 | 1.10 - 1.13 | **1.4E-39** |
|  | rs11597390 | 10 | 101861435 | G | 0.64 | Model 2 | 1.13 | 1.11 - 1.15 | **2.5E-40** | 1.05 | 1.00 - 1.10 | 0.054 | 1.05 | 0.99 - 1.12 | 0.11 | 1.11 | 1.10 - 1.13 | **6.5E-39** |
| ERLIN1 | rs11597086 | 10 | 101953705 | A | 0.58 | Model 1 | 1.16 | 1.14 - 1.18 | **1.4E-65** | 1.17 | 1.09 - 1.25 | **1.2E-05** | 1.1 | 1.03 - 1.17 | **0.0041** | 1.16 | 1.14 - 1.18 | **6.0E-71** |
|  | rs11597086 | 10 | 101953705 | A | 0.58 | Model 2 | 1.16 | 1.14 - 1.18 | **2.1E-64** | 1.17 | 1.09 - 1.25 | **2.1E-05** | 1.1 | 1.03 - 1.17 | **0.0058** | 1.16 | 1.14 - 1.18 | **2.5E-69** |
| ERLIN1 | rs11591741 | 10 | 101976501 | G | 0.58 | Model 1 | 1.16 | 1.14 - 1.18 | **1.2E-65** | 1.15 | 1.08 - 1.22 | **1.0E-05** | 1.1 | 1.04 - 1.18 | **0.0023** | 1.16 | 1.14 - 1.18 | **2.1E-71** |
|  | rs11591741 | 10 | 101976501 | G | 0.58 | Model 2 | 1.16 | 1.14 - 1.18 | **2.2E-64** | 1.15 | 1.08 - 1.22 | **1.4E-05** | 1.1 | 1.03 - 1.18 | **0.0032** | 1.16 | 1.14 - 1.18 | **8.5E-70** |
| TM6SF2 | rs2228603 | 19 | 19329924 | T | 0.08 | Model 1 | 1.21 | 1.18 - 1.25 | **1.5E-32** | 0.93 | 0.80 - 1.10 | 0.4 | 1.14 | 0.97 - 1.34 | 0.11 | 1.2 | 1.16 - 1.24 | **5.5E-31** |
|  | rs2228603 | 19 | 19329924 | T | 0.08 | Model 2 | 1.22 | 1.18 - 1.26 | **2.4E-33** | 0.92 | 0.79 - 1.08 | 0.32 | 1.13 | 0.96 - 1.33 | 0.15 | 1.2 | 1.17 - 1.24 | **2.1E-31** |
| TM6SF2 | rs58542926 | 19 | 19379549 | T | 0.07 | Model 1 | 1.26 | 1.22 - 1.30 | **3.5E-46** | 1.05 | 0.95 - 1.17 | 0.3 | 1.2 | 1.05 - 1.37 | 0.0075 | 1.24 | 1.20 - 1.27 | **1.5E-45** |
|  | rs58542926 | 19 | 19379549 | T | 0.07 | Model 2 | 1.27 | 1.23 - 1.31 | **4.9E-47** | 1.05 | 0.95 - 1.17 | 0.3 | 1.22 | 1.07 - 1.40 | **0.0036** | 1.24 | 1.21 - 1.28 | **1.4E-46** |
| PNPLA3 | rs738409 | 22 | 44324727 | G | 0.23 | Model 1 | 1.31 | 1.29 - 1.34 | **1.7E-154** | 1.21 | 1.15 - 1.27 | **5.0E-13** | 1.38 | 1.30 - 1.46 | **2.6E-26** | 1.31 | 1.28 - 1.33 | **9.8E-188** |
|  | rs738409 | 22 | 44324727 | G | 0.23 | Model 2 | 1.32 | 1.29 - 1.34 | **1.2E-153** | 1.21 | 1.15 - 1.27 | **6.6E-13** | 1.38 | 1.30 - 1.47 | **2.1E-26** | 1.31 | 1.29 - 1.33 | **9.6E-187** |
| PNPLA3 | rs2281135 | 22 | 44332570 | A | 0.17 | Model 1 | 1.27 | 1.24 - 1.30 | **1.1E-95** | 1.18 | 1.12 - 1.24 | **7.6E-11** | 1.3 | 1.22 - 1.38 | **6.6E-17** | 1.26 | 1.23 - 1.28 | **1.4E-118** |
|  | rs2281135 | 22 | 44332570 | A | 0.17 | Model 2 | 1.27 | 1.24 - 1.30 | **3.3E-94** | 1.18 | 1.12 - 1.24 | **1.4E-10** | 1.3 | 1.22 - 1.38 | **3.5E-17** | 1.26 | 1.23 - 1.28 | **5.8E-117** |
| PNPLA3 | rs2143571 | 22 | 44391686 | A | 0.18 | Model 1 | 1.19 | 1.17 - 1.22 | **2.6E-56** | 1.03 | 0.99 - 1.07 | 0.11 | 1.18 | 1.11 - 1.26 | **2.1E-07** | 1.15 | 1.13 - 1.18 | **8.3E-54** |
|  | rs2143571 | 22 | 44391686 | A | 0.18 | Model 2 | 1.19 | 1.17 - 1.22 | **9.6E-55** | 1.03 | 0.99 - 1.07 | 0.15 | 1.18 | 1.11 - 1.26 | **1.7E-07** | 1.15 | 1.13 - 1.17 | **6.8E-52** |

**ALT2HTN:** ALT >40 for men, >30 for women ≥ 6 months apart over 2 years, no viral hepatitis, and no chronic liver disease + hypertension with ALT≤30 U/L for men and ≤20 U/L for women as controls. **Abbreviations:** rsID: dbSNP identifier (build 151), Chr: chromosome, Pos: basepair position on human genome reference hg19, EA: effect allele, EAF: effect allele frequency among Europeans (Million Veteran Program), OR: odds ratio of risk in cases compared to controls per effect allele (additive model), CI: confidence interval. **Model 1:** adjusted for age, gender, and 10 principal components (PCs), **Model 2:** covariates in Model 1 + alcohol consumption at enrollment measured by the Alcohol Use Disorder Identification Test (AUDIT-C). P-values below 0.006 (adjusted for multiple comparisons) are shown in **bold font**.

**Table S6f.** Previously published NAFLD risk/ALT level-associated variants with genome-wide significance and association with NAFLD phenotype, **ALT2OBESE**

|  |  |  |  |  |  |  | **European Ancestry**  **(n=26,509 cases, 101,701 controls)** | | | **African Ancestry**  **(n=4,775 cases, 23,859 controls)** | | | **Hispanic/Latino Ancestry**  **(n=3,392 cases, 6,514 controls)** | | | **Transethnic Meta-Analysis (n=34,676 cases, 132,074 controls)** | | |
| --- | --- | --- | --- | --- | --- | --- | --- | --- | --- | --- | --- | --- | --- | --- | --- | --- | --- | --- |
| **Gene** | **rsID** | **Chr** | **Pos** | **EA** | **EAF** | **ADJUSTED** | **OR** | **95% CI** | **P** | **OR** | **95% CI** | **P** | **OR** | **95% CI** | **P** | **OR** | **95% CI** | **P** |
| LYPLAL1 | rs12137855 | 1 | 219448378 | C | 0.8 | Model 1 | 1 | 0.98 - 1.03 | 0.71 | 0.97 | 0.92 - 1.02 | 0.26 | 1 | 0.92 - 1.08 | 0.93 | 1 | 0.98 - 1.02 | 0.9 |
|  | rs12137855 | 1 | 219448378 | C | 0.8 | Model 2 | 1 | 0.98 - 1.03 | 0.72 | 0.97 | 0.92 - 1.02 | 0.26 | 1.01 | 0.93 - 1.09 | 0.86 | 1 | 0.98 - 1.02 | 0.94 |
| LYPLAL1 | rs3001032 | 1 | 219727779 | T | 0.69 | Model 1 | 1.04 | 1.02 - 1.07 | **3.5E-05** | 0.97 | 0.93 - 1.01 | 0.2 | 1.04 | 0.98 - 1.10 | 0.24 | 1.03 | 1.01 - 1.05 | **0.00086** |
|  | rs3001032 | 1 | 219727779 | T | 0.69 | Model 2 | 1.04 | 1.02 - 1.07 | **4.5E-05** | 0.97 | 0.93 - 1.01 | 0.2 | 1.04 | 0.98 - 1.10 | 0.22 | 1.03 | 1.01 - 1.05 | **0.001** |
| GCKR | rs780094 | 2 | 27741237 | T | 0.4 | Model 1 | 1.01 | 0.99 - 1.03 | 0.24 | 1.01 | 0.96 - 1.07 | 0.73 | 1.01 | 0.95 - 1.07 | 0.76 | 1.01 | 0.99 - 1.03 | 0.21 |
|  | rs780094 | 2 | 27741237 | T | 0.4 | Model 2 | 1.01 | 0.99 - 1.03 | 0.44 | 1.01 | 0.95 - 1.06 | 0.8 | 1 | 0.94 - 1.07 | 0.94 | 1.01 | 0.99 - 1.02 | 0.42 |
| HSD17B13 | rs6834314 | 4 | 88213808 | A | 0.72 | Model 1 | 1.07 | 1.05 - 1.10 | **1.8E-11** | 1.03 | 0.98 - 1.08 | 0.27 | 1.08 | 0.99 - 1.17 | 0.068 | 1.07 | 1.05 - 1.09 | **7.1E-12** |
|  | rs6834314 | 4 | 88213808 | A | 0.72 | Model 2 | 1.08 | 1.05 - 1.10 | **1.2E-11** | 1.02 | 0.97 - 1.08 | 0.34 | 1.09 | 1.00 - 1.19 | 0.044 | 1.07 | 1.05 - 1.09 | **5.7E-12** |
| HSD17B13 | rs72613567 | 4 | 88231392 | T | 0.73 | Model 1 | 1.08 | 1.05 - 1.10 | **6.0E-12** | 1.06 | 0.98 - 1.16 | 0.13 | 1.11 | 1.02 - 1.21 | 0.018 | 1.08 | 1.06 - 1.10 | **1.4E-13** |
|  | rs72613567 | 4 | 88231392 | T | 0.73 | Model 2 | 1.08 | 1.06 - 1.10 | **6.1E-12** | 1.05 | 0.97 - 1.15 | 0.2 | 1.12 | 1.03 - 1.23 | 0.009 | 1.08 | 1.06 - 1.10 | **1.5E-13** |
| PPP1R3B | rs4240624 | 8 | 9184231 | G | 0.09 | Model 1 | 1.15 | 1.11 - 1.18 | **2.2E-16** | 1.1 | 1.04 - 1.15 | **0.00044** | 1.02 | 0.95 - 1.10 | 0.62 | 1.12 | 1.09 - 1.15 | **2.7E-17** |
|  | rs4240624 | 8 | 9184231 | G | 0.09 | Model 2 | 1.15 | 1.11 - 1.19 | **3.1E-16** | 1.09 | 1.04 - 1.15 | **0.00065** | 1.01 | 0.94 - 1.09 | 0.71 | 1.12 | 1.09 - 1.15 | **8.7E-17** |
| TRIB1 | rs2954021 | 8 | 126482077 | A | 0.5 | Model 1 | 1.13 | 1.10 - 1.15 | **9.6E-35** | 1.02 | 0.97 - 1.06 | 0.44 | 1.11 | 1.04 - 1.17 | **0.0011** | 1.11 | 1.09 - 1.12 | **3.7E-33** |
|  | rs2954021 | 8 | 126482077 | A | 0.5 | Model 2 | 1.13 | 1.10 - 1.15 | **3.2E-34** | 1.02 | 0.98 - 1.06 | 0.4 | 1.1 | 1.04 - 1.17 | **0.0016** | 1.11 | 1.09 - 1.13 | **1.2E-32** |
| ERLIN1 | rs10883437 | 10 | 101795361 | T | 0.61 | Model 1 | 1.07 | 1.05 - 1.10 | **1.8E-13** | 1.05 | 1.01 - 1.09 | 0.026 | 1.05 | 0.99 - 1.12 | 0.1 | 1.07 | 1.05 - 1.09 | **8.6E-15** |
|  | rs10883437 | 10 | 101795361 | T | 0.61 | Model 2 | 1.07 | 1.05 - 1.10 | **5.6E-13** | 1.05 | 1.00 - 1.09 | 0.028 | 1.05 | 0.99 - 1.12 | 0.11 | 1.07 | 1.05 - 1.09 | **2.8E-14** |
| ERLIN1 | rs11597390 | 10 | 101861435 | G | 0.64 | Model 1 | 1.12 | 1.10 - 1.14 | **3.9E-29** | 1.02 | 0.97 - 1.08 | 0.41 | 1.04 | 0.98 - 1.11 | 0.18 | 1.1 | 1.08 - 1.12 | **1.1E-26** |
|  | rs11597390 | 10 | 101861435 | G | 0.64 | Model 2 | 1.12 | 1.10 - 1.14 | **1.1E-28** | 1.02 | 0.97 - 1.07 | 0.43 | 1.04 | 0.98 - 1.11 | 0.19 | 1.1 | 1.08 - 1.12 | **4.1E-26** |
| ERLIN1 | rs11597086 | 10 | 101953705 | A | 0.58 | Model 1 | 1.15 | 1.13 - 1.17 | **3.3E-47** | 1.12 | 1.04 - 1.22 | **0.0029** | 1.11 | 1.04 - 1.19 | **0.0019** | 1.15 | 1.13 - 1.17 | **5.5E-51** |
|  | rs11597086 | 10 | 101953705 | A | 0.58 | Model 2 | 1.15 | 1.13 - 1.17 | **2.7E-46** | 1.12 | 1.04 - 1.21 | **0.0035** | 1.11 | 1.04 - 1.19 | **0.0022** | 1.15 | 1.13 - 1.17 | **6.6E-50** |
| ERLIN1 | rs11591741 | 10 | 101976501 | G | 0.58 | Model 1 | 1.15 | 1.13 - 1.17 | **2.3E-47** | 1.11 | 1.04 - 1.19 | **0.0018** | 1.12 | 1.05 - 1.20 | **0.00087** | 1.15 | 1.13 - 1.17 | **1.3E-51** |
|  | rs11591741 | 10 | 101976501 | G | 0.58 | Model 2 | 1.15 | 1.13 - 1.17 | **2.1E-46** | 1.11 | 1.04 - 1.19 | **0.0019** | 1.12 | 1.05 - 1.20 | **0.001** | 1.15 | 1.13 - 1.17 | **1.5E-50** |
| TM6SF2 | rs2228603 | 19 | 19329924 | T | 0.08 | Model 1 | 1.2 | 1.16 - 1.24 | **4.2E-24** | 0.92 | 0.77 - 1.10 | 0.37 | 1.13 | 0.96 - 1.34 | 0.15 | 1.19 | 1.15 - 1.23 | **7.3E-23** |
|  | rs2228603 | 19 | 19329924 | T | 0.08 | Model 2 | 1.21 | 1.16 - 1.25 | **7.8E-25** | 0.92 | 0.77 - 1.10 | 0.34 | 1.13 | 0.95 - 1.34 | 0.16 | 1.19 | 1.15 - 1.23 | **1.9E-23** |
| TM6SF2 | rs58542926 | 19 | 19379549 | T | 0.07 | Model 1 | 1.25 | 1.21 - 1.30 | **2.0E-36** | 1.05 | 0.94 - 1.17 | 0.43 | 1.19 | 1.04 - 1.37 | 0.014 | 1.23 | 1.19 - 1.27 | **8.1E-36** |
|  | rs58542926 | 19 | 19379549 | T | 0.07 | Model 2 | 1.26 | 1.22 - 1.30 | **4.8E-37** | 1.05 | 0.94 - 1.17 | 0.41 | 1.21 | 1.05 - 1.40 | 0.0077 | 1.24 | 1.20 - 1.28 | **1.2E-36** |
| PNPLA3 | rs738409 | 22 | 44324727 | G | 0.23 | Model 1 | 1.34 | 1.31 - 1.37 | **1.7E-144** | 1.21 | 1.15 - 1.29 | **2.9E-11** | 1.4 | 1.32 - 1.49 | **1.4E-26** | 1.33 | 1.30 - 1.35 | **3.6E-176** |
|  | rs738409 | 22 | 44324727 | G | 0.23 | Model 2 | 1.34 | 1.31 - 1.37 | **1.4E-142** | 1.21 | 1.15 - 1.28 | **4.1E-11** | 1.4 | 1.32 - 1.49 | **1.6E-26** | 1.33 | 1.30 - 1.36 | **5.8E-174** |
| PNPLA3 | rs2281135 | 22 | 44332570 | A | 0.17 | Model 1 | 1.28 | 1.25 - 1.31 | **1.4E-85** | 1.21 | 1.15 - 1.28 | **1.3E-11** | 1.31 | 1.23 - 1.40 | **4.1E-17** | 1.27 | 1.25 - 1.30 | **4.0E-110** |
|  | rs2281135 | 22 | 44332570 | A | 0.17 | Model 2 | 1.28 | 1.25 - 1.31 | **8.1E-84** | 1.21 | 1.14 - 1.28 | **2.1E-11** | 1.31 | 1.23 - 1.40 | **4.5E-17** | 1.27 | 1.25 - 1.30 | **4.9E-108** |
| PNPLA3 | rs2143571 | 22 | 44391686 | A | 0.18 | Model 1 | 1.2 | 1.17 - 1.23 | **8.0E-51** | 1.05 | 1.00 - 1.09 | 0.029 | 1.22 | 1.15 - 1.30 | **1.2E-09** | 1.17 | 1.15 - 1.19 | **5.3E-53** |
|  | rs2143571 | 22 | 44391686 | A | 0.18 | Model 2 | 1.2 | 1.17 - 1.23 | **1.4E-49** | 1.05 | 1.00 - 1.09 | 0.04 | 1.23 | 1.15 - 1.31 | **9.2E-10** | 1.17 | 1.14 - 1.19 | **1.6E-51** |

**ALT2OBESE:** ALT >40 for men, >30 for women ≥ 6 months apart over 2 years, no viral hepatitis, and no chronic liver disease + BMI ≥ 30 kg/m2 with ALT≤30 U/L for men and ≤20 U/L for women as controls. **Abbreviations:** rsID: dbSNP identifier (build 151), Chr: chromosome, Pos: basepair position on human genome reference hg19, EA: effect allele, EAF: effect allele frequency among Europeans (Million Veteran Program), OR: odds ratio of risk in cases compared to controls per effect allele (additive model), CI: confidence interval. **Model 1:** adjusted for age, gender, and 10 principal components (PCs), **Model 2:** covariates in Model 1 + alcohol consumption at enrollment measured by the Alcohol Use Disorder Identification Test (AUDIT-C). P-values below 0.006 (adjusted for multiple comparisons) are shown in **bold font**.

**Table S7a.** Previously published ALT level-associated/ NAFLD risk variants with genome-wide significance and associations with advanced fibrosis defined by **FIB4 score >2.670**

|  |  |  |  |  |  |  | **European Ancestry**  **(n=5,751 cases,**  **40,902 controls)** | | | **African Ancestry**  **(n=657 cases, 7,362 controls)** | | | **Hispanic/Latino Ancestry**  **(n=968**  **cases, 4,902 controls)** | | | **Transethnic Meta-Analysis (n=7,376 cases,**  **53,166 controls)** | | |
| --- | --- | --- | --- | --- | --- | --- | --- | --- | --- | --- | --- | --- | --- | --- | --- | --- | --- | --- |
| **Gene** | **rsID** | **Chr** | **Pos** | **EA** | **EAF** | **ADJUSTED** | **OR** | **95% CI** | **P** | **OR** | **95% CI** | **P** | **OR** | **95% CI** | **P** | **OR** | **95% CI** | **P** |
| LYPLAL1 | rs12137855 | 1 | 219448378 | C | 0.8 | Model 1 | 0.99 | 0.95 - 1.03 | 0.56 | 0.9 | 0.82 - 1.00 | 0.045 | 0.9 | 0.79 - 1.03 | 0.14 | 0.97 | 0.94 - 1.01 | 0.11 |
|  | rs12137855 | 1 | 219448378 | C | 0.8 | Model 2 | 0.99 | 0.95 - 1.02 | 0.47 | 0.91 | 0.82 - 1.00 | 0.057 | 0.9 | 0.79 - 1.03 | 0.13 | 0.97 | 0.94 - 1.00 | 0.093 |
| LYPLAL1 | rs3001032 | 1 | 219727779 | T | 0.69 | Model 1 | 1.01 | 0.98 - 1.05 | 0.38 | 0.97 | 0.90 - 1.04 | 0.4 | 0.94 | 0.85 - 1.04 | 0.24 | 1 | 0.97 - 1.03 | 0.91 |
|  | rs3001032 | 1 | 219727779 | T | 0.69 | Model 2 | 1.01 | 0.98 - 1.05 | 0.4 | 0.96 | 0.89 - 1.04 | 0.35 | 0.94 | 0.85 - 1.04 | 0.23 | 1 | 0.97 - 1.03 | 0.96 |
| GCKR | rs780094 | 2 | 27741237 | T | 0.4 | Model 1 | 1.03 | 1.00 - 1.07 | 0.028 | 1.01 | 0.92 - 1.12 | 0.79 | 1.1 | 1.00 - 1.22 | 0.059 | 1.04 | 1.01 - 1.07 | 0.0092 |
|  | rs780094 | 2 | 27741237 | T | 0.4 | Model 2 | 1.04 | 1.00 - 1.07 | 0.024 | 1.01 | 0.92 - 1.12 | 0.81 | 1.11 | 1.00 - 1.23 | 0.054 | 1.04 | 1.01 - 1.07 | 0.0075 |
| HSD17B13 | rs6834314 | 4 | 88213808 | A | 0.72 | Model 1 | 1.06 | 1.02 - 1.09 | **0.0011** | 0.97 | 0.89 - 1.06 | 0.51 | 1.1 | 0.95 - 1.27 | 0.19 | 1.05 | 1.02 - 1.08 | **0.0023** |
|  | rs6834314 | 4 | 88213808 | A | 0.72 | Model 2 | 1.06 | 1.02 - 1.09 | **0.002** | 0.97 | 0.89 - 1.07 | 0.55 | 1.09 | 0.94 - 1.26 | 0.24 | 1.05 | 1.01 - 1.08 | **0.004** |
| HSD17B13 | rs72613567 | 4 | 88231392 | T | 0.73 | Model 1 | 1.06 | 1.02 - 1.09 | **0.0018** | 1.02 | 0.88 - 1.19 | 0.79 | 1.1 | 0.95 - 1.28 | 0.2 | 1.06 | 1.02 - 1.09 | **0.00097** |
|  | rs72613567 | 4 | 88231392 | T | 0.73 | Model 2 | 1.05 | 1.02 - 1.09 | **0.0027** | 1.03 | 0.88 - 1.20 | 0.74 | 1.09 | 0.94 - 1.27 | 0.26 | 1.05 | 1.02 - 1.09 | 0.0015 |
| PPP1R3B | rs4240624 | 8 | 9184231 | G | 0.09 | Model 1 | 0.97 | 0.92 - 1.02 | 0.27 | 0.95 | 0.86 - 1.04 | 0.27 | 0.94 | 0.83 - 1.07 | 0.34 | 0.96 | 0.92 - 1.01 | 0.084 |
|  | rs4240624 | 8 | 9184231 | G | 0.09 | Model 2 | 0.97 | 0.92 - 1.02 | 0.26 | 0.95 | 0.86 - 1.04 | 0.26 | 0.95 | 0.83 - 1.07 | 0.4 | 0.96 | 0.92 - 1.01 | 0.085 |
| TRIB1 | rs2954021 | 8 | 126482077 | A | 0.5 | Model 1 | 0.99 | 0.96 - 1.02 | 0.33 | 0.97 | 0.90 - 1.05 | 0.43 | 1.11 | 1.00 - 1.23 | 0.042 | 0.99 | 0.97 - 1.02 | 0.55 |
|  | rs2954021 | 8 | 126482077 | A | 0.5 | Model 2 | 0.98 | 0.95 - 1.01 | 0.3 | 0.97 | 0.90 - 1.05 | 0.48 | 1.12 | 1.01 - 1.24 | 0.033 | 0.99 | 0.97 - 1.02 | 0.54 |
| ERLIN1 | rs10883437 | 10 | 101795361 | T | 0.61 | Model 1 | 1.02 | 0.99 - 1.05 | 0.16 | 1 | 0.93 - 1.08 | 1 | 1.09 | 0.98 - 1.20 | 0.097 | 1.02 | 1.00 - 1.05 | 0.089 |
|  | rs10883437 | 10 | 101795361 | T | 0.61 | Model 2 | 1.02 | 0.99 - 1.05 | 0.17 | 1 | 0.93 - 1.08 | 0.98 | 1.1 | 0.99 - 1.21 | 0.068 | 1.02 | 1.00 - 1.05 | 0.085 |
| ERLIN1 | rs11597390 | 10 | 101861435 | G | 0.64 | Model 1 | 1.01 | 0.97 - 1.04 | 0.74 | 0.99 | 0.90 - 1.09 | 0.82 | 1.04 | 0.93 - 1.15 | 0.51 | 1.01 | 0.98 - 1.04 | 0.68 |
|  | rs11597390 | 10 | 101861435 | G | 0.64 | Model 2 | 1 | 0.97 - 1.04 | 0.79 | 0.99 | 0.90 - 1.08 | 0.76 | 1.04 | 0.94 - 1.15 | 0.48 | 1.01 | 0.98 - 1.03 | 0.72 |
| ERLIN1 | rs11597086 | 10 | 101953705 | A | 0.58 | Model 1 | 1 | 0.97 - 1.03 | 0.99 | 1.09 | 0.94 - 1.26 | 0.27 | 1.03 | 0.92 - 1.16 | 0.6 | 1.01 | 0.98 - 1.03 | 0.73 |
|  | rs11597086 | 10 | 101953705 | A | 0.58 | Model 2 | 1 | 0.97 - 1.03 | 0.94 | 1.08 | 0.93 - 1.26 | 0.29 | 1.04 | 0.92 - 1.16 | 0.53 | 1 | 0.98 - 1.03 | 0.76 |
| ERLIN1 | rs11591741 | 10 | 101976501 | G | 0.58 | Model 1 | 1 | 0.97 - 1.03 | 0.97 | 1.05 | 0.92 - 1.19 | 0.49 | 1.02 | 0.91 - 1.15 | 0.68 | 1 | 0.97 - 1.03 | 0.82 |
|  | rs11591741 | 10 | 101976501 | G | 0.58 | Model 2 | 1 | 0.97 - 1.03 | 0.92 | 1.05 | 0.92 - 1.19 | 0.49 | 1.03 | 0.92 - 1.15 | 0.61 | 1 | 0.97 - 1.03 | 0.85 |
| TM6SF2 | rs2228603 | 19 | 19329924 | T | 0.08 | Model 1 | 0.97 | 0.92 - 1.03 | 0.33 | 0.92 | 0.66 - 1.29 | 0.63 | 1.02 | 0.77 - 1.34 | 0.89 | 0.97 | 0.92 - 1.03 | 0.32 |
|  | rs2228603 | 19 | 19329924 | T | 0.08 | Model 2 | 0.97 | 0.92 - 1.02 | 0.26 | 0.92 | 0.66 - 1.29 | 0.64 | 1.03 | 0.78 - 1.36 | 0.84 | 0.97 | 0.92 - 1.02 | 0.26 |
| TM6SF2 | rs58542926 | 19 | 19379549 | T | 0.07 | Model 1 | 1 | 0.95 - 1.06 | 0.92 | 0.97 | 0.79 - 1.19 | 0.79 | 1.22 | 0.99 - 1.52 | 0.064 | 1.01 | 0.96 - 1.06 | 0.64 |
|  | rs58542926 | 19 | 19379549 | T | 0.07 | Model 2 | 1 | 0.94 - 1.05 | 0.87 | 0.97 | 0.79 - 1.19 | 0.78 | 1.23 | 0.99 - 1.53 | 0.062 | 1.01 | 0.96 - 1.06 | 0.83 |
| PNPLA3 | rs738409 | 22 | 44324727 | G | 0.23 | Model 1 | 1.1 | 1.06 - 1.14 | **2.8E-08** | 0.95 | 0.85 - 1.05 | 0.31 | 1.07 | 0.97 - 1.19 | 0.16 | 1.08 | 1.05 - 1.12 | **2.4E-07** |
|  | rs738409 | 22 | 44324727 | G | 0.23 | Model 2 | 1.09 | 1.06 - 1.13 | **2.2E-07** | 0.94 | 0.84 - 1.04 | 0.22 | 1.07 | 0.97 - 1.19 | 0.17 | 1.08 | 1.04 - 1.11 | **2.1E-06** |
| PNPLA3 | rs2281135 | 22 | 44332570 | A | 0.17 | Model 1 | 1.1 | 1.06 - 1.14 | **1.4E-06** | 0.97 | 0.88 - 1.08 | 0.58 | 1.04 | 0.94 - 1.15 | 0.47 | 1.08 | 1.04 - 1.11 | **1.4E-05** |
|  | rs2281135 | 22 | 44332570 | A | 0.17 | Model 2 | 1.09 | 1.05 - 1.13 | **1.2E-05** | 0.97 | 0.87 - 1.07 | 0.5 | 1.03 | 0.93 - 1.14 | 0.54 | 1.07 | 1.03 - 1.10 | **0.00011** |
| PNPLA3 | rs2143571 | 22 | 44391686 | A | 0.18 | Model 1 | 1.07 | 1.03 - 1.11 | **0.00025** | 0.98 | 0.90 - 1.06 | 0.56 | 1.05 | 0.95 - 1.17 | 0.35 | 1.05 | 1.02 - 1.09 | **0.0013** |
|  | rs2143571 | 22 | 44391686 | A | 0.18 | Model 2 | 1.07 | 1.03 - 1.11 | **0.0004** | 0.97 | 0.90 - 1.05 | 0.47 | 1.06 | 0.95 - 1.18 | 0.31 | 1.05 | 1.02 - 1.09 | **0.0022** |

**FIB4 score**: Age [years] x AST [U/L] / (platelets [10^9/L] x sqrt (ALT)). **Abbreviations:** rsID: dbSNP identifier (build 151), Chr: chromosome, Pos: basepair position on human genome reference hg19, EA: effect allele, EAF: effect allele frequency among Europeans (Million Veteran Program), OR: odds ratio of risk in cases compared to controls per effect allele (additive model), CI: confidence interval. **Model 1:** adjusted for age, gender, and 10 principal components (PCs), **Model 2:** covariates in Model 1 + alcohol consumption at enrollment measured by the Alcohol Use Disorder Identification Test (AUDIT-C). P-values below 0.006 (adjusted for multiple comparisons) are shown in **bold font**.

**Table S7b.** Previously published ALT level-associated/ NAFLD risk variants with genome-wide significance and associations with advanced fibrosis defined by **NAFLD fibrosis score ≥0.676**

|  |  |  |  |  |  |  | **European Ancestry**  **(n=15,874 cases,**  **30,779 controls)** | | | **African Ancestry**  **(n=2,308 cases, 5,711 controls)** | | | **Hispanic/Latino Ancestry**  **(n=1,571 cases, 4,299 controls)** | | | **Transethnic Meta-Analysis (n=19,764 cases,**  **40,778 controls)** | | |
| --- | --- | --- | --- | --- | --- | --- | --- | --- | --- | --- | --- | --- | --- | --- | --- | --- | --- | --- |
| **Gene** | **rsID** | **Chr** | **Pos** | **EA** | **EAF** | **Adjusted** | **OR** | **95% CI** | **P** | **OR** | **95% CI** | **P** | **OR** | **95% CI** | **P** | **OR** | **95% CI** | **P** |
| LYPLAL1 | rs12137855 | 1 | 219448378 | C | 0.8 | Model 1 | 0.98 | 0.95 - 1.02 | 0.31 | 1 | 0.91 - 1.09 | 0.92 | 0.91 | 0.80 - 1.02 | 0.11 | 0.98 | 0.95 - 1.01 | 0.18 |
|  | rs12137855 | 1 | 219448378 | C | 0.8 | Model 2 | 0.98 | 0.95 - 1.02 | 0.31 | 1 | 0.91 - 1.09 | 0.93 | 0.92 | 0.81 - 1.03 | 0.16 | 0.98 | 0.95 - 1.01 | 0.19 |
| LYPLAL1 | rs3001032 | 1 | 219727779 | T | 0.69 | Model 1 | 0.99 | 0.96 - 1.02 | 0.33 | 0.95 | 0.89 - 1.02 | 0.16 | 0.95 | 0.87 - 1.03 | 0.23 | 0.98 | 0.95 - 1.00 | 0.082 |
|  | rs3001032 | 1 | 219727779 | T | 0.69 | Model 2 | 0.99 | 0.96 - 1.02 | 0.47 | 0.96 | 0.90 - 1.02 | 0.21 | 0.95 | 0.87 - 1.03 | 0.22 | 0.98 | 0.96 - 1.01 | 0.14 |
| GCKR | rs780094 | 2 | 27741237 | T | 0.4 | Model 1 | 1 | 0.98 - 1.03 | 0.76 | 0.91 | 0.84 - 0.99 | 0.035 | 1.01 | 0.93 - 1.11 | 0.77 | 1 | 0.97 - 1.02 | 0.8 |
|  | rs780094 | 2 | 27741237 | T | 0.4 | Model 2 | 1 | 0.97 - 1.03 | 0.93 | 0.91 | 0.84 - 1.00 | 0.041 | 1.02 | 0.93 - 1.12 | 0.64 | 0.99 | 0.97 - 1.02 | 0.58 |
| HSD17B13 | rs6834314 | 4 | 88213808 | A | 0.72 | Model 1 | 1.02 | 0.99 - 1.05 | 0.31 | 0.95 | 0.88 - 1.03 | 0.21 | 1.07 | 0.95 - 1.22 | 0.26 | 1.01 | 0.98 - 1.04 | 0.47 |
|  | rs6834314 | 4 | 88213808 | A | 0.72 | Model 2 | 1.02 | 0.99 - 1.05 | 0.17 | 0.95 | 0.88 - 1.03 | 0.2 | 1.07 | 0.94 - 1.21 | 0.32 | 1.01 | 0.99 - 1.04 | 0.32 |
| HSD17B13 | rs72613567 | 4 | 88231392 | T | 0.73 | Model 1 | 1.01 | 0.98 - 1.05 | 0.37 | 1.02 | 0.90 - 1.16 | 0.74 | 1.07 | 0.94 - 1.22 | 0.29 | 1.02 | 0.99 - 1.05 | 0.25 |
|  | rs72613567 | 4 | 88231392 | T | 0.73 | Model 2 | 1.02 | 0.99 - 1.05 | 0.2 | 1.02 | 0.89 - 1.16 | 0.79 | 1.06 | 0.93 - 1.21 | 0.37 | 1.02 | 0.99 - 1.05 | 0.14 |
| PPP1R3B | rs4240624 | 8 | 9184231 | G | 0.09 | Model 1 | 1.02 | 0.97 - 1.07 | 0.44 | 0.98 | 0.90 - 1.07 | 0.67 | 0.91 | 0.81 - 1.01 | 0.088 | 1 | 0.96 - 1.04 | 0.89 |
|  | rs4240624 | 8 | 9184231 | G | 0.09 | Model 2 | 1.02 | 0.97 - 1.07 | 0.47 | 0.99 | 0.91 - 1.07 | 0.8 | 0.91 | 0.82 - 1.02 | 0.11 | 1 | 0.96 - 1.04 | 0.95 |
| TRIB1 | rs2954021 | 8 | 126482077 | A | 0.5 | Model 1 | 0.98 | 0.95 - 1.01 | 0.15 | 0.93 | 0.87 - 1.00 | 0.049 | 1.15 | 1.05 - 1.25 | **0.0023** | 0.99 | 0.96 - 1.01 | 0.25 |
|  | rs2954021 | 8 | 126482077 | A | 0.5 | Model 2 | 0.98 | 0.95 - 1.00 | 0.078 | 0.94 | 0.88 - 1.01 | 0.082 | 1.14 | 1.04 - 1.25 | **0.0035** | 0.98 | 0.96 - 1.01 | 0.16 |
| ERLIN1 | rs10883437 | 10 | 101795361 | T | 0.61 | Model 1 | 1.02 | 0.99 - 1.04 | 0.28 | 0.98 | 0.92 - 1.05 | 0.64 | 1.01 | 0.93 - 1.10 | 0.8 | 1.01 | 0.99 - 1.04 | 0.4 |
|  | rs10883437 | 10 | 101795361 | T | 0.61 | Model 2 | 1.02 | 0.99 - 1.05 | 0.25 | 0.99 | 0.93 - 1.06 | 0.73 | 1.01 | 0.93 - 1.11 | 0.76 | 1.01 | 0.99 - 1.04 | 0.34 |
| ERLIN1 | rs11597390 | 10 | 101861435 | G | 0.64 | Model 1 | 1 | 0.97 - 1.03 | 0.91 | 0.99 | 0.91 - 1.07 | 0.79 | 0.97 | 0.89 - 1.07 | 0.56 | 1 | 0.97 - 1.02 | 0.72 |
|  | rs11597390 | 10 | 101861435 | G | 0.64 | Model 2 | 1 | 0.97 - 1.03 | 0.89 | 1 | 0.92 - 1.08 | 0.93 | 0.98 | 0.89 - 1.07 | 0.61 | 1 | 0.97 - 1.02 | 0.76 |
| ERLIN1 | rs11597086 | 10 | 101953705 | A | 0.58 | Model 1 | 1 | 0.97 - 1.03 | 0.96 | 1.02 | 0.90 - 1.16 | 0.73 | 0.98 | 0.89 - 1.08 | 0.71 | 1 | 0.97 - 1.03 | 0.95 |
|  | rs11597086 | 10 | 101953705 | A | 0.58 | Model 2 | 1 | 0.97 - 1.03 | 0.86 | 1.02 | 0.90 - 1.16 | 0.71 | 0.98 | 0.89 - 1.09 | 0.73 | 1 | 0.97 - 1.02 | 0.86 |
| ERLIN1 | rs11591741 | 10 | 101976501 | G | 0.58 | Model 1 | 1 | 0.97 - 1.03 | 0.95 | 0.99 | 0.89 - 1.11 | 0.9 | 0.99 | 0.89 - 1.09 | 0.77 | 1 | 0.97 - 1.02 | 0.87 |
|  | rs11591741 | 10 | 101976501 | G | 0.58 | Model 2 | 1 | 0.97 - 1.03 | 0.85 | 1 | 0.89 - 1.11 | 0.95 | 0.99 | 0.89 - 1.09 | 0.78 | 1 | 0.97 - 1.02 | 0.79 |
| TM6SF2 | rs2228603 | 19 | 19329924 | T | 0.08 | Model 1 | 0.97 | 0.92 - 1.02 | 0.22 | 0.99 | 0.75 - 1.32 | 0.97 | 1 | 0.78 - 1.28 | 1 | 0.97 | 0.93 - 1.02 | 0.24 |
|  | rs2228603 | 19 | 19329924 | T | 0.08 | Model 2 | 0.96 | 0.92 - 1.01 | 0.15 | 0.98 | 0.74 - 1.30 | 0.9 | 1.01 | 0.79 - 1.29 | 0.93 | 0.97 | 0.92 - 1.01 | 0.17 |
| TM6SF2 | rs58542926 | 19 | 19379549 | T | 0.07 | Model 1 | 0.97 | 0.93 - 1.02 | 0.25 | 0.88 | 0.74 - 1.06 | 0.18 | 1.04 | 0.85 - 1.27 | 0.71 | 0.97 | 0.93 - 1.01 | 0.18 |
|  | rs58542926 | 19 | 19379549 | T | 0.07 | Model 2 | 0.97 | 0.92 - 1.02 | 0.21 | 0.88 | 0.74 - 1.06 | 0.18 | 1.03 | 0.84 - 1.26 | 0.78 | 0.97 | 0.92 - 1.01 | 0.14 |
| PNPLA3 | rs738409 | 22 | 44324727 | G | 0.23 | Model 1 | 1.07 | 1.03 - 1.10 | **3.8E-05** | 0.99 | 0.91 - 1.08 | 0.85 | 1.09 | 1.00 - 1.19 | 0.045 | 1.06 | 1.03 - 1.09 | **2.0E-05** |
|  | rs738409 | 22 | 44324727 | G | 0.23 | Model 2 | 1.07 | 1.03 - 1.10 | **4.6E-05** | 0.98 | 0.90 - 1.08 | 0.74 | 1.08 | 0.99 - 1.18 | 0.075 | 1.06 | 1.03 - 1.09 | **4.0E-05** |
| PNPLA3 | rs2281135 | 22 | 44332570 | A | 0.17 | Model 1 | 1.06 | 1.02 - 1.10 | **0.0008** | 1.01 | 0.92 - 1.10 | 0.89 | 1.06 | 0.97 - 1.16 | 0.23 | 1.05 | 1.02 - 1.09 | **0.0007** |
|  | rs2281135 | 22 | 44332570 | A | 0.17 | Model 2 | 1.06 | 1.02 - 1.10 | **0.0011** | 1 | 0.92 - 1.09 | 0.98 | 1.04 | 0.95 - 1.14 | 0.4 | 1.05 | 1.02 - 1.08 | **0.0016** |
| PNPLA3 | rs2143571 | 22 | 44391686 | A | 0.18 | Model 1 | 1.05 | 1.02 - 1.09 | **0.0045** | 0.99 | 0.93 - 1.06 | 0.78 | 1.09 | 0.99 - 1.20 | 0.066 | 1.04 | 1.01 - 1.07 | **0.0042** |
|  | rs2143571 | 22 | 44391686 | A | 0.18 | Model 2 | 1.05 | 1.02 - 1.09 | **0.0039** | 0.98 | 0.92 - 1.05 | 0.65 | 1.09 | 0.99 - 1.20 | 0.069 | 1.04 | 1.01 - 1.07 | **0.0049** |

**NAFFIB:** NAFLD fibrosis score = -1.675+(0.037*age)+(0.094*BMI)+(1.13*(diabetes or prediabetes))+(0.99*(AST/ALT))-(0.013*platelets)-(0.66*albumin)**. Abbreviations:** rsID: dbSNP identifier (build 151), Chr: chromosome, Pos: basepair position on human genome reference hg19, EA: effect allele, EAF: effect allele frequency among Europeans (Million Veteran Program), OR: odds ratio of risk in cases compared to controls per effect allele (additive model), CI: confidence interval. **Model 1:** adjusted for age, gender, and 10 principal components (PCs), **Model 2:** covariates in Model 1 + alcohol consumption at enrollment measured by the Alcohol Use Disorder Identification Test (AUDIT-C). P-values below 0.006 (adjusted for multiple comparisons) are shown in **bold font**.

**Table S7c.** Previously published ALT level-associated/ NAFLD risk variants with genome-wide significance and associations with **platelet count** at enrollment as a continuous measure

|  |  |  |  |  |  |  | **European Ancestry**  **(n=46,653)** | | | **African Ancestry**  **(n=8,019)** | | | **Hispanic/Latino Ancestry**  **(n=5,870)** | | | **Trans-ethnic Meta-Analysis**  **(n=60,542)** | | |
| --- | --- | --- | --- | --- | --- | --- | --- | --- | --- | --- | --- | --- | --- | --- | --- | --- | --- | --- |
| **Gene** | **rsID** | **Chr** | **Pos** | **EA** | **EAF** | **ADJUSTED** | **BETA** | **SE** | **P** | **BETA** | **SE** | **P** | **BETA** | **SE** | **P** | **BETA** | **SE** | **P** |
| LYPLAL1 | rs12137855 | 1 | 219448378 | C | 0.8 | Model 1 | 0.2145 | 0.2442 | 0.38 | 0.161 | 0.5165 | 0.76 | 0.3441 | 0.842 | 0.68 | 0.2137 | 0.2135 | 0.32 |
|  | rs12137855 | 1 | 219448378 | C | 0.8 | Model 2 | 0.219 | 0.2442 | 0.37 | 0.2068 | 0.5132 | 0.69 | 0.1881 | 0.8374 | 0.82 | 0.2149 | 0.2132 | 0.31 |
| LYPLAL1 | rs3001032 | 1 | 219727779 | T | 0.69 | Model 1 | -0.4421 | 0.2128 | 0.038 | -0.0158 | 0.3881 | 0.97 | 0.1941 | 0.6094 | 0.75 | -0.2975 | 0.1784 | 0.095 |
|  | rs3001032 | 1 | 219727779 | T | 0.69 | Model 2 | -0.41 | 0.2128 | 0.054 | -0.1049 | 0.3856 | 0.79 | -0.1339 | 0.6054 | 0.82 | -0.321 | 0.1781 | 0.071 |
| GCKR | rs780094 | 2 | 27741237 | T | 0.4 | Model 1 | 1.5664 | 0.1999 | **4.7E-15** | 2.2184 | 0.512 | **1.5E-05** | 1.0627 | 0.6316 | 0.092 | 1.6055 | 0.1786 | **2.5E-19** |
|  | rs780094 | 2 | 27741237 | T | 0.4 | Model 2 | 1.7189 | 0.1999 | **8.2E-18** | 2.0912 | 0.5087 | **3.9E-05** | 0.987 | 0.6274 | 0.12 | 1.7055 | 0.1784 | **1.2E-21** |
| HSD17B13 | rs6834314 | 4 | 88213808 | A | 0.72 | Model 1 | -1.603 | 0.2191 | **2.5E-13** | -0.7869 | 0.465 | 0.091 | -2.3503 | 0.8252 | **0.0044** | -1.5036 | 0.1927 | **6.1E-15** |
|  | rs6834314 | 4 | 88213808 | A | 0.72 | Model 2 | -1.6386 | 0.219 | **7.4E-14** | -0.6836 | 0.4617 | 0.14 | -2.3978 | 0.8204 | **0.0035** | -1.5145 | 0.1924 | **3.5E-15** |
| HSD17B13 | rs72613567 | 4 | 88231392 | T | 0.73 | Model 1 | -1.6399 | 0.2204 | **1.0E-13** | -1.7879 | 0.7601 | 0.019 | -2.3007 | 0.8656 | 0.0079 | -1.688 | 0.2056 | **2.2E-16** |
|  | rs72613567 | 4 | 88231392 | T | 0.73 | Model 2 | -1.6915 | 0.2203 | **1.6E-14** | -1.6314 | 0.7554 | 0.031 | -2.3482 | 0.8614 | 0.0064 | -1.7244 | 0.2054 | **4.7E-17** |
| PPP1R3B | rs4240624 | 8 | 9184231 | G | 0.09 | Model 1 | -0.2038 | 0.3456 | 0.56 | -0.7284 | 0.4923 | 0.14 | -0.3647 | 0.7734 | 0.64 | -0.3755 | 0.2657 | 0.16 |
|  | rs4240624 | 8 | 9184231 | G | 0.09 | Model 2 | -0.156 | 0.3457 | 0.65 | -0.7825 | 0.489 | 0.11 | -0.5125 | 0.7684 | 0.5 | -0.3823 | 0.265 | 0.15 |
| TRIB1 | rs2954021 | 8 | 126482077 | A | 0.5 | Model 1 | -0.1926 | 0.1965 | 0.33 | -0.0697 | 0.3995 | 0.86 | -1.322 | 0.6057 | 0.029 | -0.2588 | 0.1693 | 0.13 |
|  | rs2954021 | 8 | 126482077 | A | 0.5 | Model 2 | -0.2137 | 0.1964 | 0.28 | 0.0318 | 0.397 | 0.94 | -1.1697 | 0.6018 | 0.052 | -0.2446 | 0.169 | 0.15 |
| ERLIN1 | rs10883437 | 10 | 101795361 | T | 0.61 | Model 1 | -0.179 | 0.2007 | 0.37 | -0.3814 | 0.39 | 0.33 | 0.2563 | 0.6031 | 0.67 | -0.1829 | 0.1711 | 0.29 |
|  | rs10883437 | 10 | 101795361 | T | 0.61 | Model 2 | -0.1783 | 0.2006 | 0.37 | -0.4966 | 0.3875 | 0.2 | 0.2349 | 0.5994 | 0.7 | -0.2066 | 0.1708 | 0.23 |
| ERLIN1 | rs11597390 | 10 | 101861435 | G | 0.64 | Model 1 | 0.0682 | 0.2038 | 0.74 | 0.0191 | 0.4857 | 0.97 | 1.2927 | 0.6232 | 0.038 | 0.1635 | 0.1799 | 0.36 |
|  | rs11597390 | 10 | 101861435 | G | 0.64 | Model 2 | 0.029 | 0.2037 | 0.89 | 0.2389 | 0.4826 | 0.62 | 1.3024 | 0.619 | 0.035 | 0.1652 | 0.1796 | 0.36 |
| ERLIN1 | rs11597086 | 10 | 101953705 | A | 0.58 | Model 1 | -0.1091 | 0.1986 | 0.58 | -0.3789 | 0.7158 | 0.6 | 0.6873 | 0.6765 | 0.31 | -0.068 | 0.1842 | 0.71 |
|  | rs11597086 | 10 | 101953705 | A | 0.58 | Model 2 | -0.1867 | 0.1985 | 0.35 | -0.1659 | 0.7115 | 0.82 | 0.5758 | 0.6727 | 0.39 | -0.1283 | 0.1839 | 0.49 |
| ERLIN1 | rs11591741 | 10 | 101976501 | G | 0.58 | Model 1 | -0.0896 | 0.1986 | 0.65 | -0.5028 | 0.6256 | 0.42 | 0.7409 | 0.6751 | 0.27 | -0.0641 | 0.1822 | 0.72 |
|  | rs11591741 | 10 | 101976501 | G | 0.58 | Model 2 | -0.1733 | 0.1985 | 0.38 | -0.4457 | 0.6219 | 0.47 | 0.6319 | 0.6714 | 0.35 | -0.1374 | 0.182 | 0.45 |
| TM6SF2 | rs2228603 | 19 | 19329924 | T | 0.08 | Model 1 | 0.0125 | 0.3742 | 0.97 | 1.6201 | 1.6373 | 0.32 | 1.5518 | 1.7312 | 0.37 | 0.1544 | 0.3569 | 0.67 |
|  | rs2228603 | 19 | 19329924 | T | 0.08 | Model 2 | -0.011 | 0.374 | 0.98 | 1.4569 | 1.6275 | 0.37 | 1.903 | 1.7193 | 0.27 | 0.1418 | 0.3566 | 0.69 |
| TM6SF2 | rs58542926 | 19 | 19379549 | T | 0.07 | Model 1 | -0.1414 | 0.3733 | 0.7 | 0.8529 | 1.0531 | 0.42 | -0.2075 | 1.3768 | 0.88 | -0.0412 | 0.3409 | 0.9 |
|  | rs58542926 | 19 | 19379549 | T | 0.07 | Model 2 | -0.1609 | 0.373 | 0.67 | 0.5054 | 1.0468 | 0.63 | -0.0508 | 1.369 | 0.97 | -0.0836 | 0.3404 | 0.81 |
| PNPLA3 | rs738409 | 22 | 44324727 | G | 0.23 | Model 1 | -2.7598 | 0.2352 | **8.6E-32** | -2.603 | 0.5555 | **2.8E-06** | -4.0482 | 0.6159 | **5.0E-11** | -2.8804 | 0.2043 | **3.9E-45** |
|  | rs738409 | 22 | 44324727 | G | 0.23 | Model 2 | -2.8032 | 0.2352 | **1.0E-32** | -2.6006 | 0.5516 | **2.4E-06** | -4.0759 | 0.6114 | **2.7E-11** | -2.9172 | 0.204 | **2.2E-46** |
| PNPLA3 | rs2281135 | 22 | 44332570 | A | 0.17 | Model 1 | -2.2443 | 0.2624 | **1.2E-17** | -1.9194 | 0.5419 | **0.0004** | -3.8226 | 0.6346 | **1.7E-09** | -2.3821 | 0.2213 | **5.1E-27** |
|  | rs2281135 | 22 | 44332570 | A | 0.17 | Model 2 | -2.2865 | 0.2623 | **2.8E-18** | -1.9559 | 0.538 | **0.00028** | -3.9023 | 0.6301 | **6.0E-10** | -2.4292 | 0.2208 | **3.7E-28** |
| PNPLA3 | rs2143571 | 22 | 44391686 | A | 0.18 | Model 1 | -1.5541 | 0.2555 | **1.2E-09** | -0.7543 | 0.4085 | 0.065 | -2.431 | 0.6569 | **0.00022** | -1.4372 | 0.2057 | **2.8E-12** |
|  | rs2143571 | 22 | 44391686 | A | 0.18 | Model 2 | -1.5704 | 0.2555 | **8.0E-10** | -0.8056 | 0.4057 | 0.047 | -2.2736 | 0.6529 | **0.0005** | -1.4441 | 0.2053 | **2.0E-12** |

**Abbreviations:** rsID: dbSNP identifier (build 151), Chr: chromosome, Pos: basepair position on human genome reference hg19, EA: effect allele, EAF: effect allele frequency among Europeans (Million Veteran Program), OR: odds ratio of risk in cases compared to controls per effect allele (additive model), CI: confidence interval. **Model 1:** adjusted for age, gender, and 10 principal components (PCs), **Model 2:** covariates in Model 1 + alcohol consumption at enrollment measured by the Alcohol Use Disorder Identification Test (AUDIT-C). P-values below 0.006 (adjusted for multiple comparisons) are shown in **bold font**.**Table S7d.** Previously published ALT level-associated/ NAFLD risk variants with genome-wide significance and associations with the **FIB4** score as a continuous measure

|  |  |  |  |  |  |  | **European Ancestry**  **(n=46,653)** | | | **African Ancestry**  **(n=8,019)** | | | **Hispanic/Latino Ancestry**  **(n=5,870)** | | | **Trans-ethnic Meta-Analysis**  **(n=60,542)** | | |
| --- | --- | --- | --- | --- | --- | --- | --- | --- | --- | --- | --- | --- | --- | --- | --- | --- | --- | --- |
| **Gene** | **rsID** | **Chr** | **Pos** | **EA** | **EAF** | **ADJUSTED** | **BETA** | **SE** | **P** | **BETA** | **SE** | **P** | **BETA** | **SE** | **P** | **BETA** | **SE** | **P** |
| LYPLAL1 | rs12137855 | 1 | 219448378 | C | 0.8 | Model 1 | -0.009 | 0.033 | 0.79 | -0.1148 | 0.0567 | 0.043 | -0.1338 | 0.1045 | 0.2 | -0.0426 | 0.0275 | 0.12 |
|  | rs12137855 | 1 | 219448378 | C | 0.8 | Model 2 | -0.0124 | 0.0338 | 0.71 | -0.1166 | 0.057 | 0.041 | -0.1404 | 0.1059 | 0.18 | -0.0466 | 0.028 | 0.097 |
|  | rs12137855 | 1 | 219448378 | C | 0.8 | Model 3 | -0.0138 | 0.034 | 0.68 | -0.1182 | 0.0572 | 0.039 | -0.142 | 0.1063 | 0.18 | -0.0481 | 0.0282 | 0.088 |
| LYPLAL1 | rs3001032 | 1 | 219727779 | T | 0.69 | Model 1 | 0.0407 | 0.0289 | 0.16 | -0.1036 | 0.0431 | 0.016 | -0.1029 | 0.0764 | 0.18 | -0.013 | 0.0229 | 0.57 |
|  | rs3001032 | 1 | 219727779 | T | 0.69 | Model 2 | 0.0447 | 0.0296 | 0.13 | -0.1062 | 0.0433 | 0.014 | -0.1027 | 0.0772 | 0.18 | -0.0124 | 0.0233 | 0.6 |
|  | rs3001032 | 1 | 219727779 | T | 0.69 | Model 3 | 0.0477 | 0.0297 | 0.11 | -0.1044 | 0.0435 | 0.016 | -0.0982 | 0.0775 | 0.21 | -0.0097 | 0.0234 | 0.68 |
| GCKR | rs780094 | 2 | 27741237 | T | 0.4 | Model 1 | 0.067 | 0.0269 | 0.013 | 0.0265 | 0.0563 | 0.64 | 0.0803 | 0.0786 | 0.31 | 0.0612 | 0.0232 | 0.0083 |
|  | rs780094 | 2 | 27741237 | T | 0.4 | Model 2 | 0.0677 | 0.0276 | 0.014 | 0.0257 | 0.0566 | 0.65 | 0.0846 | 0.0794 | 0.29 | 0.0619 | 0.0237 | 0.0089 |
|  | rs780094 | 2 | 27741237 | T | 0.4 | Model 3 | 0.0771 | 0.0277 | **0.0054** | 0.026 | 0.0569 | 0.65 | 0.098 | 0.0799 | 0.22 | 0.07 | 0.0238 | **0.0032** |
| HSD17B13 | rs6834314 | 4 | 88213808 | A | 0.72 | Model 1 | 0.0811 | 0.0298 | 0.0065 | -0.0485 | 0.0518 | 0.35 | 0.1684 | 0.1056 | 0.11 | 0.0557 | 0.0251 | 0.027 |
|  | rs6834314 | 4 | 88213808 | A | 0.72 | Model 2 | 0.0752 | 0.0305 | 0.014 | -0.0462 | 0.0521 | 0.38 | 0.1623 | 0.1068 | 0.13 | 0.051 | 0.0256 | 0.046 |
|  | rs6834314 | 4 | 88213808 | A | 0.72 | Model 3 | 0.0721 | 0.0307 | 0.019 | -0.0463 | 0.0523 | 0.38 | 0.1638 | 0.1071 | 0.13 | 0.0488 | 0.0257 | 0.057 |
| HSD17B13 | rs72613567 | 4 | 88231392 | T | 0.73 | Model 1 | 0.0783 | 0.03 | 0.0091 | 0.0972 | 0.0861 | 0.26 | 0.1516 | 0.1113 | 0.17 | 0.0847 | 0.0275 | **0.002** |
|  | rs72613567 | 4 | 88231392 | T | 0.73 | Model 2 | 0.0731 | 0.0307 | 0.017 | 0.1 | 0.0867 | 0.25 | 0.1442 | 0.1127 | 0.2 | 0.0803 | 0.028 | **0.0042** |
|  | rs72613567 | 4 | 88231392 | T | 0.73 | Model 3 | 0.0704 | 0.0309 | 0.023 | 0.0989 | 0.087 | 0.26 | 0.1476 | 0.1131 | 0.19 | 0.0782 | 0.0282 | **0.0055** |
| PPP1R3B | rs4240624 | 8 | 9184231 | G | 0.09 | Model 1 | -0.0518 | 0.0449 | 0.25 | -0.0673 | 0.0539 | 0.21 | -0.053 | 0.0952 | 0.58 | -0.0576 | 0.0324 | 0.076 |
|  | rs4240624 | 8 | 9184231 | G | 0.09 | Model 2 | -0.0499 | 0.046 | 0.28 | -0.0672 | 0.0543 | 0.22 | -0.0399 | 0.0962 | 0.68 | -0.0551 | 0.033 | 0.094 |
|  | rs4240624 | 8 | 9184231 | G | 0.09 | Model 3 | -0.0493 | 0.0462 | 0.29 | -0.0678 | 0.0544 | 0.21 | -0.0394 | 0.0967 | 0.68 | -0.055 | 0.0331 | 0.097 |
| TRIB1 | rs2954021 | 8 | 126482077 | A | 0.5 | Model 1 | 0.0185 | 0.0264 | 0.48 | 0.0025 | 0.044 | 0.95 | 0.0457 | 0.0763 | 0.55 | 0.0168 | 0.0217 | 0.44 |
|  | rs2954021 | 8 | 126482077 | A | 0.5 | Model 2 | 0.0143 | 0.0271 | 0.6 | 0.0063 | 0.0443 | 0.89 | 0.0529 | 0.0772 | 0.49 | 0.0155 | 0.0221 | 0.48 |
|  | rs2954021 | 8 | 126482077 | A | 0.5 | Model 3 | 0.0226 | 0.0272 | 0.41 | 0.0052 | 0.0444 | 0.91 | 0.0575 | 0.0776 | 0.46 | 0.0211 | 0.0222 | 0.34 |
| ERLIN1 | rs10883437 | 10 | 101795361 | T | 0.61 | Model 1 | 0.0246 | 0.0273 | 0.37 | -0.0229 | 0.0428 | 0.59 | 0.0871 | 0.0751 | 0.25 | 0.0174 | 0.022 | 0.43 |
|  | rs10883437 | 10 | 101795361 | T | 0.61 | Model 2 | 0.0243 | 0.028 | 0.39 | -0.0219 | 0.043 | 0.61 | 0.0977 | 0.0759 | 0.2 | 0.0181 | 0.0224 | 0.42 |
|  | rs10883437 | 10 | 101795361 | T | 0.61 | Model 3 | 0.0232 | 0.0281 | 0.41 | -0.022 | 0.0432 | 0.61 | 0.0976 | 0.0763 | 0.2 | 0.0174 | 0.0225 | 0.44 |
| ERLIN1 | rs11597390 | 10 | 101861435 | G | 0.64 | Model 1 | -0.0008 | 0.028 | 0.98 | -0.0289 | 0.054 | 0.59 | 0.1245 | 0.0783 | 0.11 | 0.0052 | 0.0237 | 0.82 |
|  | rs11597390 | 10 | 101861435 | G | 0.64 | Model 2 | -0.0034 | 0.0286 | 0.91 | -0.0334 | 0.0543 | 0.54 | 0.1309 | 0.0791 | 0.098 | 0.0032 | 0.0241 | 0.9 |
|  | rs11597390 | 10 | 101861435 | G | 0.64 | Model 3 | -0.0036 | 0.0288 | 0.9 | -0.0299 | 0.0545 | 0.58 | 0.1325 | 0.0796 | 0.096 | 0.0038 | 0.0243 | 0.88 |
| ERLIN1 | rs11597086 | 10 | 101953705 | A | 0.58 | Model 1 | 0.0035 | 0.0272 | 0.9 | -0.0647 | 0.0826 | 0.43 | 0.1839 | 0.0862 | 0.033 | 0.0122 | 0.0248 | 0.62 |
|  | rs11597086 | 10 | 101953705 | A | 0.58 | Model 2 | 0.0008 | 0.0279 | 0.98 | -0.065 | 0.083 | 0.43 | 0.1924 | 0.087 | 0.027 | 0.0108 | 0.0253 | 0.67 |
|  | rs11597086 | 10 | 101953705 | A | 0.58 | Model 3 | 0.0013 | 0.028 | 0.96 | -0.0614 | 0.0833 | 0.46 | 0.1918 | 0.0875 | 0.028 | 0.0115 | 0.0254 | 0.65 |
| ERLIN1 | rs11591741 | 10 | 101976501 | G | 0.58 | Model 1 | 0.0014 | 0.0272 | 0.96 | -0.1093 | 0.0714 | 0.13 | 0.1791 | 0.0862 | 0.038 | 0.0027 | 0.0244 | 0.91 |
|  | rs11591741 | 10 | 101976501 | G | 0.58 | Model 2 | -0.0013 | 0.0279 | 0.96 | -0.1108 | 0.0718 | 0.12 | 0.1872 | 0.087 | 0.031 | 0.001 | 0.0249 | 0.97 |
|  | rs11591741 | 10 | 101976501 | G | 0.58 | Model 3 | -0.0007 | 0.028 | 0.98 | -0.1095 | 0.0721 | 0.13 | 0.1859 | 0.0874 | 0.034 | 0.0014 | 0.025 | 0.95 |
| TM6SF2 | rs2228603 | 19 | 19329924 | T | 0.08 | Model 1 | 0.023 | 0.0485 | 0.64 | 0.1137 | 0.1832 | 0.53 | 0.0758 | 0.2101 | 0.72 | 0.0311 | 0.0458 | 0.5 |
|  | rs2228603 | 19 | 19329924 | T | 0.08 | Model 2 | 0.0205 | 0.0496 | 0.68 | 0.1242 | 0.1843 | 0.5 | 0.102 | 0.2129 | 0.63 | 0.0311 | 0.0467 | 0.51 |
|  | rs2228603 | 19 | 19329924 | T | 0.08 | Model 3 | 0.0134 | 0.0499 | 0.79 | 0.1236 | 0.1853 | 0.5 | 0.0833 | 0.2144 | 0.7 | 0.0238 | 0.047 | 0.61 |
| TM6SF2 | rs58542926 | 19 | 19379549 | T | 0.07 | Model 1 | 0.0347 | 0.0479 | 0.47 | 0.0276 | 0.1132 | 0.81 | 0.2768 | 0.1707 | 0.1 | 0.0488 | 0.0427 | 0.25 |
|  | rs58542926 | 19 | 19379549 | T | 0.07 | Model 2 | 0.031 | 0.049 | 0.53 | 0.0288 | 0.1139 | 0.8 | 0.2787 | 0.1721 | 0.11 | 0.0465 | 0.0435 | 0.29 |
|  | rs58542926 | 19 | 19379549 | T | 0.07 | Model 3 | 0.0211 | 0.0493 | 0.67 | 0.0276 | 0.1143 | 0.81 | 0.2769 | 0.1741 | 0.11 | 0.0382 | 0.0438 | 0.38 |
| PNPLA3 | rs738409 | 22 | 44324727 | G | 0.23 | Model 1 | 0.2138 | 0.0302 | **1.6E-12** | 0.016 | 0.0583 | 0.78 | 0.1396 | 0.0759 | 0.066 | 0.1682 | 0.0253 | **3.0E-11** |
|  | rs738409 | 22 | 44324727 | G | 0.23 | Model 2 | 0.2136 | 0.031 | **5.2E-12** | 0.0168 | 0.0586 | 0.77 | 0.1353 | 0.0766 | 0.078 | 0.1667 | 0.0258 | **1.0E-10** |
|  | rs738409 | 22 | 44324727 | G | 0.23 | Model 3 | 0.209 | 0.0311 | **1.9E-11** | 0.0197 | 0.0588 | 0.74 | 0.1248 | 0.0772 | 0.11 | 0.1628 | 0.0259 | **3.4E-10** |
| PNPLA3 | rs2281135 | 22 | 44332570 | A | 0.17 | Model 1 | 0.1742 | 0.0336 | **2.2E-07** | 0.0606 | 0.0571 | 0.29 | 0.1571 | 0.078 | 0.044 | 0.1464 | 0.0272 | **6.9E-08** |
|  | rs2281135 | 22 | 44332570 | A | 0.17 | Model 2 | 0.1703 | 0.0344 | **7.3E-07** | 0.0637 | 0.0574 | 0.27 | 0.1514 | 0.0788 | 0.055 | 0.1433 | 0.0276 | **2.1E-07** |
|  | rs2281135 | 22 | 44332570 | A | 0.17 | Model 3 | 0.1652 | 0.0346 | **1.8E-06** | 0.0673 | 0.0576 | 0.24 | 0.1445 | 0.0792 | 0.068 | 0.1399 | 0.0278 | **4.6E-07** |
| PNPLA3 | rs2143571 | 22 | 44391686 | A | 0.18 | Model 1 | 0.1174 | 0.0331 | **0.00039** | 0.0312 | 0.0445 | 0.48 | 0.0289 | 0.081 | 0.72 | 0.0811 | 0.0252 | **0.0013** |
|  | rs2143571 | 22 | 44391686 | A | 0.18 | Model 2 | 0.1198 | 0.0339 | **0.0004** | 0.0322 | 0.0447 | 0.47 | 0.0381 | 0.0818 | 0.64 | 0.083 | 0.0256 | **0.0012** |
|  | rs2143571 | 22 | 44391686 | A | 0.18 | Model 3 | 0.1138 | 0.034 | **0.00083** | 0.0364 | 0.0449 | 0.42 | 0.035 | 0.0823 | 0.67 | 0.0806 | 0.0258 | **0.0018** |

**FIB4 score**: Age [years] x AST [U/L] / (platelets [10^9/L] x sqrt (ALT)). **Abbreviations:** rsID: dbSNP identifier (build 151), Chr: chromosome, Pos: basepair position on human genome reference hg19, EA: effect allele, EAF: effect allele frequency among Europeans (Million Veteran Program), Beta: effect size estimated increase in trait per increase copy of the effect allele (additive model). SE: Standard error on Beta. **Model 1:** adjusted for age, gender, and 10 principal components (PCs), **Model 2:** covariates in Model 1 + alcohol consumption at enrollment measured by the Alcohol Use Disorder Identification Test (AUDIT-C), **Model 3:** covariates in Model 2 + Type II diabetes/prediabetes, hypertension, dyslipidemia and BMI ≥ 30 kg/m2. P-values below 0.006 (adjusted for multiple comparisons) are shown in **bold font**.

**Table S7e.** Previously published ALT level-associated/ NAFLD risk variants with genome-wide significance and associations with the **NAFLD fibrosis score** as a continuous measure

|  |  |  |  |  |  |  | **European Ancestry**  **(n=46,653)** | | | **African Ancestry**  **(n=8,019)** | | | **Hispanic/Latino Ancestry**  **(n=5,870)** | | | **Trans-ethnic Meta-Analysis**  **(n=60,542)** | | |
| --- | --- | --- | --- | --- | --- | --- | --- | --- | --- | --- | --- | --- | --- | --- | --- | --- | --- | --- |
| **Gene** | **rsID** | **Chr** | **Pos** | **EA** | **EAF** | **ADJUSTED** | **BETA** | **SE** | **P** | **BETA** | **SE** | **P** | **BETA** | **SE** | **P** | **BETA** | **SE** | **P** |
| LYPLAL1 | rs12137855 | 1 | 219448378 | C | 0.8 | Model 1 | -0.076 | 0.084 | 0.37 | -0.2393 | 0.1888 | 0.2 | -0.8689 | 0.3112 | 0.0053 | -0.1469 | 0.0745 | 0.049 |
|  | rs12137855 | 1 | 219448378 | C | 0.8 | Model 2 | -0.0788 | 0.0866 | 0.36 | -0.2453 | 0.1902 | 0.2 | -0.8877 | 0.3159 | 0.005 | -0.1532 | 0.0765 | 0.045 |
|  | rs12137855 | 1 | 219448378 | C | 0.8 | Model 3 | -0.0661 | 0.0869 | 0.45 | -0.2925 | 0.1903 | 0.12 | -0.8647 | 0.3157 | 0.0062 | -0.1499 | 0.0767 | 0.051 |
| LYPLAL1 | rs3001032 | 1 | 219727779 | T | 0.69 | Model 1 | 0.0555 | 0.0736 | 0.45 | -0.3168 | 0.1434 | 0.027 | -0.2791 | 0.2267 | 0.22 | -0.042 | 0.0629 | 0.5 |
|  | rs3001032 | 1 | 219727779 | T | 0.69 | Model 2 | 0.0607 | 0.0759 | 0.42 | -0.3135 | 0.1443 | 0.03 | -0.2844 | 0.2299 | 0.22 | -0.0411 | 0.0645 | 0.52 |
|  | rs3001032 | 1 | 219727779 | T | 0.69 | Model 3 | 0.0869 | 0.0761 | 0.25 | -0.3049 | 0.1443 | 0.035 | -0.2606 | 0.2301 | 0.26 | -0.0191 | 0.0646 | 0.77 |
| GCKR | rs780094 | 2 | 27741237 | T | 0.4 | Model 1 | 0.0423 | 0.0685 | 0.54 | 0.0578 | 0.1875 | 0.76 | -0.0695 | 0.2339 | 0.77 | 0.0361 | 0.062 | 0.56 |
|  | rs780094 | 2 | 27741237 | T | 0.4 | Model 2 | 0.0476 | 0.0707 | 0.5 | 0.0539 | 0.1888 | 0.78 | -0.0611 | 0.237 | 0.8 | 0.0405 | 0.0637 | 0.53 |
|  | rs780094 | 2 | 27741237 | T | 0.4 | Model 3 | 0.0825 | 0.0709 | 0.24 | 0.0459 | 0.189 | 0.81 | 0.0037 | 0.2371 | 0.99 | 0.0726 | 0.064 | 0.26 |
| HSD17B13 | rs6834314 | 4 | 88213808 | A | 0.72 | Model 1 | 0.2241 | 0.076 | **0.0032** | -0.0903 | 0.1722 | 0.6 | 0.3601 | 0.3148 | 0.25 | 0.1816 | 0.0679 | 0.0075 |
|  | rs6834314 | 4 | 88213808 | A | 0.72 | Model 2 | 0.2284 | 0.0784 | **0.0036** | -0.0879 | 0.1734 | 0.61 | 0.3555 | 0.3192 | 0.27 | 0.1833 | 0.0697 | 0.0085 |
|  | rs6834314 | 4 | 88213808 | A | 0.72 | Model 3 | 0.2311 | 0.0786 | **0.0033** | -0.0661 | 0.1733 | 0.7 | 0.3716 | 0.319 | 0.24 | 0.1896 | 0.0698 | 0.0066 |
| HSD17B13 | rs72613567 | 4 | 88231392 | T | 0.73 | Model 1 | 0.2018 | 0.0765 | 0.0083 | 0.1054 | 0.2854 | 0.71 | 0.2326 | 0.3318 | 0.48 | 0.1971 | 0.0721 | 0.0063 |
|  | rs72613567 | 4 | 88231392 | T | 0.73 | Model 2 | 0.209 | 0.0788 | 0.008 | 0.0943 | 0.2876 | 0.74 | 0.2224 | 0.3368 | 0.51 | 0.202 | 0.0742 | 0.0065 |
|  | rs72613567 | 4 | 88231392 | T | 0.73 | Model 3 | 0.212 | 0.0791 | 0.0073 | 0.1244 | 0.2875 | 0.67 | 0.2525 | 0.3367 | 0.45 | 0.2081 | 0.0744 | **0.0051** |
| PPP1R3B | rs4240624 | 8 | 9184231 | G | 0.09 | Model 1 | -0.1042 | 0.1141 | 0.36 | -0.3985 | 0.1794 | 0.026 | -0.5484 | 0.2839 | 0.054 | -0.2261 | 0.0912 | 0.013 |
|  | rs4240624 | 8 | 9184231 | G | 0.09 | Model 2 | -0.1078 | 0.1178 | 0.36 | -0.3874 | 0.1809 | 0.032 | -0.517 | 0.2878 | 0.073 | -0.2253 | 0.0934 | 0.016 |
|  | rs4240624 | 8 | 9184231 | G | 0.09 | Model 3 | -0.1138 | 0.1181 | 0.34 | -0.3915 | 0.1808 | 0.03 | -0.4719 | 0.2883 | 0.1 | -0.2258 | 0.0935 | 0.016 |
| TRIB1 | rs2954021 | 8 | 126482077 | A | 0.5 | Model 1 | -0.0324 | 0.0673 | 0.63 | -0.0357 | 0.1466 | 0.81 | 0.2052 | 0.2273 | 0.37 | -0.0169 | 0.0591 | 0.78 |
|  | rs2954021 | 8 | 126482077 | A | 0.5 | Model 2 | -0.0436 | 0.0694 | 0.53 | -0.0187 | 0.1476 | 0.9 | 0.2231 | 0.2304 | 0.33 | -0.021 | 0.0606 | 0.73 |
|  | rs2954021 | 8 | 126482077 | A | 0.5 | Model 3 | -0.021 | 0.0697 | 0.76 | -0.0204 | 0.1476 | 0.89 | 0.2329 | 0.2306 | 0.31 | -0.0033 | 0.0608 | 0.96 |
| ERLIN1 | rs10883437 | 10 | 101795361 | T | 0.61 | Model 1 | 0.0423 | 0.0695 | 0.54 | -0.0563 | 0.1424 | 0.69 | 0.2521 | 0.224 | 0.26 | 0.0398 | 0.0602 | 0.51 |
|  | rs10883437 | 10 | 101795361 | T | 0.61 | Model 2 | 0.0447 | 0.0717 | 0.53 | -0.0486 | 0.1433 | 0.73 | 0.2689 | 0.2271 | 0.24 | 0.0439 | 0.0617 | 0.48 |
|  | rs10883437 | 10 | 101795361 | T | 0.61 | Model 3 | 0.0466 | 0.0719 | 0.52 | -0.0594 | 0.1433 | 0.68 | 0.2814 | 0.227 | 0.22 | 0.0443 | 0.0619 | 0.47 |
| ERLIN1 | rs11597390 | 10 | 101861435 | G | 0.64 | Model 1 | -0.0041 | 0.0712 | 0.95 | 0.0437 | 0.1801 | 0.81 | 0.1463 | 0.2334 | 0.53 | 0.0131 | 0.0637 | 0.84 |
|  | rs11597390 | 10 | 101861435 | G | 0.64 | Model 2 | -0.0111 | 0.0735 | 0.88 | 0.0399 | 0.1813 | 0.83 | 0.1618 | 0.2367 | 0.49 | 0.0088 | 0.0654 | 0.89 |
|  | rs11597390 | 10 | 101861435 | G | 0.64 | Model 3 | -0.0101 | 0.0737 | 0.89 | 0.0486 | 0.1812 | 0.79 | 0.1853 | 0.2368 | 0.43 | 0.0126 | 0.0656 | 0.85 |
| ERLIN1 | rs11597086 | 10 | 101953705 | A | 0.58 | Model 1 | 0.0183 | 0.0693 | 0.79 | 0.0002 | 0.2764 | 1 | 0.2752 | 0.2579 | 0.29 | 0.0337 | 0.0651 | 0.6 |
|  | rs11597086 | 10 | 101953705 | A | 0.58 | Model 2 | 0.0117 | 0.0715 | 0.87 | -0.0019 | 0.278 | 0.99 | 0.2992 | 0.261 | 0.25 | 0.0298 | 0.0669 | 0.66 |
|  | rs11597086 | 10 | 101953705 | A | 0.58 | Model 3 | 0.0166 | 0.0717 | 0.82 | 0.0169 | 0.2778 | 0.95 | 0.2829 | 0.2608 | 0.28 | 0.0342 | 0.0671 | 0.61 |
| ERLIN1 | rs11591741 | 10 | 101976501 | G | 0.58 | Model 1 | 0.014 | 0.0693 | 0.84 | -0.0649 | 0.2391 | 0.79 | 0.2345 | 0.2578 | 0.36 | 0.0221 | 0.0645 | 0.73 |
|  | rs11591741 | 10 | 101976501 | G | 0.58 | Model 2 | 0.0075 | 0.0715 | 0.92 | -0.0678 | 0.2405 | 0.78 | 0.2565 | 0.2609 | 0.33 | 0.0179 | 0.0663 | 0.79 |
|  | rs11591741 | 10 | 101976501 | G | 0.58 | Model 3 | 0.0125 | 0.0717 | 0.86 | -0.0551 | 0.2404 | 0.82 | 0.235 | 0.2608 | 0.37 | 0.0217 | 0.0664 | 0.74 |
| TM6SF2 | rs2228603 | 19 | 19329924 | T | 0.08 | Model 1 | -0.0859 | 0.1236 | 0.49 | -0.3577 | 0.6095 | 0.56 | 0.2346 | 0.6263 | 0.71 | -0.0847 | 0.119 | 0.48 |
|  | rs2228603 | 19 | 19329924 | T | 0.08 | Model 2 | -0.101 | 0.1274 | 0.43 | -0.3465 | 0.6137 | 0.57 | 0.2938 | 0.6366 | 0.64 | -0.0962 | 0.1224 | 0.43 |
|  | rs2228603 | 19 | 19329924 | T | 0.08 | Model 3 | -0.1084 | 0.1278 | 0.4 | -0.2747 | 0.6134 | 0.65 | 0.2221 | 0.6366 | 0.73 | -0.1028 | 0.1228 | 0.4 |
| TM6SF2 | rs58542926 | 19 | 19379549 | T | 0.07 | Model 1 | -0.1221 | 0.1221 | 0.32 | -0.234 | 0.3776 | 0.54 | 1.0795 | 0.5096 | 0.034 | -0.0728 | 0.1133 | 0.52 |
|  | rs58542926 | 19 | 19379549 | T | 0.07 | Model 2 | -0.1403 | 0.1258 | 0.26 | -0.2259 | 0.3799 | 0.55 | 1.0855 | 0.5156 | 0.035 | -0.0859 | 0.1163 | 0.46 |
|  | rs58542926 | 19 | 19379549 | T | 0.07 | Model 3 | -0.1572 | 0.1263 | 0.21 | -0.1382 | 0.3797 | 0.72 | 1.0469 | 0.5164 | 0.043 | -0.0939 | 0.1167 | 0.42 |
| PNPLA3 | rs738409 | 22 | 44324727 | G | 0.23 | Model 1 | 0.3717 | 0.077 | **1.4E-06** | -0.0534 | 0.1945 | 0.78 | 0.1241 | 0.2264 | 0.58 | 0.2968 | 0.0683 | **1.4E-05** |
|  | rs738409 | 22 | 44324727 | G | 0.23 | Model 2 | 0.3642 | 0.0794 | **4.5E-06** | -0.0572 | 0.1957 | 0.77 | 0.1095 | 0.2292 | 0.63 | 0.2864 | 0.0701 | **4.3E-05** |
|  | rs738409 | 22 | 44324727 | G | 0.23 | Model 3 | 0.3468 | 0.0796 | **1.3E-05** | -0.089 | 0.1959 | 0.65 | 0.0663 | 0.2297 | 0.77 | 0.2646 | 0.0702 | **0.00017** |
| PNPLA3 | rs2281135 | 22 | 44332570 | A | 0.17 | Model 1 | 0.4083 | 0.0855 | **1.8E-06** | 0.0247 | 0.1902 | 0.9 | 0.062 | 0.2325 | 0.79 | 0.3153 | 0.074 | **2.0E-05** |
|  | rs2281135 | 22 | 44332570 | A | 0.17 | Model 2 | 0.3976 | 0.0882 | **6.5E-06** | 0.0243 | 0.1915 | 0.9 | 0.0471 | 0.2356 | 0.84 | 0.3028 | 0.0758 | **6.5E-05** |
|  | rs2281135 | 22 | 44332570 | A | 0.17 | Model 3 | 0.3851 | 0.0884 | **1.3E-05** | -0.0166 | 0.1917 | 0.93 | 0.0135 | 0.236 | 0.95 | 0.2835 | 0.076 | **0.00019** |
| PNPLA3 | rs2143571 | 22 | 44391686 | A | 0.18 | Model 1 | 0.3044 | 0.0842 | **0.0003** | -0.0241 | 0.148 | 0.87 | 0.2144 | 0.241 | 0.37 | 0.2233 | 0.07 | **0.0014** |
|  | rs2143571 | 22 | 44391686 | A | 0.18 | Model 2 | 0.3039 | 0.0868 | **0.00046** | -0.0327 | 0.1489 | 0.83 | 0.2372 | 0.2442 | 0.33 | 0.2202 | 0.0717 | **0.0021** |
|  | rs2143571 | 22 | 44391686 | A | 0.18 | Model 3 | 0.2899 | 0.087 | **0.00087** | -0.0324 | 0.149 | 0.83 | 0.2013 | 0.2444 | 0.41 | 0.2074 | 0.0718 | **0.0039** |

**NAFLD fibrosis score**: -1.675+(0.037*age)+(0.094*BMI)+(1.13*(diabetes or prediabetes))+(0.99*(AST/ALT))-(0.013*platelets)-(0.66*albumin). **Abbreviations:** rsID: dbSNP identifier (build 151), Chr: chromosome, Pos: basepair position on human genome reference hg19, EA: effect allele, EAF: effect allele frequency among Europeans (Million Veteran Program), Beta: effect size estimated increase in trait per increase copy of the effect allele (additive model). SE: Standard error on Beta. **Model 1:** adjusted for age, gender, and 10 principal components (PCs), **Model 2:** covariates in Model 1 + alcohol consumption at enrollment measured by the Alcohol Use Disorder Identification Test (AUDIT-C), **Model 3:** covariates in Model 2 + Type II diabetes/prediabetes, hypertension, dyslipidemia and BMI ≥ 30 kg/m2. P-values below 0.006 (adjusted for multiple comparisons) are shown in **bold font**.
