# Supplementary figures and images for: Validating a non-invasive, ALT-based non-alcoholic fatty liver phenotype in the million veteran program
Source: PLoS One. 2020 Aug 25;15(8):e0237430. doi: 10.1371/journal.pone.0237430 (PMC7447043; doi:10.1371/journal.pone.0237430)

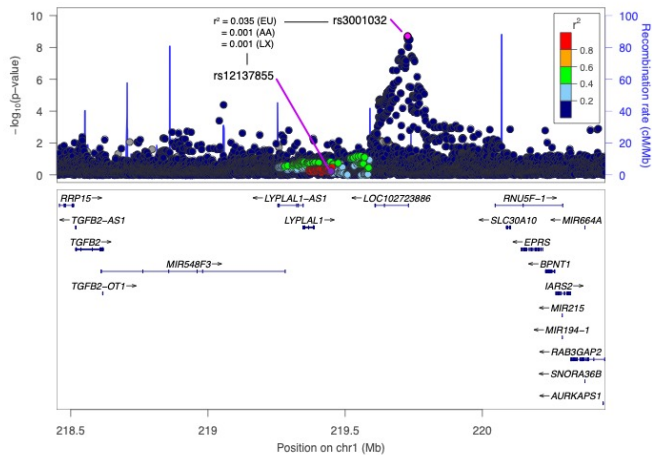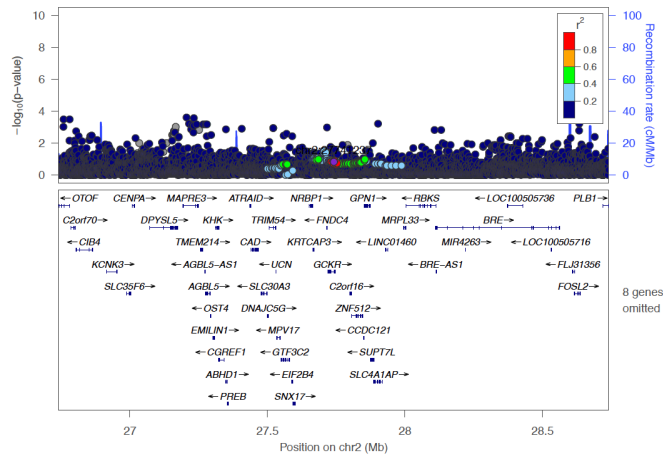

8 genes omitted

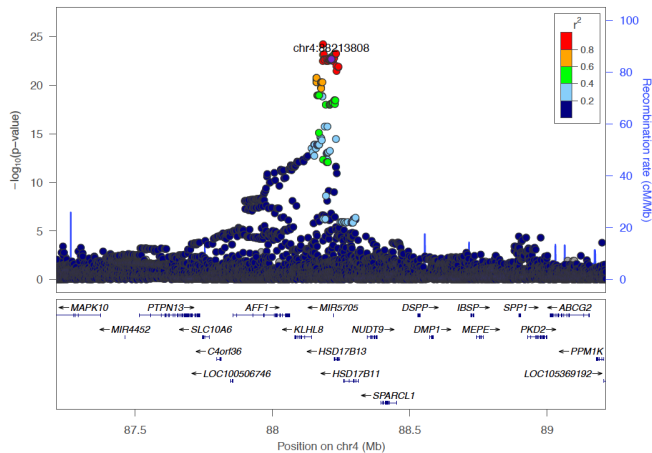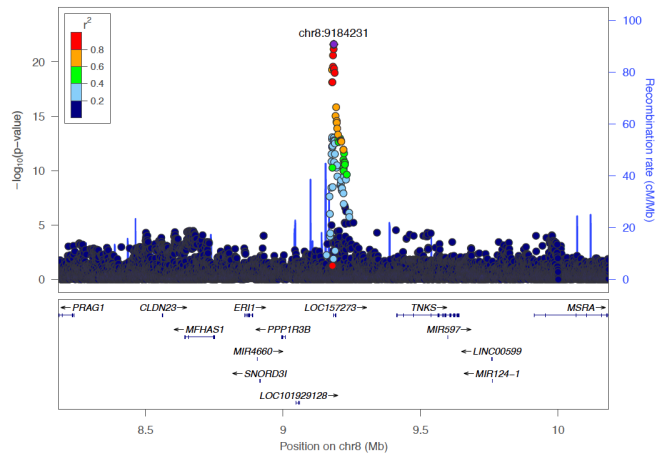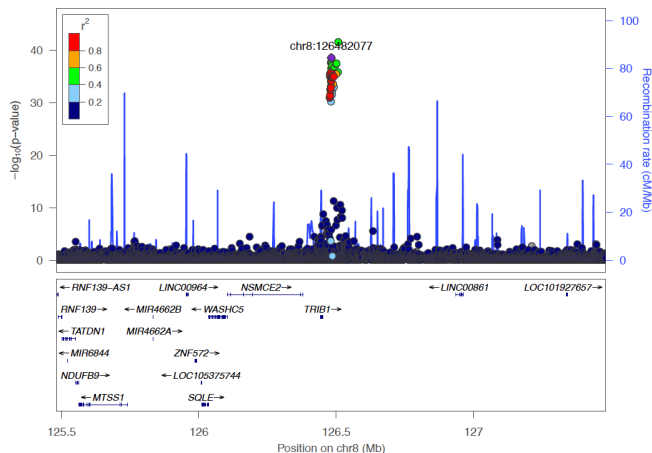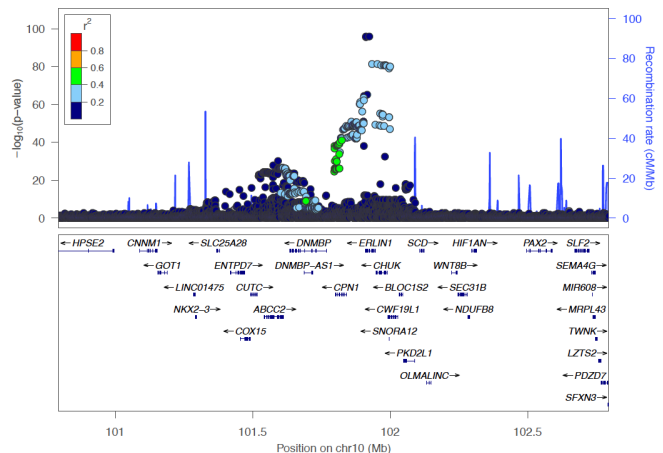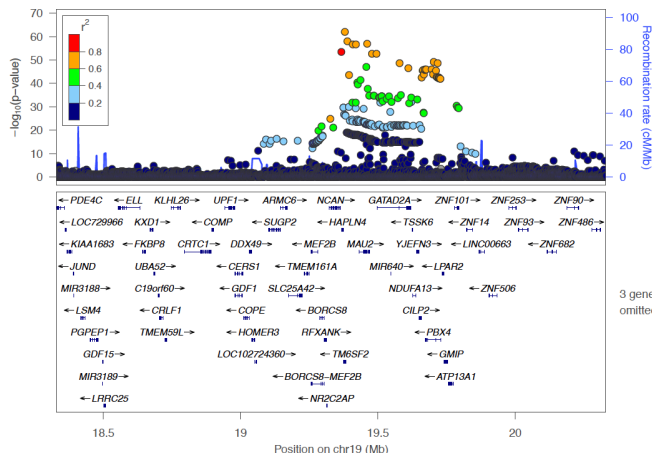

3 genes omitted

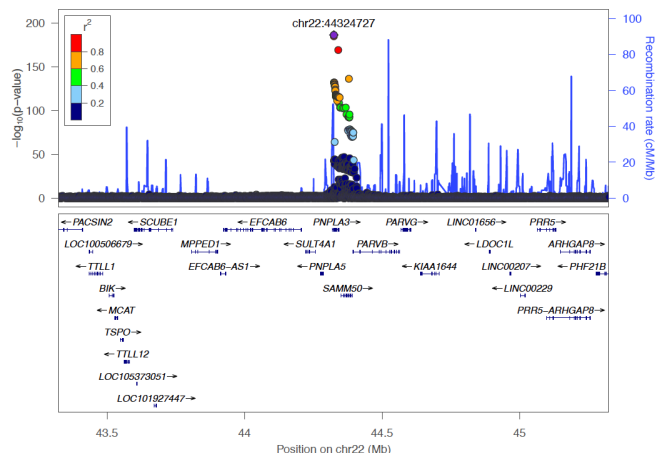

Supplement: S1 Fig — (PDF) [file pone.0237430.s002.pdf]
